# Western medical acupuncture techniques for pain management in athletes: a systematic review and meta-analysis

**DOI:** 10.3389/fmed.2026.1737602

**Published:** 2026-02-17

**Authors:** Karima Chaabna, Anupama Jithesh, Jibrail Cheema, Jasmine Aboughanem, Ravinder Mamtani

**Affiliations:** Institute for Population Health, Weill Cornell Medicine - Qatar, Ar Rayyan, Qatar

**Keywords:** acupuncture effectiveness, evidence-based complementary medicine, musculoskeletal injuries, needle-based therapy, pain management, physical therapy adjuncts, sport medicine

## Abstract

**Background:**

Musculoskeletal pain can undermine athletic performance. Medical procedures that fall under Western medical acupuncture (WMA) such as dry needling, grounded in conventional scientific principles, represent a promising adjunct to conventional pain treatments. However, its effectiveness among athletes remains unclear. To address this gap, we conducted a systematic review and meta-analysis to assess whether WMA reduces pain in athletic populations.

**Methods:**

We searched PubMed, Web of Science, SPORTDiscus, Allied and Complementary Medicine databases, and Google Scholar (latest search: July 2023). We included primary studies that used WMA techniques, including dry needling, manual acupuncture, and percutaneous needle electrolysis, applied based on biomedical principles. Eligible studies diagnosed pain using conventional medical criteria or validated tools and selected evidence-based acupoints based on peer-reviewed research and/or conventional anatomy and physiology, without reference to traditional Asian acupuncture principles. Random-effects meta-analyses were conducted to assess pre–post and between-group changes in pain.

**Results:**

We included 8 studies with overall good internal validity. Publication biases and heterogeneity between studies were identified. In pre–post within-group analyses, WMA techniques alone significantly reduced mean pain scores (number of studies (*n*) = 5, *p*-value = 0.002) whereas WMA techniques combined with exercise and/or physiotherapy showed a nonsignificant reduction (*n* = 3, *p*-value = 0.206). In between-group comparisons, significant decreases in mean pain scores were observed for both WMA techniques alone (*n* = 1, *p*-value = 0.0003) and WMA techniques combined with exercise and/or physiotherapy (*n* = 3, *p*-value = 0.011). The certainty of evidence was rated low for WMA techniques alone and moderate for WMA techniques combined with physiotherapy and/or exercise.

**Conclusion:**

Our findings suggest that WMA techniques alone or combined with physiotherapy and/or exercise may reduce pain among athletes. However, the current evidence base remains preliminary, and additional well-controlled trials are required to establish its efficacy with greater confidence.

**Systematic review registration:**

https://osf.io/qb9gc/overview, identifier osf.io/qb9gc.

## Introduction

Athletes undergo high-frequency and high-intensity training that imposes physical demands that often exceed those experienced by non-athletes. This sustained load can increase the risk of acute musculoskeletal injuries, overuse injuries, and musculoskeletal pain (1, 2), which in turn restrict participation in training (3) and athletic performance (4). Effective strategies for the treatment of musculoskeletal pain are therefore essential to prevent negative impacts on athletes’ performance, careers, and incomes (5).

One strategy to address this challenge is Western medical acupuncture (WMA), an adaptation of traditional Asian acupuncture (6, 7). WMA encompasses multiple techniques, such as dry needling (DN) and ultrasound-guided percutaneous needle electrolysis (PNE). This review collectively refers to these as ‘WMA techniques’. These techniques share the use of needle insertion grounded in biomedical principles but differ in mechanism and application. They select acupuncture points (or acupoints) where the needles are inserted based on anatomy, physiology, pathology, and the principles of evidence-based medicine (8). The insertion of needles at specific acupoints triggers the release of biochemicals, including endorphins (hormones that function as painkillers in the body) (9). As such, WMA techniques represent a promising adjunct to conventional treatments (e.g., analgesics, nonsteroidal anti-inflammatory drugs, cryotherapy, and physical therapy) while offering the advantage of avoiding systemic side effects and drug dependence.

Skepticism surrounding acupuncture in general, within parts of the scientific and medical communities, emerges from the traditional approach in Asian acupuncture, where diagnosis and acupoint selection are according to traditional theories involving the circulation of Qi vital energy (10) and the dynamic balance between Yin and Yang as opposing forces within the body (7). In this context, someone may argue that it lacks empirical foundation (11). While these traditional models hold cultural and historical significance, their metaphysical nature is often perceived as incompatible with contemporary biomedical standards. Although many practicing acupuncturists employ conventional diagnostic methods, the rationale for point selection frequently blends traditional theory with biomedical principles. This conceptual overlap continues to fuel skepticism toward a broader uptake of acupuncture, as it complicates efforts to evaluate acupuncture using standardized, evidence-based methodologies. A focused evaluation of WMA techniques, grounded in biomedical principles, can help reduce conceptual ambiguity, improve clinical clarity, support evidence-based decision-making, and address lingering skepticism.

The effectiveness of acupuncture for pain reduction has been debated in the evidence-based syntheses (3, 5, 12–16). Most systematic reviews (SRs) have either focused exclusively on traditional Asian acupuncture (3) or combined evidence from both WMA and traditional approaches, without distinguishing between them (5, 12–15), making it difficult to isolate the effects of needle-based interventions based purely on anatomical and physiological mechanisms. Additionally, these SRs have primarily examined general or mixed populations. Therefore, the findings of these SRs may not be applicable in the context of pain management among athletes, limiting the relevance to sports medicine.

Given the distinct context of athletes and the increasing use of WMA techniques in sports medicine, a focused evaluation of their effectiveness in this population is needed. To address this gap, we conducted a SR and meta-analysis of randomized clinical trials (RCTs) and observational studies to synthesize evidence exclusively on the effectiveness of WMA techniques for musculoskeletal pain management in athletes. The findings from this review aim to strengthen the evidence base for the use of WMA techniques as a complementary modality in sports medicine.

## Methods

### Protocol and registration

This research protocol was developed *a priori* and prospectively registered on the Open Science Framework (registration number: osf.io/qb9gc). This SR adheres to the Preferred Reporting Items for Systematic reviews and Meta-Analyses (PRISMA) extension for acupuncture (checklists are provided in Supplementary Tables 1–3). The methodology of this SR complies with the Assessment of Multiple Systematic Reviews-2 (AMSTAR-2) tool for critical appraisal of SRs.

### Eligibility criteria

The study eligibility criteria were developed using the Population, Intervention, Comparison, Outcomes, and Study design (PICOS) framework (17).

**Population:** Studies were eligible if they included adult athletes (≥18 years) and/or child athletes (<18 years) who were practicing any sport. Studies that included individuals participating in recreational sports were excluded. No exclusions were made based on geographical coverage.

**Intervention:** The intervention evaluated in this SR was WMA techniques, characterized by needle insertion based on conventional anatomical and physiological considerations. Our selection criteria specifically targeted WMA techniques to assess only intervention with an evidence-based rationale. Primary studies were included if the treatment approach was grounded in biomedical principles and excluded if it referenced traditional Asian medicine concepts (e.g., Qi, meridians, Yin-Yang). Eligible studies employed WMA techniques, including manual acupuncture (MA), PNE, or DN either administered alone or in combination with other interventions (e.g., physical exercise). We excluded studies that combined acupuncture with traditional Asian therapies (e.g., acupressure, cupping, or moxibustion). While all WMA techniques share a common biomedical rationale for acupoint selection, they differ procedurally. Throughout the manuscript, we use the umbrella term ‘WMA techniques’ and specify individual techniques where relevant.

**Comparator:** Studies were included if WMA techniques was compared with any comparator (e.g., sham acupuncture or physical exercise).

**Outcome:** The primary outcome of interest analyzed in this SR was the change in pain intensity, measured by *mean pain scores before and after the intervention* and *mean pain scores between intervention and control groups*. Studies reporting pain outcomes as odds ratios were eligible. We included studies that used pain diagnostic criteria. We included studies that measured the therapeutic effect of WMA techniques (pain intensity) using standards from conventional medicine or validated tools. We excluded studies that relied on measures derived from traditional Asian medicine.

In line with PRISMA guidelines for SRs on acupuncture, we included only studies utilizing terminology from Western medicine (e.g., pain intensity). We excluded studies using terminology from traditional medicine (e.g., syndrome score for syndrome remission) (18). We included primary studies that focused on pain management related to musculoskeletal pain. We excluded primary studies using acupuncture as short-term analgesia for surgical procedures.

Study design: We included clinical trials and observational studies. We excluded animal studies. Articles in languages other than English, Arabic, Spanish, or French (the languages spoken by the research team) were excluded if their English abstracts did not provide sufficient information to address our research questions.

The detailed selection criteria are presented in Supplementary Box 1.

### Information sources and search strategy

We conducted systematic searches across the PubMed/MEDLINE, Web of Science, SPORTDiscus, Allied and Complementary Medicine databases, and Google Scholar covering publications up to 2023. The search strategy employed the keywords *athletes* and *acupuncture*, along with relevant synonyms, across all mentioned databases. The search strategy and choice of databases were reviewed and approved by an experienced librarian. The detailed controlled vocabulary and free-text terms used to search each database are provided in Supplementary Box 2. No language restrictions were applied. Reference lists of included articles and relevant reviews identified during title and abstract screening were manually screened to supplement the search.

### Study selection and data extraction

Removal of duplicates and multistage screening were conducted using Rayyan software (Rayyan Systems Inc., Cambridge, MA, USA^1^). Title and abstract screening were performed independently by two reviewers. The full-text articles were screened independently by three reviewers. All reviewers agreed on the set of articles to include, with any disagreements resolved through discussion among team members. The final list of excluded studies is provided in Supplementary Box 3.

Data extraction was independently conducted by two reviewers using a predesigned extraction sheet developed in Microsoft Excel. The sheet included the following: (i) study characteristics (i.e., study design, sampling method, data collection time, and sample size); (ii) setting; (iii) population description, including age and sex; (iv) details of the intervention; (v) outcome (i.e., mean pain scores, mean pain differences, and pain measurement tool); and (vi) additional items described in the STandards for Reporting Interventions in Clinical Trials of Acupuncture (STRICTA) checklist (19).

The extracted data were compared to identify discrepancies. All extracted data were independently checked for accuracy by a third reviewer. Any disagreements were resolved through discussion among team members.

### Risk of bias assessment

The risk of bias (RoB) and declared conflicts of interest of the included studies were appraised independently by two reviewers. Each RCTs included in this review was assessed for RoB using the Cochrane Collaboration Risk of Bias 1 (RoB-1) tool (20) (Supplementary Table 4). Each RCT was assessed for six sources of bias: (i) sequence generation; (ii) allocation concealment; (iii) blinding of participants and personnel; (iv) blinding of the outcome assessment; (v) incomplete outcome data; and (vi) selective outcome reporting. The RoB for each study was categorized as low, some concerns, high, or unclear.

For observational studies, we used the National Heart, Lung, and Blood Institute (NHLBI) study quality assessment tool for observational cohort and cross-sectional studies (21) (Supplementary Table 5).

When differences in opinion emerged during the RoB assessment, they were discussed to reach consensus. In cases where the articles did not provide sufficient information for a clear determination, we attributed an unclear RoB to the specific source of bias. To ensure a comprehensive evaluation of each study’s methodological quality, we used information from the publications and their supplementary materials and communicated with the study authors when necessary. The final decision for each RoB assessment is reported along with relevant quotes from the study publications to provide transparency and support the rationale behind our assessments.

### Data synthesis

Weighted inverse-variance, random-effects meta-analyses were conducted to estimate pooled differences in mean pain scores: within groups (pre- vs. post-intervention) and between groups (intervention vs. control). If the standard deviation (SD) was not reported, we calculated it from the 95% confidence interval, as recommended by the Cochrane Handbook (22). The inclusion of studies in the meta-analysis is described in Supplementary Table 6 and Supplementary Box 4.

To explore heterogeneity, subgroup meta-analyses were conducted according to intervention type (i.e., WMA techniques alone and with exercise/physiotherapy), pain etiology [i.e., delayed-onset muscle soreness (DOMS), shoulder, elbow, hip, and knee], body section studied (i.e., upper and lower body), needle insertion location (i.e., in muscle and in tendon), and pain measurement tool (i.e., visual analog scale [VAS], numerical pain rating scale [NPRS], and patient-rated elbow evaluation [PREE]). We assessed heterogeneity between studies via the I^2^ statistics, with a cutoff point of ≥50% and a *p* value <0.10 per the chi-squared test, which was defined as a significant level of heterogeneity. Statistical significance was set at *α* = 0.05. Differences in mean scores was considered significant when the 95% confidence interval did not include the null value (zero) (23). Meta-analyses were conducted with R software (version 4.0.0, 64 bit).

Potential small study effects or publication bias were explored via contour-enhanced funnel plots (24). The commonly used Egger’s test was not conducted because each meta-analysis included fewer than 10 studies.

### Certainty of evidence assessment

The certainty of evidence was assessed using the Grading of Recommendations, Assessment, Development, and Evaluation (GRADE) approach (25) in accordance with the Cochrane Handbook.

## Results

### Study selection and characteristics of the included primary studies

**Figure 1:**
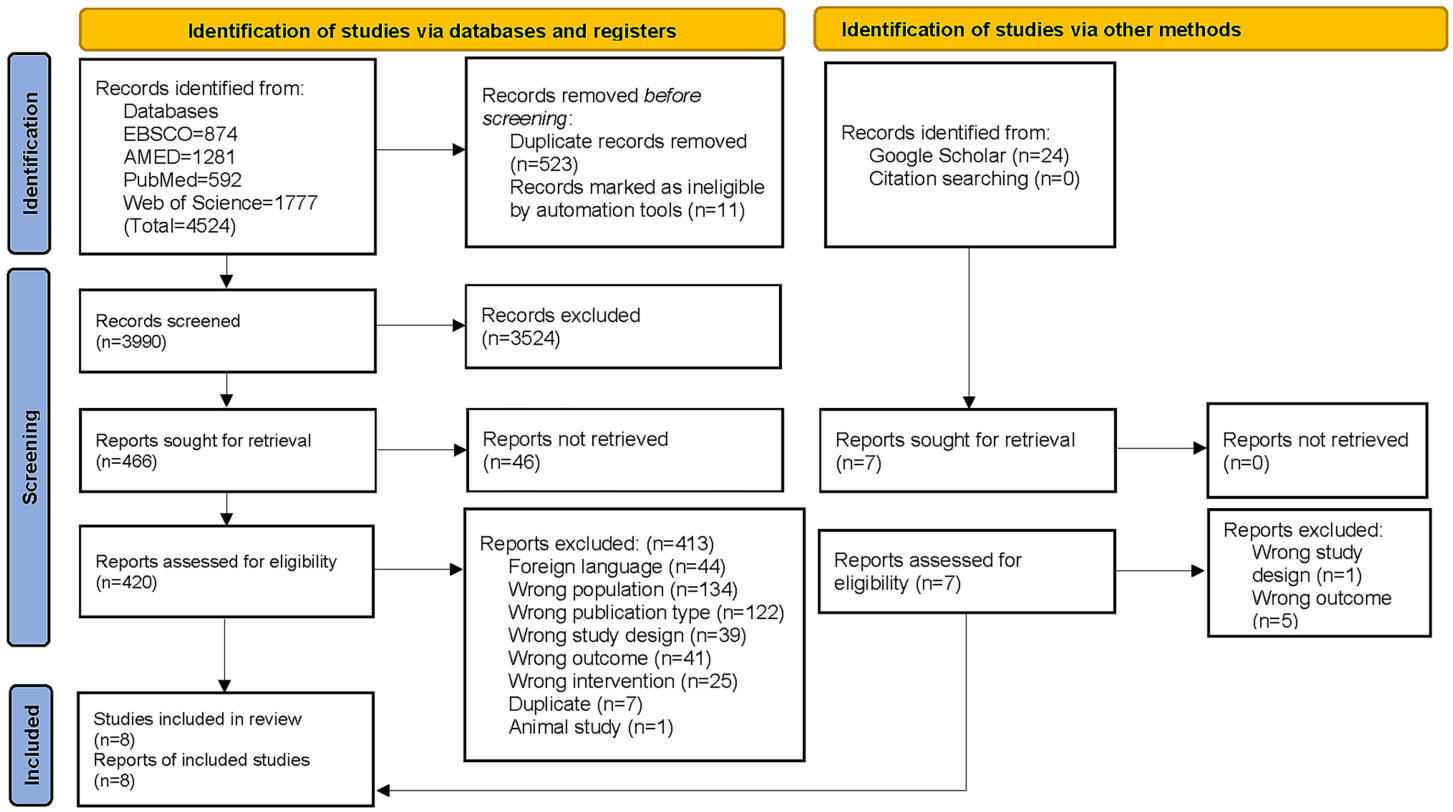
PRISMA flow diagram. Study selection process for the systematic review on the effectiveness of Western medical acupuncture in reducing pain among athletes. A total of 4,524 records were identified through database searches and 24 through Google Scholar. After removing duplicates, screening titles and abstracts, and assessing full texts, 8 studies met the inclusion criteria and were included in the review.

A total of 4,548 records were identified through database and Google Scholar searches (Figure 1). After duplicate removal and title/abstract screening, 473 full-text articles were assessed for eligibility. Of these, 6 RCTs (26–31) and 2 longitudinal studies (32, 33) met the inclusion criteria, for a total of 8 studies included in this SR (26–33). A detailed description of the included studies, according to the STRICTA checklist (19), is provided in Supplementary Tables 6, 7.

The included studies examined adult athletes with elbow (29), shoulder (28, 30), hip (31), or knee (26, 27) pain, and healthy adolescent athletes with DOMS in the muscle groups of the lower limbs (32, 33). The studies evaluated various medical procedures falling within the broader framework of WMA, including DN (27–29, 31), MA (32, 33), ultrasound-guided DN (26, 30), or ultrasound-guided percutaneous needle electrolysis (PNE) (26). WMA techniques were applied either to muscles (27, 28, 30–33) or to tendons (26, 29). Interventions included WMA techniques alone (28, 30, 32, 33), WMA techniques combined with exercise (26, 27), or WMA techniques combined with both exercise and physiotherapy (29, 31). The control interventions varied substantially across studies and included DN applied at acupoints different from those selected for the intervention group (28), exercise (27), exercise combined with physiotherapy (29, 31), no intervention (30), or sham needling (26).

Pain intensity was assessed using different instruments across studies, including the Kujala anterior knee pain scale (27), NPRS (27, 30), PREE (29), and VAS (26, 28, 31–34).

All studies reported mean score differences, and none reported odds ratios. The number of acupuncture sessions ranged from one to nine, with needle sizes ranging from 0.20 × 25 mm to 0.30 × 65 mm. Outcome assessments were conducted between 5 days and 8 weeks post-intervention or averaged across five consecutive daily sessions. Treatment duration ranged from 1 to 4 weeks. Needle insertion depth, reported in only two studies, ranged from 3 mm to 25 mm (32, 33) (Supplementary Tables 7, 8).

### Risk of bias within studies

RoB was assessed to evaluate the internal validity of the included studies and to inform confidence in the meta-analytic findings. All RCTs (6/6, 100%) (26–31) had a low RoB for sequence generation, as they all employed randomization to produce comparable intervention and control groups. All RCTs had a low RoB for selective outcome reporting, as they consistently reported findings on their prespecified outcomes (26–31) (Table 1).

**Table 1:** Risk of bias and conflict of interest in included randomized controlled trials.

Risk of bias assessment	Bias domain	Selection bias		Performance bias	Detection bias	Attrition bias	Reporting bias	Conflict of interest
	Sources of bias	Sequence generation	Allocation concealment	Blinding of participants and personnel	Blinding of outcome assessment	Incomplete outcome data	Selective outcome reporting	
**Studies**	Lopez-Royo (2021) (26)							
	Zarei (2020) (27)							
	Kamali (2019) (28)							
	Etminan (2019) (29)							
	Ceballos-Laita (2021) (30)							
	Jamaly (2018) (31)							

	Unclear risk of bias		Low risk of bias		Minor concerns

This table presents the review authors’ judgments on the risk of bias for each included study, assessed using Cochrane’s Risk of Bias Tool (RoB 1). It also includes information on each study’s declared conflicts of interest.

The RoB for allocation concealment was unclear in most RCTs (4/6, 66.7%) (28–31) because the reports lacked sufficient detail to assess whether intervention allocations could have been anticipated before or during enrollment. Blinding of the researchers performing the intervention was not feasible due to the inherent features of the intervention and was therefore either not applied (26–28, 30) or not reported (29, 31) in any RCT. Blinding of participants and blinding of the outcome assessment were applied in most RCTs (4/6, 66.7%) (26–28, 30). Most RCTs (4/6, 66.7%) had a low risk of attrition bias (26–28, 30).

Both longitudinal studies (2/2, 100%) (32, 33) provided positive answers to most of the signaling questions in the quality assessment tool (8/14, 57.1%) (21), reflecting overall good quality (Table 2). Blinding of participants was applied in all longitudinal studies (2/2, 100%) (32, 33). However, neither blinding of the researchers involved in providing the treatment nor blinding of the assessors was performed in any study. No study reported the participation rate or adjusted for the impact of potential confounding variables on the relationship between WMA techniques and pain reduction.

**Table 2:** Risk of bias and conflict of interest in included observational studies.

Signaling questions of the quality assessment tool for observational cohort and cross-sectional studies		Observational studies	
		Luetmer (2019) (32)	Garlanger (2017) (33)
1	Was the research question or objective in this paper clearly stated?		
2	Was the study population clearly specified and defined?		
3	Was the participation rate of eligible persons at least 50%?		
4	Were all the subjects selected or recruited from the same or similar populations (including the same time period)? Were inclusion and exclusion criteria for being in the study prespecified and applied uniformly to all participants?		
5	Was a sample size justification, power description, or variance and effect estimates provided?		
6	For the analyses in this paper, were the exposure(s) of interest measured prior to the outcome(s) being measured?		
7	Was the timeframe sufficient so that one could reasonably expect to see an association between exposure and outcome if it existed?		
8	For exposures that can vary in amount or level, did the study examine different levels of the exposure as related to the outcome (e.g., categories of exposure or exposure measured as continuous variable)?		
9	Were the exposure measures (independent variables) clearly defined, valid, reliable, and implemented consistently across all study participants?		
10	Was the exposure(s) assessed more than once over time?		
11	Were the outcome measures (dependent variables) clearly defined, valid, reliable, and implemented consistently across all study participants?		
12	Were the outcome assessors blinded to the exposure status of participants?		
13	Was loss to follow-up after baseline 20% or less?		
14	Were key potential confounding variables measured and adjusted statistically for their impact on the relationship between exposure(s) and outcome(s)?		
Conflict of interest disclosure			

	Yes		Not reported		No		Cannot determine

This table presents the review authors’ judgments on each signaling question for the included observational studies, assessed using the NIH NHLBI Study Quality Assessment Tool for Observational Cohort and Cross-Sectional Studies (21). Declared conflicts of interest are also reported.

Overall, the included studies showed low RoB in randomization, outcome reporting, and attrition. However, limitations were noted in allocation concealment, blinding, and the absence of confounding control in observational studies.

### Results of individual studies

For shoulder pain, we identified two good-quality RCTs demonstrating that DN significantly reduced pain in overhead athletes (Supplementary Table 6). The first RCT (30), conducted in volleyball and handball players reported short-term shoulder pain relief after a single session of ultrasound-guided DN applied to the teres major (MD = −3.3, *p* value ≤ 0.001), compared with a minimal change in the control group (MD = −0.4, *p* value = 0.07), resulting in significant between-group difference (*p* < 0.001). The second RCT (28), conducted in semi-elite throwers, swimmers, volleyball, and basketball players found significant pre–post pain reductions after three DN sessions targeting the upper trapezius (intervention) or infraspinatus (control) (MD = −4.7 vs. − 4.8, both *p* value≤ 0.001). Despite differences in target muscles and procedures, both studies suggest that DN reduces shoulder pain in overhead athletes.

For elbow pain, one RCT (29) demonstrated that DN combined with exercise and physiotherapy resulted in significantly greater pain reduction than physiotherapy alone. Between-group differences were significant at the seventh session (*p* value< 0.0001), ninth session (*p-*value = 0.006), and 1 week post-treatment (*p-*value < 0.001). To date, no RCT has isolated the effect of DN alone on elbow pain.

For hip pain, one RCT (31) demonstrated that DN combined with physiotherapy and stretching significantly reduced pain compared to physiotherapy alone (31). Between-group differences were significant after the fifth session (*p-*value < 0.001). To date, no RCT has isolated the effect of DN alone on hip pain.

For knee pain, one RCT (26) conducted in athletes with patellar tendinopathy, compared DN plus exercise and PNE plus exercise to sham needling plus exercise, and found no significant differences between intervention and control groups. However, all three showed a significant improvement in pain after a 22-week intervention (*p-*value ≤ 0.05). The second RCT (26), conducted in female athletes with unilateral prepatellar or retro-patellar pain, reported that that DN combined with exercise produced greater reduction in unilateral prepatellar or retro-patellar pain than exercise alone at weeks 4 and 6 postintervention (*p-*values < 0.001) (27). Methodological heterogeneity in controls and pain measures prevents firm conclusions about the independent effectiveness of DN for knee pain.

For DOMS pain, two longitudinal studies (32, 33) used the same MA protocol and reported statistically significant post-intervention pain reductions among adolescents who received at least one of five treatment days (*p-*values<0.05). However, the clinical relevance of these reductions remains uncertain, as no minimal detectable change has been established for the VAS in DOMS (32).

### Pre–post within-group meta-analyses

**Figure 2:**
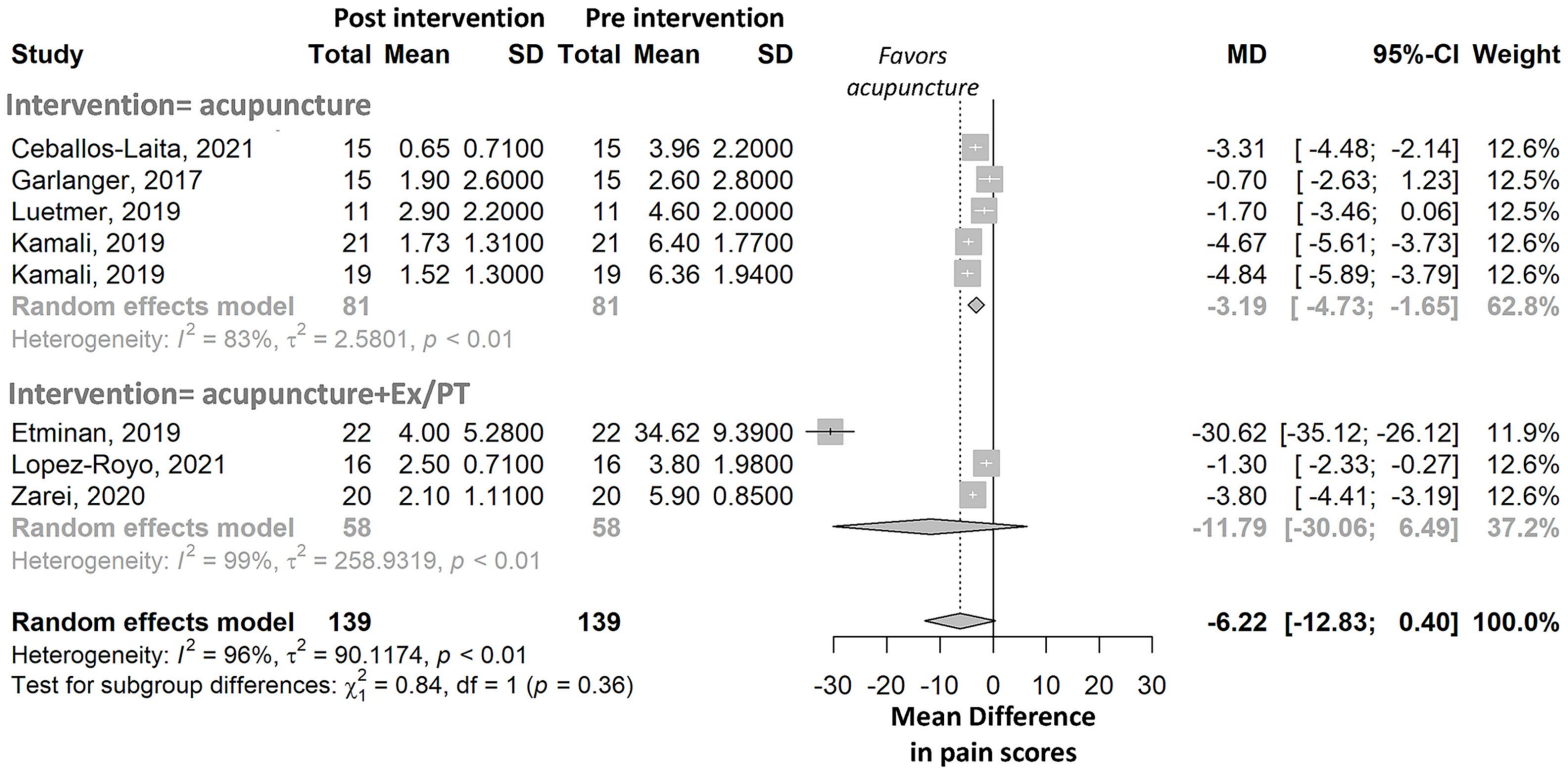
Meta-analysis of pre- to post-intervention differences in pain scores among athletes, stratified by intervention type. Forest plot shows pooled mean differences (MD) with 95% confidence intervals for studies using acupuncture alone or combined with exercise/physiotherapy (Ex/PT). The control group in Kamali, 2019, received dry needling in a shoulder muscle, and this was different from the method selected for the intervention group (Supplementary Table 6 and Supplementary Box 3). Both groups were included in the meta-analysis.

To examine within-group changes in pain, we conducted meta-analyses comparing mean pain scores pre- and post-intervention. WMA techniques alone was associated with a significant reduction in the mean pain score [meta-analysis, number of studies (*n*) = 5, *p-*value = 0.002]. In contrast, WMA techniques combined with exercise and/or physiotherapy resulted in a nonsignificant reduction in the mean pain score (meta-analysis, *n* = 3, *p-*value = 0.206, forest plot in Figure 2). Both WMA techniques and WMA techniques combined with exercise and/or physiotherapy showed high heterogeneity in treatment effects, with *I*^2^ values >50%.

Pre-post intervention differences in pooled mean scores varied significantly according to pain etiology, pain measurement instrument, and the number of sessions (*p* values<0.0001, forest plots in Supplementary Figures 1–3). No significant difference in the reduction of mean pain scores was observed based on the body section treated or the needle insertion location (forest plots in Supplementary Figures 4, 5).

Forest plot shows pooled mean differences (MD) with 95% confidence intervals for studies using WMA techniques alone or combined with exercise/physiotherapy (Ex/PT). The control group in Kamali, 2019, received dry needling in a shoulder muscle, and this was different from the method selected for the intervention group (Supplementary Table 6 and Supplementary Box 4). Intervention and control groups from Kamali, 2019 were included in the pre- to post meta-analysis.

### Between-group intervention vs. control meta-analyses

**Figure 3:**
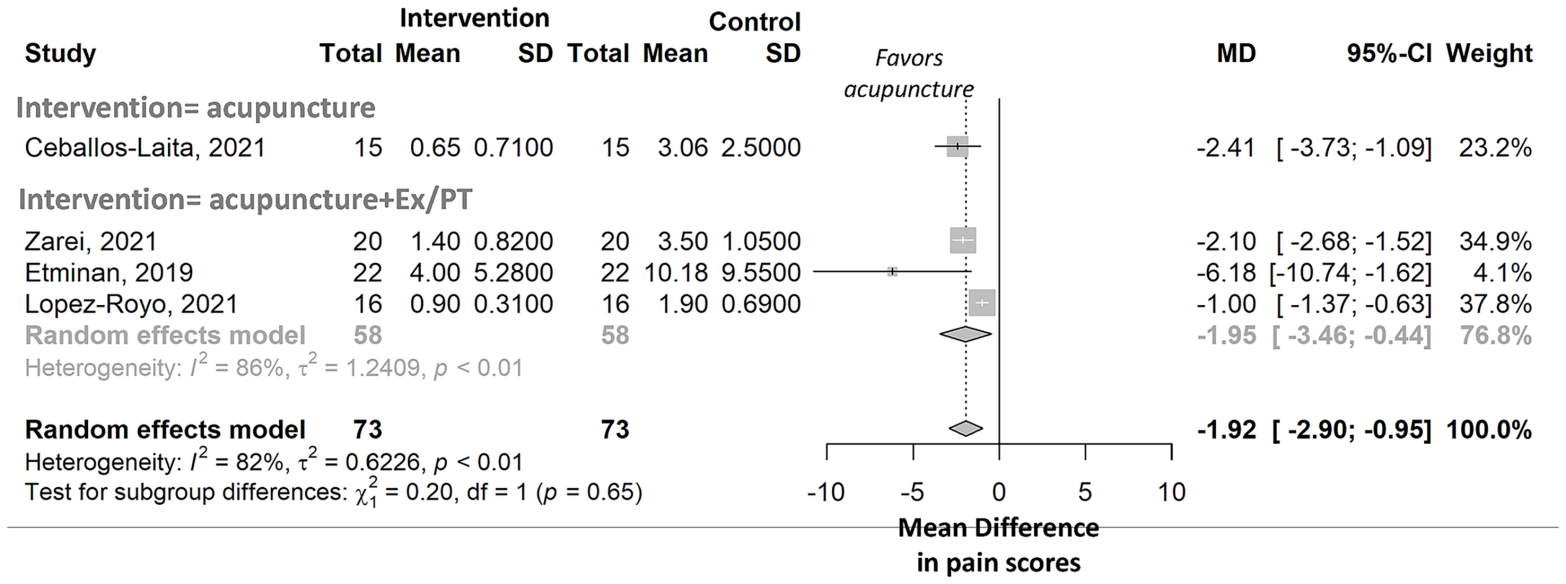
Meta-analysis of between-group differences in pain scores among athletes, stratified by type of intervention. Forest plot shows pooled mean differences (MD) with 95% confidence intervals comparing acupuncture to control groups. Results are stratified by intervention type: acupuncture alone versus acupuncture combined with exercise and/or physiotherapy (Ex/PT). The standardized mean difference was not computed because variability between RCTs was not solely due to the measurement tool used. Standardization assumes that differences in SDs across studies are due to differences in measurement scales rather than actual variability among study populations (22).

We next examined between-group differences in pain by comparing WMA techniques interventions with control conditions. WMA techniques alone (*n* = 1, *p-*value = 0.0003) (30) and WMA techniques combined with exercise and/or physiotherapy (meta-analysis, n = 3, p value = 0.011) were associated with significant reductions in mean pain scores compared with control groups (forest plot in Figure 3). The meta-analysis of WMA techniques combined with exercise and/or physiotherapy revealed high heterogeneity in treatment effects, with an *I*^2^ value of 86%. Differences in pooled mean scores between the intervention and control groups varied significantly according to the type of control intervention (*p-*value = 0.001), pain measurement instrument (*p-*value = 0.0003), and number of sessions (*p-*value < 0.0001; forest plots in Supplementary Figures 6–8). No significant decreases in mean pain scores were observed based on the intervention type, pain etiology, body section treated, or needle insertion location (forest plots in Supplementary Figures 9, 11).

Forest plot shows pooled mean differences (MD) with 95% confidence intervals comparing WMA techniques alone with control groups. Results are stratified by intervention type: WMA techniques alone versus WMA techniques combined with exercise and/or physiotherapy (Ex/PT). The standardized mean difference was not computed because variability between RCTs was not solely attributable to the measurement tool used. Standardization assumes that differences in SDs across studies are due to differences in measurement scales rather than actual variability among study populations (22).

### Reporting bias

Publication bias was assessed using contour-enhanced funnel plots. In the pre–post intervention meta-analyses, the distribution of effect sizes showed asymmetry consistent with small-study effects (Supplementary Figure 12). In the intervention versus control analyses, asymmetry was also observed, suggesting that smaller or nonsignificant studies may be underrepresented (Supplementary Figure 13).

### Certainty of evidence

The certainty of evidence was rated based on changes in pain intensity, the primary outcome of interest. Across the included studies, we identified five types of musculoskeletal pain: elbow pain, shoulder pain, hip pain, knee pain, and DOMS. While, the meta-analysis of five pre–post comparisons showed a statistically significant reduction in mean pain scores, the effectiveness of WMA techniques compared with a control group was evaluated in only one RCT, which showed a significant between-group difference (30). Consequently, the overall certainty of evidence was downgraded due to limited data, clinical and statistical heterogeneity, potential publication bias, and the risk of performance bias—an inherent issue when performing acupuncture interventions. Based on these factors, the certainty of evidence was rated as low for WMA techniques alone, and moderate for WMA techniques combined with physiotherapy and/or exercise. Overall, the available evidence suggests that WMA techniques may offer therapeutic benefit for managing musculoskeletal pain in athletes but confidence in the findings varies depending on the intervention type and study limitations.

## Discussion

In this SR and meta-analysis, we examined the available evidence on the effectiveness of WMA techniques for musculoskeletal pain management in athletes. Although the number of eligible studies was limited, our results suggest that WMA techniques may provide therapeutic benefits for managing musculoskeletal pain in athletes. However, the current evidence base remains preliminary, as high heterogeneity and methodological limitations warrant further well-controlled trials to establish efficacy with greater confidence.

Our work analyzed the effect of WMA techniques (primarily DN, PNE, and MA protocols) on musculoskeletal pain across five different types of pain: shoulder pain, elbow pain, hip pain, knee pain, and DOMS. WMA techniques were associated with reductions in musculoskeletal pain, particularly in shoulder and DOMS pain. For shoulder pain, two good-quality RCTs reported significant pain reduction among overhead athletes following DN, with consistent results despite differences in target muscles and intervention protocols. For DOMS, two longitudinal studies using identical MA protocols reported statistically significant reductions in pain; however, the clinical relevance of these findings remain uncertain (32, 33). However, for elbow, hip, and knee pain, the evidence was less consistent, with some trials reporting significant improvements and others showing comparable outcomes between intervention and control groups. For elbow pain, one trial found that DN combined with physiotherapy and exercise significantly outperformed physiotherapy alone, although the independent effect of DN remains unclear (29). Similarly, for hip pain, one trial showed that DN combined with physiotherapy and stretching significantly reduced pain compared to physiotherapy alone, though the contribution of DN alone could not be determined (31). Evidence for knee pain was inconsistent: one study showed no added benefit of DN or PNE when combined with exercise compared to sham needling (26), while another reported superior pain reduction with DN plus exercise relative to exercise alone (27). These differences likely reflect methodological variations in control groups and pain assessment tools. Overall, the evidence was most consistent for shoulder pain in overhead athletes and DOMS, but further research is needed for other musculoskeletal pain conditions.

Our meta-analysis results provided further nuance: in the pre–post within-group analyses, WMA techniques alone were associated with a statistically significant reduction in pain, whereas WMA techniques combined with exercise and/or physiotherapy produced a nonsignificant reduction. To further explore the potential benefits of WMA techniques we conducted additional between-group comparisons. These analyses examined whether WMA techniques alone or WMA techniques combined with exercise and/or physiotherapy produced greater reductions in pain relative to control groups. Both intervention types showed significant pain reductions compared with controls, suggesting that WMA techniques can play a beneficial role in managing musculoskeletal pain. However, the evidence for WMA techniques alone was derived from a single RCT (30), underscoring the need for replication in future studies to confirm this effect. Additionally, meta-analysis findings should be interpreted with caution, as the high heterogeneity observed (*I*^2^ > 50%) suggests substantial variability across studies, likely driven by differences in pain etiology, measurement tools, and treatment session frequency. The overall certainty of evidence remained limited due to several methodological constraints. Consequently, the certainty of evidence was rated as low for WMA techniques alone and moderate for WMA techniques combined with physiotherapy and/or exercise. Together, these findings suggest a potentially beneficial role for WMA techniques in managing pain, but additional well-controlled trials are necessary to establish its efficacy with greater confidence.

Previous SRs on musculoskeletal pain and sports-related injuries have focused on traditional Asian acupuncture (3) or have aggregated data from both WMA and traditional Asian acupuncture approaches (5, 13–16). While these reviews suggest that acupuncture may offer short-term pain relief for conditions such as chronic low back pain, hand-and-wrist pain and knee osteoarthritis, the overall level of evidence was rated as low to moderate. This is likely due to methodological heterogeneity including variability in acupuncture techniques and control interventions (13–16). In the context of DOMS, findings in published SRs were similarly inconsistent and constrained by small sample sizes and methodological limitations (3, 12). Moreover, these reviews have generally targeted heterogenous general populations, rather than focusing on athlete-specific cohorts (3, 12–16), thereby limiting their relevance to sports medicine. To date only one SR has synthesized case reports involving adult athletes, and it combined both WMA and traditional Asian acupuncture approaches for treating sports-related injuries (5). This SR provides the first meta-analytic synthesis focused on the application of WMA techniques for musculoskeletal pain in athletic populations, offering a targeted and clinically relevant evidence base for sports medicine practitioners. By including only studies that used evidence-based diagnostic criteria, anatomically and physiologically justified acupoint selection, and validated outcome measures, this review adheres closely to the principles of conventional medical practice. This approach minimizes conceptual overlap with traditional techniques and aligns the methodology with biomedical standards.

Our literature search was conducted in July 2023. While AMSTAR-2 considers reviews outdated after 24 months, this standard is more applicable to rapidly evolving fields (e.g., artificial intelligence, oncology, or COVID-19–related research). In contrast, WMA techniques for pain in athletes is a relatively slower-developing field with limited new publications and high methodological variability. Therefore, even if newer studies were published after July 2023, they are unlikely to substantially alter the evidence base summarized in this review. Instead, our review underscores the need for more standardized, conceptually clear, and methodologically rigorous research to advance the field.

The studies included in this review represent an important foundation for advancing research on WMA in the management of musculoskeletal pain among athletes. However, the next step for this field requires greater conceptual clarity and methodological refinement. One key issue concerns the inconsistent of the term *athlete* has not been clearly defined in across studies, w which may limit comparability and reduces the generalizability of findings (35). Establishing a clear and widely accepted scientific definition of *athlete* would enhance the interpretability and relevance of future research (5). Our analyses also revealed clinical and methodological heterogeneity across studies, including the type of intervention (WMA techniques alone vs. WMA techniques combined with exercise and/or physiotherapy), the type of control intervention, the number of acupuncture sessions, the pain etiology, and the measurement instrument used in both the pre- vs. postintervention and control analyses. Furthermore, most studies did not report the depth of needle insertion, a parameter known to influence stimulation intensity and treatment effectiveness (36). Lack of standardized treatment duration and timing for post-treatment pain assessment further complicated the comparison and synthesis of findings across trials. To enhance the quality, comparability, and reproducibility of future research, studies should adopt standardized reporting frameworks (e.g., STRICTA), specify intervention protocols in detail, and use validated outcome measures. Greater methodological rigor, standardization, and comprehensive reporting will be essential to generate reliable evidence, which strengthens the evidence base and guides the effective integration of WMA into sports medicine practice. Further research should explore the long-term clinical impact and economic value of WMA to clarify its role within sports medicine and integrative care models.

## Conclusion

This SR and meta-analysis examined preliminary and methodologically diverse evidence on the effectiveness of WMA techniques in reducing musculoskeletal pain among athletes. While the number of eligible studies was limited, the review provides preliminary evidence and important foundation for guiding future research. Our SR shows that WMA techniques, when used alone or with physiotherapy and/or exercise, reduce musculoskeletal pain in athletic populations. Owing to its well-established safety profile, WMA techniques should be considered as complementary approaches in musculoskeletal pain management in athletes. To support integration of acupuncture in sports medicine, future trials should employ rigorous, standardized designs and report intervention protocol as per reporting guidelines. Additional rigorous research on the effectiveness of WMA techniques is essential to validate their role in managing musculoskeletal.

## Data Availability

The original contributions presented in the study are included in the article/Supplementary material, further inquiries can be directed to the corresponding author/s.

## Footnotes

1
https://www.rayyan.ai/
