# Supplementary material for: Western medical acupuncture techniques for pain management in athletes: a systematic review and meta-analysis
Source: Front Med (Lausanne). 2026 Feb 17;13:1737602. doi: 10.3389/fmed.2026.1737602 (PMC12953514; doi:10.3389/fmed.2026.1737602)
Supplement: Supplementary file 1 [file Data_Sheet_1.docx]

#

# **Western Medical Acupuncture Effectiveness in Pain Management among Athletes: A Systematic Review and Meta-Analysis**

Supplementary file- Table of contents

| eTable 1 | PRISMA-A checklist for acupuncture |
| --- | --- |
| eTable 2 | PRISMA 2020 for Abstracts Checklist |
| eTable 3 | PRISMA-S Checklist |
| eBox 1 | Detailed selection criteria based on the PICOS framework |
| eBox 2 | Search strategy |
| eTable 4 | Quality assessment for Randomized controlled trials |
| eTable 5 | Quality assessment for Observational, cohort and case-control studies |
| eTable 6 | Included studies characteristics and study selection for the meta-analyses |
| eBox 3 | Detailed selection criteria for the meta-analysis |
| eTable 7 | Characteristics of the studies included in the systematic review |
| eTable 8 | Acupuncture effectiveness reported in the included studies |
| eBox 4 | List of excluded studies |
| eFigure 1 | Meta-analysis for the assessment of the pooled pre-to-post intervention difference in pain mean scores (MD) difference by pain etiology |
| eFigure 2 | Meta-analysis for the assessment of the pooled pre-to-post intervention difference in pain mean scores (MD) difference by pain measurement instrument |
| eFigure 3 | Meta-analysis for the assessment of the pooled pre-to-post intervention difference in pain mean scores (MD) difference by number of sessions |
| eFigure 4 | Meta-analysis for the assessment of the pooled pre-to-post intervention difference in pain mean scores (MD) difference by body section studied |
| eFigure 5 | Meta-analysis for the assessment of the pooled pre-to-post intervention difference in pain mean scores (MD) difference by needle insertion location |
| eFigure 6 | Funnel plot for the pre-post mean difference meta-analysis |
| eFigure 7 | Meta-analysis for the assessment of the pooled difference in mean scores (MD) between the intervention vs. the control groups by type of control intervention |
| eFigure 8 | Meta-analysis for the assessment of the pooled difference in mean scores (MD) between the intervention vs. the control groups by pain measurement instrument |
| eFigure 9 | Meta-analysis for the assessment of the pooled difference in mean scores (MD) between the intervention vs. the control groups by number of sessions |
| eFigure 10 | Meta-analysis for the assessment of the pooled difference in mean scores (MD) between the intervention vs. the control groups by pain etiology |
| eFigure 11 | Meta-analysis for the assessment of the pooled difference in mean scores (MD) between the intervention vs. the control groups by body section studied |
| eFigure 12 | Meta-analysis for the assessment of the pooled difference in mean scores (MD) between the intervention vs. the control groups by needle insertion location |
| eFigure 13 | Funnel plot for the meta-analysis of mean difference between the intervention and control groups |
| eBOX 5 | Supplementary Reference |

## eTable 1: PRISMA-A checklist for acupuncture

| **Subjects** | **PRISMA for Acupuncture** | | **Page** |
| --- | --- | --- | --- |
| ***Title*** |  |  |  |
| **Title** | 1^*^ Identify the report as a systematic review, meta-analysis, or both; if applicable, state the specific type of acupuncture treatment, such as manual acupuncture or electroacupuncture. | | Title |
| ***Abstract*** |  |  |  |
| **Structured summary** | 2^†^ Provide a structured summary including, as applicable: background; objectives; data sources; study eligibility criteria, participants, and interventions; study appraisal and synthesis methods; results limitations; conclusions and implications of key findings; systematic review registration number. | | Abstract |
| ***Introduction*** |  |  |  |
| **Rationale** | 3^*^ Describe the rationale for what is already known about acupuncture for the target condition in the background; if applicable, state what is already known about the specific types of acupuncture to be studied, and describe whether there is any difference of the effects among different types of acupuncture. | | Introduction |
| **Objectives** | 4^†^ Provide an explicit statement of questions being addressed with reference to participants, interventions, comparisons, outcomes, and study design (PICOS) | | Introduction and eBOX1 in supplementary file |
| ***Methods*** |  |  |  |
| **Protocol and registration** | 5^†^ Indicate if a review protocol exists, if and where it can be accessed (e.g., web address), and, if available, provide registration information including registration number. | | Protocol and registration |
| **Eligibility criteria** | 6^†^ Specify study characteristics (e.g., PICOS, length of follow-up) and report characteristics (e.g., years considered, language, publication status) used as criteria for eligibility, giving rationale.  6a.1^‡^ Describe the diagnostic criteria of the target condition in Western medicine.  6a.2^‡^ If applicable, describe the diagnostic criteria in terms of Traditional Medicine, such as Traditional Chinese Medicine.  6b^‡^ Describe the types of acupuncture to be included, such as traditional acupuncture, electroacupuncture, or fire acupuncture.  6c^‡^ If applicable, report measures for therapeutic effects using the terminology of either traditional medicine (e.g. syndrome score for syndrome remission) or Western medicine (e.g. pain intensity). | | Eligibility criteria |
| **Information sources** | 7^*^ Describe all sources of information (e.g., databases with dates of coverage, contact with study authors to identify additional studies) in the search, and report the date of the last search. If applicable, report the databases or complementary search methods for acupuncture or traditional medicine. | | Information sources and search strategy and eBOX2 in supplementary file |
| **Search** | 8^*^ Present full electronic search strategy for at least one commonly used database (e.g. MEDLINE), including any limits used, such that it could be repeated. If applicable, include the full search strategy for at least a Western and a traditional medicine database for each systematic review where both were used. | | eBOX2 in supplementary file |
| **Study selection** | 9^†^ State the process for selecting studies (i.e., screening, eligibility, included in systematic review, and, if applicable, included in the meta-analysis). | | Study selection and data extraction |
| **Data collection**  **process** | 10^†^ Describe method of data extraction from reports (e.g., piloted forms, independently, in duplicate) and any processes for obtaining and confirming data from investigators. | | Study selection and data extraction |
| **Data items** | 11^*^ List and define all variables for which data were sought (e.g., PICOS, funding sources) and any assumptions and simplifications made; describe data items about details of acupuncture interventions and controls (**e.g.,** sham acupuncture) referring to TIDieR when applicable. | | Study selection and data extraction |
| **Risk of bias in**  **individual studies** | 12^†^ Describe methods used for assessing risk of bias of individual studies (including specification of whether this was done at the study or outcome level), and how this information is to be used in any data synthesis. | | Risk of bias assessment |
| **Summary measures** | 13^†^ State the principal summary measures (e.g., risk ratio, difference in means). | | Data synthesis |
| **Synthesis of results** | 14^†^ Describe the methods of handling data and combining results of studies, if done, including measures of consistency (e.g., I^2^) for each meta-analysis. | | Data synthesis |
| **Risk of bias across**  **studies** | 15^†^ Specify any assessment of risk of bias that may affect the cumulative evidence (e.g., publication bias, selective reporting within studies). | | Data synthesis |
| **Additional analyses** | 16^†^ Describe methods of additional analyses (e.g., sensitivity or subgroup analyses, meta-regression), if done, indicating which were pre-specified. | | Data synthesis |
| ***Results*** |  |  |  |
| **Study selection** | 17^†^ Give numbers of studies screened, assessed for eligibility, and included in the review, with reasons for exclusions at each stage, ideally with a flow diagram. | | Figure 1; Study selection and characteristics of included studies |
| **Study characteristics** | 18* For each study, present characteristics that were extracted (e.g., study size, PICOS, follow-up period) and provide the citations of the included studies. Summarize details of the acupuncture intervention for each study in a table referring to TIDieR**.**  18a^‡^ Describe details of “De-qi” after acupuncture reported in the included studies. | | Study selection and characteristics of included studies; eTable6 in supplementary file |
| **Risk of bias within**  **studies** | 19^†^ Present data on risk of bias of each study and, if available, any outcome-level assessment (see item 12). | | Risk of bias within studies; Table 1& Table 2 |
| **Results of individual**  **studies** | 20^†^ For all outcomes considered (benefits or harms), present, for each study: (a) simple summary data for each intervention group and (b) effect estimates and confidence intervals, ideally with a forest plot. | | Results of individual studies |
| **Synthesis of results** | 21^†^ Present results of each meta-analysis done, including confidence intervals and measures of consistency. | | Results of individual studies; eFigures 2-14 in supplement file |
| **Risk of bias across**  **studies** | 22^†^ Present results of any assessment of risk of bias across studies (see item 15). | | Reporting bias |
| **Additional analysis** | 23^†^ Give results of additional analyses, if done (e.g., sensitivity or subgroup analyses, meta-regression [see item 16]). | | Certainty of evidence |
| ***Discussion*** |  |  |  |
| **Summary of evidence** | 24^†^ Summarize the main findings including the strength of evidence for each main outcome; consider their relevance to key groups (e.g., health care providers, users, and policy  makers). | | Discussion |
| **Limitations** | 25^†^ Discuss limitations at study and outcome level (e.g., risk of bias), and at review level (e.g., incomplete retrieval of identified research, reporting bias). | | Discussion |
| **Conclusions** | 26^†^ Provide a general interpretation of the results in the context of other evidence, and implications for future research. | | Discussion |
| ***Funding*** |  |  |  |
| **Funding** | 27^†^ Describe sources of funding for the systematic review and other support (e.g., supply of data); role of funders for the systematic review. | | Funding |

Note: * modified original item ^†^ unmodified item from PRISMA ^‡^ new extended item

From: Wang X, Chen Y, Liu Y, Yao L, Estill J, Bian Z, et al. Reporting items for systematic reviews and meta-analyses of acupuncture: the PRISMA for acupuncture checklist. BMC Complementary and Alternative Medicine. 2019 Aug 12;19(1):208. doi: 10.1186/s12906-019-2624-3.

For more information: https://prisma-statement.org/

## eTable 2: PRISMA 2020 for Abstracts Checklist

| **Section and Topic** | **Item #** | **Checklist item** | **Reported (Yes/No)** |
| --- | --- | --- | --- |
| **TITLE** | | |  |
| Title | 1 | Identify the report as a systematic review. | Y |
| **BACKGROUND** | | |  |
| Objectives | 2 | Provide an explicit statement of the main objective(s) or question(s) the review addresses. | Y |
| **METHODS** | | |  |
| Eligibility criteria | 3 | Specify the inclusion and exclusion criteria for the review. | Y |
| Information sources | 4 | Specify the information sources (e.g. databases, registers) used to identify studies and the date when each was last searched. | Y |
| Risk of bias | 5 | Specify the methods used to assess risk of bias in the included studies. | Y |
| Synthesis of results | 6 | Specify the methods used to present and synthesise results. | Y |
| **RESULTS** | | |  |
| Included studies | 7 | Give the total number of included studies and participants and summarise relevant characteristics of studies. | Y |
| Synthesis of results | 8 | Present results for main outcomes, preferably indicating the number of included studies and participants for each. If meta-analysis was done, report the summary estimate and confidence/credible interval. If comparing groups, indicate the direction of the effect (i.e. which group is favoured). | Y |
| **DISCUSSION** | | |  |
| Limitations of evidence | 9 | Provide a brief summary of the limitations of the evidence included in the review (e.g. study risk of bias, inconsistency and imprecision). | Y |
| Interpretation | 10 | Provide a general interpretation of the results and important implications. | Y |
| **OTHER** | | |  |
| Funding | 11 | Specify the primary source of funding for the review. | Y |
| Registration | 12 | Provide the register name and registration number. | Y |

From*:*  Page MJ, McKenzie JE, Bossuyt PM, Boutron I, Hoffmann TC, Mulrow CD, et al. The PRISMA 2020 statement: an updated guideline for reporting systematic reviews. BMJ 2021;372:n71. doi: 10.1136/bmj.n71

For more information, visit: <http://www.prisma-statement.org/>

## eTable 3: PRISMA-S Checklist

| **Section/topic** | **#** | **Checklist item** | **Location(s) Reported** |
| --- | --- | --- | --- |
| **INFORMATION SOURCES AND METHODS** | | | |
| Database name | 1 | Name each individual database searched, stating the platform for each. | 5 |
| Multi-database searching | 2 | If databases were searched simultaneously on a single platform, state the name of the platform, listing all of the databases searched. | 5 and eBOX2 in supplementary file |
| Study registries | 3 | List any study registries searched. | None |
| Online resources and browsing | 4 | Describe any online or print source purposefully searched or browsed (e.g., tables of contents, print conference proceedings, web sites), and how this was done. | eBOX2 in supplementary file |
| Citation searching | 5 | Indicate whether cited references or citing references were examined, and describe any methods used for locating cited/citing references (e.g., browsing reference lists, using a citation index, setting up email alerts for references citing included studies). | 5 |
| Contacts | 6 | Indicate whether additional studies or data were sought by contacting authors, experts, manufacturers, or others. | None |
| Other methods | 7 | Describe any additional information sources or search methods used. | None |
| **SEARCH STRATEGIES** | | | |
| Full search strategies | 8 | Include the search strategies for each database and information source, copied and pasted exactly as run. | eBOX2 in supplementary file |
| Limits and restrictions | 9 | Specify that no limits were used, or describe any limits or restrictions applied to a search (e.g., date or time period, language, study design) and provide justification for their use. | 5 |
| Search filters | 10 | Indicate whether published search filters were used (as originally designed or modified), and if so, cite the filter(s) used. | None |
| Prior work | 11 | Indicate when search strategies from other literature reviews were adapted or reused for a substantive part or all of the search, citing the previous review(s). | None |
| Updates | 12 | Report the methods used to update the search(es) (e.g., rerunning searches, email alerts). | eBOX2 in supplementary file |
| Dates of searches | 13 | For each search strategy, provide the date when the last search occurred. | eBOX2 in supplementary file |
| **PEER REVIEW** | | | |
| Peer review | 14 | Describe any search peer review process. | eBOX2 in supplementary file |
| **MANAGING RECORDS** | | | |
| Total Records | 15 | Document the total number of records identified from each database and other information sources. | Figure1 in main manuscript |
| Deduplication | 16 | Describe the processes and any software used to deduplicate records from multiple database searches and other information sources. | 5 |
| PRISMA-S: An Extension to the PRISMA Statement for Reporting Literature Searches in Systematic Reviews. Rethlefsen ML, Kirtley S, Waffenschmidt S, Ayala AP, Moher D, Page MJ, Koffel JB, PRISMA-S Group.  Last updated February 27, 2020. | | | |

## eBox 1: Detailed selection criteria based on the PICOS framework

**Population:** Studies on athletes including adults and children were included while studies on individuals who were practicing recreational sports were excluded. No exclusion was made based on geographical coverage.

**Intervention:** Western medical acupuncture—defined as a therapeutic modality involving needles penetrating and puncturing the skin at specific points selected based on evidence—was the intervention evaluated in this systematic review^1^. Western medical acupuncture involves the insertion of needles at specific trigger points identified based on anatomy, physiology, pathology, and evidence-based medicine^2^. Western medical acupuncture is adapted from the Chinese traditional acupuncture, which involves the insertion of needles at specific trigger points^3^. Healthcare professionals practicing Western medical acupuncture diagnose and treat health conditions based on evidence-based medicine and do not apply concepts from the Chinese traditional acupuncture like Qi circulation and the Yin/Yang^1^. Therefore, distinguishing between these two types of acupuncture is essential when synthesizing and reporting data^4^. Primary studies investigating the effect of acupuncture interventions like manual acupuncture (MA), electroacupuncture (EA), or dry needling (DN) either alone or in combination with other interventions (e.g., physical exercise) were eligible. However, acupuncture intervention combined with Chinese medicine therapies like acupressure, cupping, or moxibustion were excluded. Primary studies using interventions without skin penetration such as transcutaneous electrical nerve stimulation (TENS), laser acupuncture, and acupressure were also excluded.

**Outcome:** Change in pain intensity was the primary outcome of interest. Primary studies on pain management due to musculoskeletal disease—broadly defined as pain affecting the muscles, ligaments, tendons, or bones—following injury or DOMS (after a training or a competition) were included. Studies reporting other acupuncture use among athletes (e.g., performance improvement) were excluded. Primary studies on acupuncture for short-term analgesia associated with surgical procedures were not included. Primary studies reporting efficacy of acupuncture in reducing pain as mean score differences or odds ratios were eligible—pain level being measured with a validated tool.

**Control**

Studies that compared acupuncture interventions with any comparator (e.g., sham acupuncture or physical exercise) were included.

**Study design:** Studies that compared acupuncture interventions with any comparator (e.g., sham acupuncture or physical exercise) were included. Observational study and clinical trials were included. Articles in languages other than English, Arabic, Spanish, and French (languages spoken by the research team) were excluded if their abstract in English did not contain enough information to address our research questions.

## eBox 2: Search strategy

Database selection was conducted in consultation with an experienced librarian and included two general bibliographic databases (Medline via PubMed and Web of Science) and two specialized databases [SPORTDiscus via EBSCO, Allied and Complimentary Medicine (AMED) via OVID]. Additionally, Google Scholar was searched for grey and non-grey literature. Literature search for eligible articles was carried out electronically from database inception. The latest search was conducted in February 2022. The search strategy and search terms were finalized with the assistance of an experienced librarian. The search terms included controlled vocabulary terms, free-text terms related to the following three component: intervention (acupuncture), outcome (pain), and population (athletes).

The reference lists of relevant published systematic reviews and the publications of the included primary studies were hand searched to ensure that there were no missing studies. The literature search was not limited to any language, country, or study design.

**PubMed/Medline: 493**

**Date search was run: 27 April 2021**

("athletes"[MeSH Terms] OR “Para-Athletes" [MeSH Terms] OR "sports"[MeSH Terms] OR “Athletic Injuries”[MeSH] OR "athletes"[Title/Abstract] OR "athlete"[Title/Abstract] OR "athletically"[Title/Abstract] OR "athlets"[Title/Abstract] OR "sport"[Title/Abstract] OR "sport medicine” [Title/Abstract] OR "sport injuries” [Title/Abstract] OR "sports"[Title/Abstract] OR "sports medicine” [Title/Abstract] OR "sports injury” [Title/Abstract] OR "sports injuries” [Title/Abstract] OR "athletic"[Title/Abstract] OR "athletics"[Title/Abstract] OR "football” [Title/Abstract] OR "soccer” [Title/Abstract] OR "basketball” [Title/Abstract] OR "bicycling” [Title/Abstract] OR "boxing” [Title/Abstract] OR "golf” [Title/Abstract] OR "gymnastic*” [Title/Abstract] OR "Runner*” [Title/Abstract] OR "jogg*” [Title/Abstract] OR "marathon*” [Title/Abstract] OR "volleyball” [Title/Abstract] OR "weight lifting” [Title/Abstract] OR "swimm*” [Title/Abstract])

AND

(“acupuncture” [MeSH] OR “acupuncture therapy” [Mesh:NoExp] OR “Acupuncture, Ear” [MeSH] OR “acupuncture points” [MeSH] OR “Acupuncture Analgesia” [MeSH] OR “electroacupuncture” [MeSH] OR “acupuncture” [Title/Abstract] OR “Pharmacopuncture” [Title/Abstract] OR “electroacupuncture” [Title/Abstract] OR “electro-acupuncture” [Title/Abstract] OR “dry needling” [Title/Abstract] OR “trigger point” [Title/Abstract] OR “acupoint*” [Title/Abstract] OR Acupressure [Title/Abstract])

**OVID/AMED: 1174**

**Date search was run: 27 April 2021**

**(**Rehabilitation/ or Athletic injuries/ or Exercise/ or Injuries/ or Athletes/ or athletes.mp. or para-athletes.mp. or Sports/ or sport.mp. or Football/ or sport medicine.mp. or sport injuries.mp. or sports injury.mp. or sports injuries.mp. or athletic.mp. or athletics.mp. or Soccer/ or soccer.mp. or basketball.mp. or Bicycling/ or bicycling.mp. or boxing.mp. or Boxing/ or Golf/ or golf.mp. or Gymnastics/ or gymnastic.mp. or Running/ or runner.mp. or Jogging/ or jogging.mp. or Walking/ or Diseases in athletes/ or marathon.mp. or volleyball.mp. or Lifting/ or weight lifting.mp. or Swimming/ or swimming.mp.)

AND

(acupuncture.mp. or Acupuncture/ or Acupuncture therapy/ or Acupoints/ or acupuncture therapy.mp. or electroacupuncture.mp. or Electroacupuncture/ or Pharmacopuncture.mp. or electro-acupuncture.mp. or Needling/ or dry needling.mp. or trigger point.mp. or acupoint.mp or acupoints.mp. or Acupoints/ or Acupressure/ or Ear acupuncture/ or Neck pain/ or hand acupuncture.mp.)

**Web of Science: 1461**

**Date search was run: 27 April 2021**

(TS=((athletes) OR (athlete) OR (athletically) OR (sport) OR (sport medicine) OR (sport injuries) OR (sports) OR (sports medicine) OR (sports injury) OR (sports injuries) OR (athletic) OR (athletics) OR (football) OR (soccer) OR (basketball) OR (bicycling) OR (boxing) OR (golf) OR (gymnastic) OR (Running) OR (jogging) OR (marathon) OR (volleyball) OR (weight lifting) OR (swimming)))

AND

(TS=((acupuncture) OR (Pharmacopuncture) OR (electroacupuncture) OR (electro-acupuncture) OR (dry needling) OR (trigger point) OR (acupoint) OR (Acupressure)))

**EBSCO/SportDISCUS: 874**

**Date search: 28 April 2021**

((acupuncture) OR (Pharmacopuncture) OR (electroacupuncture) OR (electro-acupuncture) OR (dry needling) OR (trigger point) OR (acupoint) OR (Acupressure)) OR (DE "ACUPUNCTURE" OR DE "ELECTROACUPUNCTURE" OR DE "acupressure" OR DE "ACUTAPING")

AND

(athletes) OR (athlete) OR (athletically) OR (sport) OR (sport medicine) OR (sport injuries) OR (sports) OR (sports medicine) OR (sports injury) OR (sports injuries) OR (athletic) OR (athletics) OR (football) OR (soccer) OR (basketball) OR (bicycling) OR (boxing) OR (golf) OR (gymnastic) OR (Running) OR (jogging) OR (marathon) OR (volleyball) OR (weight lifting) OR (swimming)

AND

(((((DE "DISEASES in athletes" OR DE "ATHLETES" OR DE "ABORIGINAL Australian athletes" OR DE "AFRICAN athletes" OR DE "AMATEUR athletes" OR DE "ARAB athletes" OR DE "ARCHERS" OR DE "ASIAN athletes" OR DE "ATHLETES as actors" OR DE "ATHLETES in art" OR DE "ATHLETES with disabilities" OR DE "BADMINTON players" OR DE "BASEBALL players" OR DE "BASKETBALL players" OR DE "BLACK athletes" OR DE "BOBSLEDDERS" OR DE "BODYBUILDERS" OR DE "BOWLERS" OR DE "BOXERS (Sports)" OR DE "BULLFIGHTERS" OR DE "CANADIAN athletes" OR DE "CANOEISTS" OR DE "CELEBRITY athletes" OR DE "CHILD athletes" OR DE "CHILDREN of athletes" OR DE "CHRISTIAN athletes" OR DE "COLLEGE athletes" OR DE "CRICKET players" OR DE "CROQUET players" OR DE "CURLERS (Athletes)" OR DE "CYCLISTS" OR DE "DEFENSIVE players" OR DE "DIABETIC athletes" OR DE "ELITE athletes" OR DE "ENDURANCE athletes" OR DE "EUROPEAN athletes" OR DE "FENCERS" OR DE "FOOTBALL players" OR DE "GAY athletes" OR DE "GLADIATORS" OR DE "GOLFERS" OR DE "GYMNASTS" OR DE "HANDBALL players" OR DE "HIGH school athletes" OR DE "HOCKEY players" OR DE "INTERSEX athletes" OR DE "JAI alai players" OR DE "JEWISH athletes" OR DE "JUNIOR high school athletes" OR DE "KABADDI players" OR DE "LACROSSE players" OR DE "LAWN bowlers" OR DE "LGBTQ+ athletes" OR DE "LONG-term athlete development" OR DE "MALE athletes" OR DE "MARTIAL artists" OR DE "MEXICAN athletes" OR DE "MIDDLE school athletes" OR DE "MOUNTAINEERS" OR DE "MUSLIM athletes" OR DE "NATIVE American athletes" OR DE "NETBALL players" OR DE "OFFENSIVE players" OR DE "OLDER athletes" OR DE "OLYMPIC athletes" OR DE "ORIENTEERS" OR DE "PACIFIC Islander athletes" OR DE "PROFESSIONAL athletes" OR DE "ROWERS" OR DE "RUGBY football players" OR DE "RUNNERS (Sports)" OR DE "SKATERS" OR DE "SKIERS" OR DE "SKYDIVERS" OR DE "SNOWBOARDERS" OR DE "SOCCER players" OR DE "SOFTBALL players" OR DE "SQUASH players" OR DE "STARTING players" OR DE "SUBSTITUTE players" OR DE "SURFERS" OR DE "SWIMMERS" OR DE "TABLE tennis players" OR DE "TEAM handball players" OR DE "TENNIS players" OR DE "TRACK & field athletes" OR DE "TRIATHLETES" OR DE "VOLLEYBALL players" OR DE "WATER polo players" OR DE "WEIGHT lifters" OR DE "WINDSURFERS (Persons)" OR DE "WOMEN athletes" OR DE "WRESTLERS") AND (DE "ATHLETES with disabilities" OR DE "AUTISTIC athletes" OR DE "BASEBALL players with disabilities" OR DE "BASKETBALL players with disabilities" OR DE "BLIND athletes" OR DE "DEAF athletes" OR DE "SKIERS with disabilities" OR DE "WOMEN athletes with disabilities" OR DE "ATHLETES' health" OR DE "WOMEN athletes' health")) OR (DE "ATHLETIC ability" OR DE "ATHLETIC ability testing")) OR (DE "ATHLETICS" OR DE "AMATEUR sports" OR DE "ATHLETIC tryouts" OR DE "BAG punching" OR DE "BOXING" OR DE "COLLEGE sports" OR DE "DUATHLON" OR DE "FENCING" OR DE "GOODWILL Games" OR DE "GYMNASTICS" OR DE "HIGHLAND games" OR DE "JIU-jitsu" OR DE "MIXED martial arts" OR DE "PANCRATIUM" OR DE "PARKOUR" OR DE "POWERLIFTING" OR DE "PROFESSIONALISM in sports" OR DE "SENIOR Olympics" OR DE "SKATING" OR DE "SWIMMING" OR DE "TETRATHLON" OR DE "TRACK & field" OR DE "TRIATHLON" OR DE "WALKING" OR DE "WEIGHT lifting" OR DE "WRESTLING")) OR (DE "SPORTS" OR DE "AERODYNAMICS in sports" OR DE "AERONAUTICAL sports" OR DE "AGE & sports" OR DE "AMATEUR sports" OR DE "ANIMAL sports" OR DE "ANTISEMITISM in sports" OR DE "AQUATIC sports" OR DE "BALL games" OR DE "BALLISTICS in sports" OR DE "BASEBALL" OR DE "BIOMECHANICS in sports" OR DE "COLLEGE sports" OR DE "COMBAT sports" OR DE "COMMUNICATION in sports" OR DE "CONTACT sports" OR DE "CROSS-training (Sports)" OR DE "DISC golf" OR DE "DISCRIMINATION in sports" OR DE "DOG sports" OR DE "DOPING in sports" OR DE "ENDURANCE sports" OR DE "EXTREME sports" OR DE "FANTASY sports" OR DE "FASCISM & sports" OR DE "FEMINISM & sports" OR DE "GAELIC games" OR DE "GAY Games" OR DE "GOODWILL Games" OR DE "GYMNASTICS" OR DE "HOCKEY" OR DE "HOMOPHOBIA in sports" OR DE "HYDRODYNAMICS in sports" OR DE "INDIVIDUAL sports" OR DE "KINEMATICS in sports" OR DE "KNIFE throwing" OR DE "LGBTQ+ people & sports" OR DE "LOG-chopping (Sports)" OR DE "MASCULINITY in sports" OR DE "MASS media & sports" OR DE "MILITARY sports" OR DE "MINORITIES in sports" OR DE "MOTION pictures in sports" OR DE "MOTORSPORTS" OR DE "NATIONAL socialism & sports" OR DE "NATIONALISM & sports" OR DE "NONVERBAL communication in sports" OR DE "OLYMPIC Games" OR DE "PARKOUR" OR DE "PHYSICS in sports" OR DE "PRESIDENTS -- Sports" OR DE "PROFESSIONAL sports" OR DE "PROFESSIONALISM in sports" OR DE "RACISM in sports" OR DE "RACKET games" OR DE "RADAR in sports" OR DE "RECREATIONAL sports" OR DE "REGIONALISM & sports" OR DE "ROBOTICS in sports" OR DE "RODEOS" OR DE "ROLLER skating" OR DE "SCHOOL sports" OR DE "SENIOR Olympics" OR DE "SEXUAL harassment in sports" OR DE "SHOOTING (Sports)" OR DE "SHUTOUTS (Sports)" OR DE "SOCIALISM & sports" OR DE "SOFTBALL" OR DE "SPORT for all" OR DE "SPORTS & state" OR DE "SPORTS & technology" OR DE "SPORTS & theater" OR DE "SPORTS & tourism" OR DE "SPORTS for children" OR DE "SPORTS for girls" OR DE "SPORTS for older people" OR DE "SPORTS for people with disabilities" OR DE "SPORTS for youth" OR DE "SPORTS forecasting" OR DE "SPORTS in antiquity" OR DE "SPORTS penalties" OR DE "SPORTS photography" OR DE "SPORTS rivalries" OR DE "SPORTS teams" OR DE "SPORTS tourism" OR DE "STEREOTYPES in sports" OR DE "TARGETS (Sports)" OR DE "TEAM sports" OR DE "TEAMWORK (Sports)" OR DE "TELEVISION & sports" OR DE "TRACEURS" OR DE "VIDEO tapes in sports" OR DE "VIOLENCE in sports" OR DE "WINTER sports" OR DE "WOMEN'S sports")) OR (DE "SPORTS injuries" OR DE "ACHILLES tendinitis" OR DE "AEROBICS injuries" OR DE "AQUATIC sports injuries" OR DE "BASEBALL injuries" OR DE "BASKETBALL injuries" OR DE "BOXING injuries" OR DE "COMMOTIO cordis" OR DE "CRICKET injuries" OR DE "DELAYED onset muscle soreness" OR DE "EQUESTRIAN accidents" OR DE "FOOTBALL injuries" OR DE "GOLF injuries" OR DE "GYMNASTICS injuries" OR DE "HIKING injuries" OR DE "HOCKEY injuries" OR DE "HORSE sports injuries" OR DE "IN-line skating injuries" OR DE "JOGGING injuries" OR DE "JUDO injuries" OR DE "JUMPER'S knee" OR DE "KARATE injuries" OR DE "MARTIAL arts injuries" OR DE "MOTORSPORTS injuries" OR DE "NETBALL injuries" OR DE "RACKET game injuries" OR DE "RUGBY football injuries" OR DE "RUNNING injuries" OR DE "SKATEBOARDING injuries" OR DE "SOCCER injuries" OR DE "TENNIS injuries" OR DE "TURF toe" OR DE "VAULTING injuries" OR DE "VOLLEYBALL injuries" OR DE "WALKING (Sports) injuries" OR DE "WEIGHT training injuries" OR DE "WINTER sports injuries")

**Google Scholar**

**Date search was run: 14 February 2022**

Pain acupuncture

Search results:100

Pain acupuncture football

Search results:100

Pain acupuncture soccer

Search results:100

Pain acupuncture basketball

Search results:100

Pain acupuncture bicycling

Search results:100

Pain acupuncture boxing

Search results:100

Pain acupuncture runner

Search results:100

Pain acupuncture golf

Search results:100

Pain acupuncture gymnastic

Search results:100

Pain acupuncture marathon

Search results:100

Pain acupuncture swimming

Search results:100

Pain acupuncture volleyball

Search results:100

Pain acupuncture weight lifting

Search results:100

Dry needling athlete

Search results:100

## **eBox 3: List** of excluded studies

1. Acosta-Olivo C, Siller-Adame A, Tamez-Mata Y, Vilchez-Cavazos F, Pena-Martinez V, Acosta-Olivo C. Laser Treatment on Acupuncture Points Improves Pain and Wrist Functionality in Patients Undergoing Rehabilitation Therapy after Wrist Bone Fracture. A Randomized, Controlled, Blinded Study. Acupunct Electrother Res. 2017;42(1):11-25.

2. Adams AC, Fleming KB, Tripp PM. Hip Pain in a Collegiate Gymnast. International Journal of Athletic Therapy & Training. 2018;23(4):178-80.

3. Adams Z, Stewart R, Taylor A, Scifers JR. Dry Needling. Athletic Training & Sports Health Care: The Journal for the Practicing Clinician. 2020;12(2):53-5.

4. Ahlgren C, Waling K, Kadi F, Djupsjobacka M, Thornell LE, Sundelin G. Effects on physical performance and pain from three dynamic training programs for women with work-related trapezius myalgia. J Rehabil Med. 2001;33(4):162-9.

5. Al-Boloushi Z, Gomez-Trullen EM, Arian M, Fernandez D, Herrero P, Bellosta-Lopez P. Comparing two dry needling interventions for plantar heel pain: a randomised controlled trial. BMJ Open. 2020;10(8):e038033.

6. Alaei P, Ansari NN, Naghdi S, Fakhari Z, Komesh S, Dommerholt J. Dry Needling for Hamstring Flexibility: A Single-Blind Randomized Controlled Trial. Journal of Sport Rehabilitation. 2021;30(3):452-7.

7. Allais G, De Lorenzo C, Quirico PE, Lupi G, Airola G, Mana O, et al. Non-pharmacological approaches to chronic headaches: transcutaneous electrical nerve stimulation, lasertherapy and acupuncture in transformed migraine treatment. Neurol Sci. 2003;24 Suppl 2:S138-42.

8. Amano K, Sakai T, Katayama K, Katsumi Y. Evaluation of the effects of acupuncture treatment on jumper's knee. ;1995 1995.

9. Ammer K. Trigger point therapy for carpal tunnel syndrome. Manuelle Medizin. 2010;48(6):465-7.

10. An XX. Galvao acupuncture for treating athletes' muscular sprain. Journal of Shanghai Physical Education Institute / Shanghai Tiyu Xueyuan Xuebao. 1997;21(4):53-5;65.

11. Andersson S, Sundberg T, Johansson E, Falkenberg T. Patients' experiences and perceptions of integrative care for back and neck pain. Altern Ther Health Med. 2012;18(3):25-32.

12. Ansari NN, Alaei P, Naghdi S, Fakhari Z, Komesh S, Dommerholt J. Immediate Effects of Dry Needling as a Novel Strategy for Hamstring Flexibility: A Single-Blinded Clinical Pilot Study. Journal of Sport Rehabilitation. 2020;29(2):156-61.

13. Arbuthnot H. Acupuncture and Trigger Point Needling in Track and Field. Modern Athlete & Coach. 2010;48(4):16-8.

14. Arbuthnot H. Acupuncture. Modern Athlete & Coach. 2012;50(2):16-7.

15. Archer PA. Three clinical sports massage approaches for treating injured athletes. Athletic Therapy Today. 2001;6(3):14-20.

16. Arias-Buria J, Valero-Alcaide R, Cleland J, Salom-Moreno J, Ortega-Santiago R, Atin-Arratibel M. Inclusion of trigger point dry-needling in a multimodal physical therapy program for postoperative shoulder pain: A randomised controlled trial. New Zealand Journal of Physiotherapy. 2016;44(2):113.

17. Arias-Buria JL, Valero-Alcaide R, Cleland JA, Salom-Moreno J, Ortega-Santiago R, Atin-Arratibel MA, et al. Inclusion of trigger point dry needling in a multimodal physical therapy program for postoperative shoulder pain: a randomized clinical trial. J Manipulative Physiol Ther. 2015;38(3):179-87.

18. arlene Cimons M. Pinning Down Pain. Runner's World. 1999;34(3):36.

19. Arriaga-Pizano L, Gomez-Jimenez DC, Flores-Mejia LA, Perez-Cervera Y, Solorzano-Mata CJ, Lopez-Macias C, et al. Low back pain in athletes can be controlled with acupuncture by a catecholaminergic pathway: clinical trial. Acupuncture in Medicine. 2020;38(6):388-95.

20. Atchison J, Taub N, Cotter A, Tellis A. Complementary and alternative medicine treatments for low back pain. Physical Medicine and Rehabilitation: State of the Art Reviews. 1999;13(3):561-86.

21. Bahrami-Taghanaki H, Liu Y, Azizi H, Khorsand A, Esmaily H, Bahrami A, et al. A randomized, controlled trial of acupuncture for chronic low-back pain. Altern Ther Health Med. 2014;20(3):13-9.

22. Baoqiang D, Yu HAN, Honglin W, Chunri LI. Clinical Evaluation on Treatment of Knee Osteoarthritis with Knee Rehabilitation Gymnastics. Journal of Shenyang Sport University. 2011;30(3):77-9.

23. Baraja-Vegas L, Martin-Rodriguez S, Piqueras-Sanchiz F, Faundez-Aguilera J, Bautista IJ, Barrios C, et al. Localization of Muscle Edema and Changes on Muscle Contractility After Dry Needling of Latent Trigger Points in the Gastrocnemius Muscle. Pain Med. 2019;20(7):1387-94.

24. Barbieri M. Alternative Relief. Bicycling Australia. 2011(167):126-30.

25. Barefoot JA, Bauman A. Pinpointing pain relief. Runner's World. 1998;33(9):23.

26. Bateman T. Pinning down pain. Runner's World. 2000;3(1):34-5.

27. Bauermeister W. Trigger-Diagnostik und Trigger-Stosswellen-Therapie (TST) - Praevention und Behandlung von chronisch rezidivierenden Lumbalgien und Lumboischialgien bei Muskelaufbautraining. (Poster Session) / Trigger point diagnostics and trigger point shockwave therapy (TST) - prevention and treatment of chronically recurring low back pain during strength training. Deutsche Zeitschrift fuer Sportmedizin. 2001;52(7):S100-S.

28. Beek EV. Treatment procedures in the healing of sports injuries. Coaching Science Update. 1980:60-2.

29. Beissner F. Therapeutic sensations: a new unifying concept. Evidence-Based Complementary andAlternative Medicine. 2020;2020:7630190.

30. Belko KR. Sports acupuncture. Joe Weider's Muscle & Fitness. 1993;54(10):86.

31. Benito-de-Pedro M, Becerro-de-Bengoa-Vallejo R, Losa-Iglesias ME, Rodríguez-Sanz D, López-López D, Cosín-Matamoros J, et al. Effectiveness between Dry Needling and Ischemic Compression in the Triceps Surae Latent Myofascial Trigger Points of Triathletes on Pressure Pain Threshold and Thermography: A Single Blinded Randomized Clinical Trial. J Clin Med. 2019;8(10).

32. Benito-de-Pedro M, Becerro-de-Bengoa-Vallejo R, Losa-Iglesias ME, Rodríguez-Sanz D, López-López D, Palomo-López P, et al. Effectiveness of Deep Dry Needling vs Ischemic Compression in the Latent Myofascial Trigger Points of the Shortened Triceps Surae from Triathletes on Ankle Dorsiflexion, Dynamic, and Static Plantar Pressure Distribution: A Clinical Trial. Pain Medicine. 2020;21(2):e172-e81.

33. Bennell K, Hall M, Hinman R. Osteoarthritis year in review 2015: Rehabilitation and outcomes. Osteoarthritis and Cartilage. 2016;24(1):58-70.

34. Bennett L. Acupuncture as an adjunct to standard physiotherapy in the management of adhesive capsulitis. Acupuncture in Physiotherapy. 2017;29(1):83-9.

35. Berggreen S, Wiik E, Lund H. Treatment of myofascial trigger points in female patients with chronic tension-type headache - a randomized controlled trial. Advances in Physiotherapy. 2012;14(1):10-7.

36. Bisset K. Natural sports medicine. Journal of Complementary Medicine. 2006;5(6):39-43.

37. Blizard J. Getting the needle. Athletics Weekly (0004-6671). 2015:48-9.

38. Bloomquist LE. Sports medicine in China. Alexandria, Va.;: Computer Microfilms International; 1985 1985.

39. Bodes-Pardo G, Pecos-Martin D, Gallego-Izquierdo T, Salom-Moreno J, Fernandez-de-Las-Penas C, Ortega-Santiago R. Manual treatment for cervicogenic headache and active trigger point in the sternocleidomastoid muscle: A pilot randomized clinical trial. Journal of Manipulative and Physiological Therapeutics. 2013;36(7):403-11.

40. Bonnar J. Acupuncture for sports injuries. Killshot. 1991:18-9.

41. Bopp Limoge C, Bopp J, Bopp Y. Acupuncture et performances sportives. Medecine du Sport. 1990;64(1):18-22.

42. Boylan M. Acupuncture and Stabilising Exercises Important Adjuncts in Relieving Pelvic Girdle Pain. Journal - Australian Traditional-Medicine Society. 2006;12(4):207.

43. Brage K, Ris I, Falla D, Sogaard K, Juul-Kristensen B. Pain education combined with neck- and aerobic training is more effective at relieving chronic neck pain than pain education alone A preliminary randomized controlled trial. Manual Therapy. 2015;20(5):686-93.

44. Brennan KL, Allen BC, Munoz Maldonado Y. Dry Needling Versus Cortisone Injection in the Treatment of Greater Trochanteric Pain Syndrome: A Noninferiority Randomized Clinical Trial. Journal of Orthopaedic & Sports Physical Therapy. 2017;47(4):232-9.

45. Brignol T. Place de l'acupuncture dans la prise en charge de la detresse psychologique dans des centres de soins primaires en Suede. Acupuncture et Moxibustion. 2014;13(3):202-4.

46. Brignol T, Stephan J. Acupuncture: Un facteur de risque supplementaire de sacro-iliite pyogenique chez la femme enceinte ? Acupuncture et Moxibustion. 2016;15(1):68-71.

47. Buckingham M. The runner’s dread. Athletics Weekly (0004-6671). 2012:40-2.

48. Callison M. Clinical study. Acupuncture and tibial stress syndrome (shin splints). Journal of Chinese Medicine. 2002;70:24-7.

49. Callison M. The treatment of sports-related muscle tension headaches with acupuncture and moxibustion. Journal of Chinese Medicine. 2008;87:34-8.

50. Campa-Moran I, Rey-Gudin E, FernAinverted-!ndez-Carnero J, Paris-Alemany A, Gil-Martinez A, Lerma LS, et al. Comparison of dry needling versus orthopedic manual therapy in patients with myofascial chronic neck pain: a single-blind, randomized pilot study. Pain Research and Treatment. 2015;2015:327307.

51. Cardoso R, Lumini-Oliveira J, Santos M, Ramos B, Matos L, Machado J, et al. Acupuncture can be beneficial for exercise-induced muscle soreness: A randomised controlled trial. Journal of Bodywork and Movement Therapies. 2020;24(1):8-14.

52. Cecconi A. The use of acupuncture in the emergency room. Journal of Chinese Medicine. 2011(97):60-4.

53. Cen S, Loy S, Sletten E, McLaine A. The effect of traditional Chinese Therapeutic Massage on individuals with neck pain. Clinical Acupuncture and Oriental Medicine. 2003;4(2):88-93.

54. Chan B. Acupuncture. Volleyball (10584668). 1996;7(12):68;84-68;84.

55. Chan J, Wong N, Wong D, Yeung W, Jones A. Effect of Acu-TENS on post exercise airway resistance in healthy individuals. Hong Kong Physiotherapy Journal. 2012;30(1):29-35.

56. Chan KM, Lai JS, Wong AMK, Hsu RWW, Leung PC, Liu CSC. Scientific basis of traditional medicine and practice in the management of sports injuries. ;2000 2000.

57. Chan Liat M. L' Acupuncture au service des sportifs. Paris, France: Amphora; 1981 1981.

58. Charles-Liscombe RS, Brechue WF. The effects of acupressure therapy on exercise-induced delayed onset muscle soreness and muscle. Journal of Athletic Training (National Athletic Trainers' Association). 1998;33(2):S16.

59. Cheing G, So E, Chao C. Effectiveness of electroacupuncture and interferential electrotherapy in the management of frozen shoulder. Journal of Rehabilitation Medicine. 2008;40(3):166-70.

60. Chen Z, Zhou X. The effect of acupuncture in 300 cases of acute lumbar sprain. Journal of Traditional Chinese Medicine. 1984;4(2):93-5.

61. Chiu T, Hui-Chan C, Chein G. A randomized clinical trial of TENS and exercise for patients with chronic neck pain. Clinical Rehabilitation. 2005;19(8):850-60.

62. Chou L, Hsieh Y, Chen H, Hong C, Kao M, Han T. Remote therapeutic effectiveness of acupuncture in treating myofascial trigger point of the upper trapezius muscle. American Journal of Physical Medicine and Rehabilitation. 2011;90(12):1036-49.

63. Chu J, Yuen K, Wang B, Chan R, Schwartz I, Neuhauser D. Electrical twitch-obtaining intramuscular stimulation in lower back pain: a pilot study. American Journal of Physical Medicine and Rehabilitation. 2004;83(2):104-11.

64. Chu Q, Lu L. Treatment of biceps tendonitis with electroacupuncture and local application of Yunan Bai Yao Paste. International Journal of Clinical Acupuncture. 2013;22(3):138-9.

65. Clavo J. Vital points: Chinese medical and martial arts. Black Belt. 1982;20(7):31-2.

66. Cohen M. Les indications de l'acupuncture en football. ;1989 1989.

67. Cosma D-T, Gavan N. The podiatry role in the foot care. Sports Medicine Journal / Medicina Sportivâ. 2017;13(1):2877-8.

68. Cronin S. Acupuncture for the treatment of whiplash-associated disorder. Acupuncture in Physiotherapy. 2017;29(1):39-48.

69. Cross T. Healing massage. Climbing. 2014(326):46-7.

70. Crowell MS, Brindle RA, Mason JS, Pitt W, Miller EM, Posner MA, et al. The effectiveness of battlefield acupuncture in addition to standard physical therapy treatment after shoulder surgery: a protocol for a randomized clinical trial. Trials. 2020;21(1):995.

71. Cui Y. Acupuncture of contralateral collateral points in treating soft tissue injuries: a clinical study of 1000 cases. International Journal of Clinical Acupuncture. 1994;5(4):491-5.

72. Cuignet O. Place de l'electroacupuncture dans le traitement des cephalees primaires a la lumiere de ses effets sur leur physiopathologie (2e partie). Acupuncture et Moxibustion. 2016;15(1):36-47.

73. Curatolo M. Pharmacological and Interventional Management of Pain After Whiplash Injury. Journal of Orthopaedic & Sports Physical Therapy. 2016;46(10):845-50.

74. Curley KJ, Gabbard A, Peters A, Boutagy N, Maniguet E, Zierman K, et al. The Effect Of Acupuncture Treatment On The Recovery From Delayed Onset Of Muscle Soreness(DOMS). Medicine & Science in Sports & Exercise. 2011;43:363-.

75. Cushman DM, Cummings K, Skinner L, Holman A, Haight P, Brobeck M, et al. Postrace Dry Needling Does Not Reduce Subsequent Soreness and Cramping-A Randomized Controlled Trial. Clin J Sport Med. 2021;31(3):225-31.

76. Dale R. The Chinese acupressure eye exercises. American Journal of Acupuncture. 1988;16(4):366-7.

77. Dar G, Hicks GE. The immediate effect of dry needling on multifidus muscles' function in healthy individuals. Journal of Back & Musculoskeletal Rehabilitation. 2016;29(2):273-8.

78. Davis Lake A, Myers H, Aefsky B, Butler R. I mmediate and short term effect of dry needling on triceps surae range of motion and functional movement: A randomized trial. International Journal of Sports Physical Therapy. 2018;13(2):185-95.

79. De MK, Castelein B, Coppieters I, Barbe T, Cools A, Cagnie B. Comparing trigger point dry needling and manual pressure technique for the management of myofascial neck/shoulder pain: A randomized clinical trial. Journal of Manipulative and Physiological Therapeutics. 2017;40(1):11-20.

80. Delhagen K, Legwold G. Pinpointing Pain. Runner's World. 1990;25(9):21.

81. Deng BY, Zhang JF, Luo MR, Liang L. [Effects of Ziwuliuzhu combined selection of the source point and the collateral point on athletic injuries and the state of channels]. Zhongguo Zhen Jiu. 2006;26(10):707-9.

82. Denis A. Part de l'acupuncture dans la prise en charge des acouphenes: une solution d'avenir? Acupuncture et Moxibustion. 2018;17(1):66-74.

83. Dhillon S. The acute effect of acupuncture on 20-km cycling performance. Clin J Sport Med. 2008;18(1):76-80.

84. Dilworgh PP, Baker E. Is Acupuncture or Ultrasound Therapy More Efficacious for Impingement Syndrome? Clinical Journal of Sport Medicine. 2006;16(4):376-8.

85. Dimitrios S, Malliaras P. It is time to abandon the myth that eccentric training is best practice. Biology of Exercise. 2016;12(1):15-21.

86. Diracoglu D, Vural M, Karan A, Aksoy C. Effectiveness of dry needling for the treatment of temporomandibular myofascial pain: A double-blind, randomized, placebo controlled study. Journal of Back and Musculoskeletal Rehabilitation. 2012;25(4):285-90.

87. Dlin RA, Benmair J, Hanne N. Pain relief in sports injuries - application of TENS to acupuncture points. International Journal of Sports Medicine. 1980;1(4):203-6.

88. Dlin RA, Hanne N, Benmair J. Application of TENS to acupuncture points for pain relief in sports injuries. Physics in Medicine and Biology. 1980;25(5):993-.

89. Doğan N, Şengül İ, Akçay-Yalbuzdağ Ş, Kaya T. Kinesio taping versus dry needling in the treatment of myofascial pain of the upper trapezius muscle: A randomized, single blind (evaluator), prospective study. Journal of Back & Musculoskeletal Rehabilitation. 2019;32(5):819-27.

90. Doheny K. Unconventional healers. Men's Fitness. 1994;10(2):38;40-1.

91. Dong C. High frequency manipulation at Ashi points in the treatment of soft tissue injuries. International Journal of Clinical Acupuncture. 2000;11(3):227-8.

92. Dunning J, Walsh S, Arias-BurÍA JL, Butts R, Goult C, Garcia J, et al. Spinal Manipulation and Electrical Dry Needling in Patients With Subacromial Pain Syndrome: A Multicenter Randomized Clinical Trial. Journal of Orthopaedic & Sports Physical Therapy. 2021;51(2):72-81.

93. Eftekharsadat B, Porjafar E, Eslamian F, Shakouri SK, Fadavi HR, Raeissadat SA, et al. Combination of Exercise and Acupuncture Versus Acupuncture Alone for Treatment of Myofascial Pain Syndrome: A Randomized Clinical Trial. J Acupunct Meridian Stud. 2018;11(5):315-22.

94. Elliott R, Burkett B. Massage therapy as an effective treatment for carpal tunnel syndrome. Journal of Bodywork & Movement Therapies. 2013;17(3):332-8.

95. Elwell S. Medical Acupuncture: A Western Scientific Approach. Physical Therapy in Sport. 2017;26:90-.

96. Escribano A, Berral FJ, Delgado C, Gallardo A, Berral CJ, Lancho JL. Apports au traitement de la pathologie douloureuse du denomme "coude du joueur de tennis". / Treatment of tennis elbow. Medecine du Sport. 1996;70(1):9-16.

97. EspÍ-LÓPez GV, Serra-AÑO P, Vicent-Ferrando J, SÁNchez-Moreno-Giner M, Arias-BurÍA JL, Cleland J, et al. Effectiveness of Inclusion of Dry Needling in a Multimodal Therapy Program for Patellofemoral Pain: A Randomized Parallel-Group Trial. Journal of Orthopaedic & Sports Physical Therapy. 2017;47(6):392-401.

98. Fini F, Cellerini G. Sul trattamento della pubalgia acuta, sub acuta e cronica in calciatori seeondo la tecnica della medicina tradizionale cinese. / On the treatment of acute, subacute and chronic pubic pain in soccer players using the technique of Chinese traditional medicine. Medicina dello Sport. 1981;34(1):17-26.

99. Fletcher AN, Cheah JW, Nho SJ, Mather Iii RC. Proximal Hamstring Injuries. Clinics in Sports Medicine. 2021;40(2):339-61.

100. Fousekis K. Treatment of shoulder myofascial trigger points in amateur athletes with Ergon® IASTM therapy, cupping and ischaemic pressure techniques: A randomized controlled clinical trial. Physical Therapy in Sport. 2017;28:e9-e10.

101. Fried D, Rubin M, Cassler C. Medicine of peace. European Journal of Oriental Medicine. 2017;8(6):27-33.

102. Fritsch B, Finkenzeller T, Amesberger G. Wirkung der Akupunktmassage nach Penzel (APM) bei Ruckenschmerz: Auswirkungen auf das vegetative Nervensystem und das Schmerzerleben. Zeitschrift fur Physiotherapeuten Krankengymnastik. 2013;65(11):78-82.

103. Gallaspy JB, Maneval MW. Use of finger acupressure in athletics. Athletic Training. 1986;21(1):36-9.

104. Gallucci J. Easing the Pain. Physical Therapy Products. 2012;23(6):16-9.

105. Gao A. Clinical study on the treatment of lumbar disc herniation with acupuncture and rehabilitation therapy. International Journal of Clinical Acupuncture. 2017;26(4):234-9.

106. Gao W, Liu M, Wang W. Acupuncture in treatment of peripheral nerve injury: a report of 26 cases. International Journal of Clinical Acupuncture. 1995;6(3):351-3.

107. Garlanger KL, Fredericks WH, Do A, Bauer BA, Laskowski ER. The Feasibility and Effects of Acupuncture in an Adolescent Nordic Ski Population. Pm r. 2017;9(8):795-803.

108. Gattie E, Cleland JA, Pandya J, Snodgrass S. Dry Needling Adds No Benefit to the Treatment of Neck Pain: A Sham-Controlled Randomized Clinical Trial With 1-Year Follow-up. Journal of Orthopaedic & Sports Physical Therapy. 2021;51(1):37-45.

109. Giannini G, Favoni V, Merli E, Nicodemo M, Torelli P, Matra A, et al. A Randomized Clinical Trial on Acupuncture Versus Best Medical Therapy in Episodic Migraine Prophylaxis: The ACUMIGRAN Study. Frontiers in Neurology. 2021;11.

110. Giles LG, Muller R. Chronic spinal pain: a randomized clinical trial comparing medication, acupuncture, and spinal manipulation. Spine (Phila Pa 1976). 2003;28(14):1490-502; discussion 502-3.

111. Goldman R, Stason W, Park S, Kim R, Schnyer R, Davis R, et al. Acupuncture for Treatment of Persistent Arm Pain Due to Repetitive Use: A Randomized Controlled Clinical Trial. Clinical Journal of Pain. 2008;24(3):211-8.

112. Gomez-Conesa A, Zuil-Escobar JC, Martínez-Cepa CB, Martín-Urrialde JA. Latent trigger points in lower limb muscles in sedentary and athletic subjects. A cross-sectional study on prevalence. Physiotherapy. 2016;102:e209-e10.

113. Goom T, Bradford D. Acupuncture: what's the point? Cycling Weekly. 2015(6343):42-3.

114. Gordon FK, Fahs J. Stop the soreness! Don't let a hamstring strain happen to you. Sports Medicine Update. 2002;16(2):10-3.

115. Goret O, Nguyen J, Phan-Choffrut F. Traitement des entorses aigues de la cheville par point distal unique. Acupuncture et Moxibustion. 2017;16(1):98-108.

116. Gorrell L, Beath K, Engel R. Manual and instrument applied cervical manipulation for mechanical neck pain: A randomized controlled trial. Journal of Manipulative and Physiological Therapeutics. 2016;39(5):319-29.

117. Gourion G, Nguyen A. Acupuncture et medecine sportive. Revue Francaise de Medecine Traditionnelle Chinoise. 1985(109):99-104.

118. Gouttebarge V, Veenstra E, Goedegebuure S, Frings-Dresen M, Kuijer PP. Professional football players at risk for non-acute groin injuries during the first half of the season: A prospective cohort study in The Netherlands. Journal of Back & Musculoskeletal Rehabilitation. 2018;31(1):15-21.

119. Graham J. Each runner is an individual experiment in pain. Medical experts are studying the rhyme and reason behind pain - its sources and ways to control it. Runner's World. 1981;16(4):72-5.

120. Grävare Silbernagel K, Brorsson A, Lundberg M. The Majority of Patients With Achilles Tendinopathy Recover Fully When Treated With Exercise Alone. American Journal of Sports Medicine. 2011;39(3):607-13.

121. Grieve R, Cranston A, Henderson A, John R, Malone G, Mayall C. The immediate effect of triceps surae myofascial trigger point therapy on restricted active ankle joint dorsiflexion in recreational runners: A crossover randomised controlled trial. Journal of Bodywork & Movement Therapies. 2013;17(4):453-61.

122. Grieve R, Palmer S. Physiotherapy for plantar fasciitis: a UK-wide survey of current practice. Physiotherapy (London). 2017;103(2):193-200.

123. Grillo C, Wada R, Rosario dSM. Acupuncture in the management of acute dental pain. Journal of Acupuncture and Meridian Studies. 2014;7(2):65-70.

124. Grosam B. Treating injuries with acupuncture. International Journal of Clinical Acupuncture. 2014;23(2):83.

125. Gu L. Treating soft-tissue injury of the ankle by needling points of both upper and lower limbs and simultaneous exercise. International Journal of Clinical Acupuncture. 1996;7(2):215-6.

126. Guo B, Wu Y, Wang XF, Zhao YJ, Ma HF, Jin YR. The different metabolomic profile of acupuncture stimulation in athletes after an 800-m running race and a training lesson. Medicina Dello Sport. 2018;71(3):413-28.

127. Hall M. Randomized trial of trigger point acupuncture treatment for chronic shoulder pain: A peliminary study. New Zealand Journal of Physiotherapy. 2014;42(3):177.

128. Halle R, Crowell M, Goss D. Dry needling and physical therapy versus physical therapy alone following shoulders stabilization repair: A randomized clinical trial. International Journal of Sports Physical Therapy. 2020;15(1):81-102.

129. Hamilton A. Acupuncture and recovery. Peak Performance. 2009(280):11-.

130. Hamilton A. What's the point? Peak Performance. 2010(290):1-.

131. Harding S, Swait G, Johnson IP, Cunliffe C. Utilisation of CAM by runners in the UK: A retrospective survey among non-elite marathon runners. Clinical Chiropractic. 2009;12(2):61-6.

132. Harman JC. Alternative therapies for the equine athlete. USCTA News. 1993;22(6):32-4.

133. Hattori H, Katsumi Y, Katayama K, Ikeuchi T, Ochi H, Inoue M, et al. Fact-finding in acupuncture and moxibustion treatment of baseball players in high school. ;1995 1995.

134. He L, Wen X. Treatment of tennis elbow with heat needling - a clinical summary of 58 cases. Journal of Traditional Chinese Medicine. 1988;8(2):129-30.

135. Hendrich S, Kahanov L, Eberman LE. Administration of Acupressure for Relief of Low Back Pain. International Journal of Athletic Therapy & Training. 2011;16(5):26-8.

136. Hendrich S, Kahanov L, Eberman LE. Acupressure for Tension Headache. International Journal of Athletic Therapy & Training. 2011;16(3):37-40.

137. Henry BJ, Bucher L, Mackley A, Eckman T. Journal Watch. SportEX Dynamics. 2010;26(24):4-6.

138. Horcic J. Procedury regenerace sportovcu. / Regeneration of athletes and its procedures. Trener: The Methodical Magazine. 1982;26(9):411-3.

139. Horiba H, Katayama K, Katsumi Y, Hattori H, Hanafusa F, Hirasawa Y. Investigation in the recognition and application of volleyball players to acupuncture and moxibustion treatment. ;1995 1995.

140. Horn C. Big Men, Small Needles. Joe Weider's Muscle & Fitness. 1999;60(12):161.

141. Hou C, Tsai L, Cheng K, Chung K, Hong C. Immediate effects of various physical therapeutic modalities on cervical myofascial pain and trigger-point sensitivity. Archives of Physical Medicine and Rehabilitation. 2002;83(10):1406-14.

142. Hsieh Y, Kao M, Kuan T, Chen S, Chen J, Hong C. Dry Needling to a Key Myofascial Trigger Point May Reduce the Irritability of Satellite MTrPs. American Journal of Physical Medicine and Rehabilitation. 2007;86(5):397-403.

143. Hu B, Zhang HX. A survey of treatment to hyperplastic ostearthritis with acupuncture and moxibustion. Journal of Hubei Sports Science. 1998;17(2):43-5.

144. Hu LC. Selection of points in acupuncture treatment of sport injury. Journal of Hubei Sports Science. 2000;19(1):46-8.

145. Huang W, Li S, Fan Z, Wu S, Huang Z, Cheng L. A clinical study of 30 cases of lumbodorsal myofascial pain syndrome treated by plucking on trigger points. International Journal of Clinical Acupuncture. 2016;25(4):258-60.

146. Huey L. Acupressure and body balancing. Racquetball Illustrated. 1981;4(11):41-3.

147. Hufton E. Targeted Relief. Runner's World (Australia & New Zealand Edition). 2008;11(1):32-3.

148. Huguenin L, Brukner P, McCrory P, Smith P, Wajswelner H, Bennell K. The effect of dry needling of gluteal muscles on straight leg raise-a randomised placebo controlled double blind trial. (Abstract). Journal of Science & Medicine in Sport. 2003;6:110-.

149. Huguenin L, Brukner PD, McCrory P, Smith P, Wajswelner H, Bennell K. Effect of dry needling of gluteal muscles on straight leg raise: a randomised, placebo controlled, double blind trial. British Journal of Sports Medicine. 2005;39(2):84-90.

150. Ingber R. Myofascial pain in lumbar dysfunction. Physical Medicine and Rehabilitation: State of the Art Reviews. 1999;13(3):473-98.

151. Itch K, Salto S, Sahara S, Naitoh Y, Imai K, Kitakoji H. Randomized trial of trigger point acupuncture treatment for chronic shoulder pain: A preliminary study. Journal of Acupuncture and Meridian Studies. 2014;7(2):59-64.

152. Jack M, Tierney R, Mansell J, Russ A. Dry Needling Versus Sham Needling for Myofascial Pain Syndrome: A Critically Appraised Topic. International Journal of Athletic Therapy & Training. 2020;25(6):289-93.

153. Jagadish A, Nandyala SV, Marquez-Lara A, Singh K, Lee Y-P. Spinal Interventions-The Role in the Athlete. Operative Techniques in Sports Medicine. 2013;21(3):185-90.

154. James SLJ, Ali K, Pocock C, Robertson C, Walter J, Bell J, et al. Ultrasound guided dry needling and autologous blood injection for patellar tendinosis. British Journal of Sports Medicine. 2007;41(8):518-22.

155. Javens JA. Effect of acupuncture TENS on second degree ankle sprains. Eugene, Ore.;: Microform Publications, College of Human Development and Performance, University of Oregon; 1992 1992.

156. Jeffs P. Acupuncture for chronic patellar tendinopathy. Journal - Acupuncture Association of Chartered Physiotherapists. 2013;2013(3):79-86.

157. Jermyn E. Point of attack. Training & Conditioning. 2014;24(8):14-9.

158. Ji X. Injury of sacroiliac joint. International Journal of Clinical Acupuncture. 2000;11(2):121-3.

159. Jiang Y, Zou J. Analysis of the TCM theory of traditional Chinese health exercise. Journal of Sport & Health Science. 2013;2(4):204-8.

160. Jiang Z, Li C. Acupuncture and exercise in treatment of periarthritis of shoulder. International Journal of Clinical Acupuncture. 2000;11(3):233-6.

161. Jin Z. 10 cases of oculomotor nerve injuries treated with acupuncture therapy. International Journal of Clinical Acupuncture. 1990;1(2):205-7.

162. Johansson KM, Adolfsson LE, Foldevi MOM. Effects of Acupuncture Versus Ultrasound in Patients With Impingement Syndrome: Randomized Clinical Trial. Physical Therapy. 2005;85(6):490-501.

163. Jones H. Any alternatives? From acupuncture to yoga, the list of complementary therapies grows ever longer. So what can they do for you? Runner's World (UK). 1995;3(12):46-8;50-1.

164. Jull G, Kenardy J, Hendrikz J, Cohen M, Sterling M. Management of acute whiplash: A randomized controlled trial of multidisciplinary stratified treatments. Pain. 2013;154(9):1798-806.

165. Kamien M. A rational management of tennis elbow. Sports Medicine. 1990;9(3):173-91.

166. Kaptchuk TJ, Stason WB, Davis RB, Legedza ATR, Schnyer RN, Kerr CE, et al. Sham device versus inert pill: randomised controlled trial of two placebo treatments. Bmj-British Medical Journal. 2006;332(7538):391-4.

167. Karagounis P, Tsironi M, Prionas G, Tsiganos G, Baltopoulos P. Treatment of plantar fasciitis in recreational athletes: two different therapeutic protocols. Foot Ankle Spec. 2011;4(4):226-34.

168. Kargela M, Silvemail J, Blickenstaff C, Kruger E, Brennan KL. Comparing dry needling to corticosteroid injection for greater trochanteric pain syndrome. Journal of Orthopaedic & Sports Physical Therapy. 2017;47(8):583-5.

169. Kastner M. The treatment of tendon pain with traditional Chinese medicine. Journal of Chinese Medicine. 2014;106:5-11.

170. Kastner R. Electro acupuncture and its uses. Leistungssport. 1974;4(3):221-6.

171. Katayama K. Acupuncture approach to pain. Japanese Journal of Clinical Sports Medicine. 2005;13(3):301-11.

172. Katayama K, Ochi H, Ikeuchi T, Matsumoto T, Tanaka C, Katsumi Y, et al. Effect of acupuncture treatment on delayed onset muscular soreness studied by magnetic resonance method. ;1995 1995.

173. Kennedy Gordon F. Acupuncture for the athlete: East meets West with use of an ancient Chinese practice. Sports Medicine Update. 2000;15(1):26-8.

174. Kent JB, Tanabe KO, Muthusubramanian A, Statuta SM, MacKnight JM. Complementary and Alternative Medicine Prescribing Practices Among Sports Medicine Providers. Alternative Therapies in Health and Medicine. 2020;26(5):28-32.

175. Khanittanuphong P, Upho P. Day of peak pain reduction by a single session of dry needling in the upper trapezius myofascial trigger points: A 14 daily follow-up study. Journal of Bodywork and Movement Therapies. 2020;24(4):7-12.

176. Kim K, Jeong Y, Youn Y, Choi J, Kim J, Chung W, et al. Nonoperative Korean medicine combination therapy for lumbar spinal stenosis: a retrospective case-series study. Evidence-Based Complementary andAlternative Medicine. 2015;2015:263898.

177. Kim S, Kim B, Kim J. The use of complementary and alternative medicine among Korean young adult members of fitness centers. Evidence-Based Complementary andAlternative Medicine. 2019;2019:7648237.

178. Kishmishian B, Richards J, Selfe J. A randomised feasibility study using an acupuncture protocol to the Achilles tendon in Achilles tendinopathy. Physiotherapy Practice and Research. 2019;40(1):59-67.

179. Kizhakkeveettil A, Rose K, Kadar G, Hurwitz E. Integrative acupuncture and spinal manipulative therapy versus either alone for low back pain: A randomized controlled trial feasibility study. Journal of Manipulative and Physiological Therapeutics. 2017;40(3):201-13.

180. Koelblinger G, Tanaka O, Mukaino Y. The meridiantest - a new method of defining effective meridians for the treatment of painful disorders of the shoulder by acupuncture. ;1995 1995.

181. Kong F. Auricular needling in the treatment of soft tissue injury. International Journal of Clinical Acupuncture. 1991;2(2):205-6.

182. Koppenhaver S, Walker M, Su J, McGowen J, Umlauf L, Harris K, et al. Changes in lumbar multifidus muscle function and nociceptive sensitivity in low back pain patient responders versus non-responders after dry needling treatment. Manual Therapy. 2015;20(6):769-76.

183. Koppenhaver SL, Walker MJ, Smith RW, Booker JM, Walkup ID, Su J, et al. Baseline Examination Factors Associated With Clinical Improvement After Dry Needling in Individuals With Low Back Pain. Journal of Orthopaedic & Sports Physical Therapy. 2015;45(8):604-12.

184. Koszalinski A, Flynn T, Hellman M, Cleland JA. Trigger point dry needling, manual therapy and exercise versus manual therapy and exercise for the management of Achilles tendinopathy: a feasibility study. Journal of Manual & Manipulative Therapy (Taylor & Francis Ltd). 2020;28(4):212-21.

185. Krabak B, Borg-Stein J, Oas J. Chronic cervical myofascial pain syndrome: improvement in dizziness and pain with a multidisciplinary rehabilitation program. A pilot study. Journal of Back and Musculoskeletal Rehabilitation. 2000;15(2):83-7.

186. Kresge C, Karcy B, Ozeri M. Sports massage series, volume 8 : An intro to acupressure and sports massage. (New York);: Larador Productions and Bob Karcy Productions; 1988 1988.

187. Kwak HY, Kim JI, Park JM, Lee SH, Yu HS, Lee JD, et al. Acupuncture for Whiplash-associated disorder: A randomized, waiting-list controlled, pilot trial. European Journal of Integrative Medicine. 2012;4(2):E151-E8.

188. Larimore WL. Sprains, strains, trigger points, and soft tissue injuries. ;1994 1994.

189. Lebert R. Myofascial Trigger Points. Massage Magazine. 2016(247):52-7.

190. Lee J, Eun S, Kim J, Lee JH, Park K. Differential Influence of Acupuncture Somatosensory and Cognitive/Affective Components on Functional Brain Connectivity and Pain Reduction During Low Back Pain State. Frontiers in Neuroscience. 2019;13.

191. Lee YS, Park DS, Oh JK, Kim SY. Prediction model for utilization of complementary and alternative medicine for sports injuries among Korean elite collegiate athletes. Integr Med Res. 2020;9(2):100401.

192. Lehmann T, Russell D, Spratt K, Colby H, Liu Y, Fairchild M, et al. Efficacy of electroacupuncture and TENS in the rehabilitation of chronic low back pain patients. Pain. 1986;27(3):277-90.

193. Lehn C. Massage. 1990.

194. Lei Luo. Curative Effect Observation of the Treatment of Shoulder Impingement Syndrome by Zheng's Old Wound Medicine with Acupuncture. Journal of Chengdu Sport University. 2014;40(5):72-4.

195. Lenkowitz E. Where pricka aren’t bad. Men's Fitness. 2008;24(2):36-.

196. Leonard J. Nick Thierry. Swimnews. 2008(300):4-.

197. Levy LS, Bernier JN, Perrin DH. Lab 6: myofascial release, myofascial trigger points, and muscle energy. ;2001 2001.

198. Li F. Treatment and verification of scapulohumeral periarthritis by acupuncture. Chinese Journal of Sports Medicine. 1997;16(3):176-8.

199. Li G, Shi H, Duan L. An epidemiological investigation of sports trauma in swimming. Chinese Journal of Sports Medicine. 1998;17(2):129-34.

200. Li X, Li X. Clinical application of keeping-fit acupuncture. International Journal of Clinical Acupuncture. 1990;1(3):265-70.

201. Li Y. Tenosynovitis of table tennis players and its treatment. Sports Science/Tiyu Kexue. 1996;16(5):67-70.

202. Lin J, Yang S. Effects of acupuncture on exercise-induced muscle soreness and serum creatine kinase activity. American Journal of Chinese Medicine. 1999;27(3):299-305.

203. Lin ZP, Chen YH, Chia F, Wu HJ, Lan LW, Lin JG. Episodes of injuries and frequent usage of traditional Chinese medicine for Taiwanese elite wrestling athletes. Am J Chin Med. 2011;39(2):233-41.

204. Lipner M. Different strokes. Women's Sports & Fitness. 1993;15(4):31.

205. Liu M. Clinical observations on 72 cases of periarthritis of the shoulder treated by needle-warming moxibustion combined with massage. International Journal of Clinical Acupuncture. 2015;24(1):38-40.

206. Liu YS, Gadau M, Zhang GX, Liu H, Wang FC, Zaslawski C, et al. Acupuncture treatment of lateral elbow pain: A nonrandomized pilot study. Evidence-Based Complementary andAlternative Medicine. 2016;2016:8182071.

207. Llamas-Ramos R, Pecos-Martin D, Gallego-Izquierdo T, Llamas-Ramos I, Plaza-Manzano G, Ortega-Santiago R, et al. Comparison of the Short-Term Outcomes Between Trigger Point Dry Needling and Trigger Point Manual Therapy for the Management of Chronic Mechanical Neck Pain: A Randomized Clinical Trial. Journal of Orthopaedic & Sports Physical Therapy. 2014;44(11):852-61.

208. Loesel H. Valuable knowledge from Korea: Oriental medicine. / Wissenswertes aus Korea: Orientalische Medizin. UIT Shooting Sport Journal. 1987(3):12-21.

209. Loizidis T, Nikodelis T, Bakas E, Kollias I. The effects of dry needling on pain relief and functional balance in patients with sub-chronic low back pain. Journal of Back & Musculoskeletal Rehabilitation. 2020;33(6):953-9.

210. Long BC. Repeated Electrical Point Stimulation (EPS) Treatments for Myofascial Trigger Points in the Upper Trapezius Muscle. International Journal of Athletic Therapy & Training. 2021;26(1):36-41.

211. Longworth W, McCarthy P. Acupuncture treatment of lumbar disc related disorders. Acupuncture in Medicine. 1998;16(1):18-31.

212. Lu D, Duan C, Zhang J, Fan J, Tang X. Effect of acupuncture on ultrastructural alteration in skeletal muscle after strenous exercise. Med Sport Sci. 1988;28:China's Sports Medicine 90-3.

213. Lu M. Acupuncture plus cupping in the treatment of acute rhomboid muscle injury: a report of 143 cases. International Journal of Clinical Acupuncture. 1992;3(4):385-7.

214. Lucas KR, Polus BI, Rich PA. Latent myofascial trigger points: their effect on the temporal muscle recruitment pattern during scapular plane elevation. Sport Health. 2003;21(2):13-4.

215. Luo P, Lin H. Curative efficacy of myofascial pain syndrome (MPS) treated by comprehensive treatment focusing on acupuncture stretching. International Journal of Clinical Acupuncture. 2017;26(2):85-90.

216. Ma J. Periomarthritis treated with pain point pressure in combination with local exercises. J Trad Chin Med. 1995;15(4):289.

217. Macznik A, Schneiders A, Sullivan J, Athens J. Management of pain associated with acute sports injuries—Is acupressure a way to go? Journal of Science & Medicine in Sport. 2013;16:e8-e.

218. Mącznik AK, Schneiders AG, Athens J, Sullivan SJ. Does Acupressure Hit the Mark? A Three-Arm Randomized Placebo-Controlled Trial of Acupressure for Pain and Anxiety Relief in Athletes With Acute Musculoskeletal Sports Injuries. Clinical Journal of Sport Medicine. 2017;27(4):338-43.

219. Mahé L. Getting the needle. Cycling Weekly. 2014(6320):71-.

220. Mallon B. Don't overlook `alternative' choices. Golf Digest. 1995;46(7):12-C.

221. Manca A, Dumville J, Torgerson D, Klaber MJ, Mooney M, Jackson D, et al. Randomized trial of two physiotherapy interventions for primary care back and neck pain patients: cost effectiveness analysis. Rheumatology. 2007;46(9):1495-501.

222. Manietta K. Putting your best foot forward. Run for Your Life: R4YL. 2010(33):56-7.

223. Martin M. Acupressure technique offers female athletes relief from menstrual cramps. First Aider. 1982;52(3):10-.

224. Mason JS, Crowell M, Dolbeer J, Morris J, Terry A, Koppenhaver S, et al. The effectiveness of dry needling and streching VS. streching alone on hamstring flexibility in patients with knee pain: A randoized controlled trial. International Journal of Sports Physical Therapy. 2016;11(5):672-83.

225. McCray RE, Patton NJ. Pain relief at trigger points: a comparison of moist head and shortwave diathermy. Journal of Orthopaedic & Sports Physical Therapy. 1984;5(4):175-8.

226. McCray S. Understanding the Perceptions of Dry Needling in NCAA Division I Athletes. 2019.

227. McMakin C. Microcurrent therapy: a novel treatment method for chronic low back myofascial pain. Journal of Bodywork and Movement Therapies. 2004;8(2):143-53.

228. Meguriya S, Kobayashi T, Miyamoto T, Yamaguchi S, Fukubayashi T. Acupuncture therapy for ankle sprains in athletes. ;1995 1995.

229. Meisingset I, Stensdotter A, Woodhouse A, Vasseljen O. Neck motion, motor control, pain and disability: A longitudinal study of associations in neck pain patients in physiotherapy treatment. Manual Therapy. 2016;22:94-100.

230. Mejuto-VÁZquez MJ, Salom-Moreno J, Ortega-Santiago R, Truyols-DomÍNguez S, FernÁNdez-De-Las-PeÑAs C. Short-Term Changes in Neck Pain, Widespread Pressure Pain Sensitivity, and Cervical Range of Motion After the Application of Trigger Point Dry Needling in Patients With Acute Mechanical Neck Pain: A Randomized Clinical Trial. Journal of Orthopaedic & Sports Physical Therapy. 2014;44(4):252-60.

231. Mellion MB. Neck and back pain in bicycling. Clinics in Sports Medicine. 1994;13(1):137-64.

232. Michaelson P, Sjolander P, Johansson H. Factors predicting pain reduction in chronic back and neck pain after multimodal treatment. Clinical Journal of Pain. 2004;20(6):447-54.

233. Middleton C. Needles and wins: the world of medicine has some unorthodox ways of helping sports men. (acupuncture). Sport & Leisure. 1981;22(2):38-9.

234. Mitchell IC. Remedial gymnastics and acupuncture - a combined approach. Journal of the Society of Remedial Gymnastics & Recreational Therapy. 1984(113):14-7.

235. Mitterbauer G. Akupunktur im Dienste des Sports Ein Diskussionsbeitrag anlasslich einer interdisziplinar durchgefuhrten Fallstudie. (L' acupuncture au service du sport A propos d' une etude de cas traite de maniere interdisciplinaire). Leibesubungen - Leibeserziehung. 1982(3):67-75.

236. Miyamoto T, Kobayashi T, Meguriya S, Yamaguchi S, Fukubayashi T, Hayashi K. The actual condition of acupuncture for athletes enterd(sic) the National Athletic Meet in Ibaraki Prefecture. ;1995 1995.

237. Molsberger A, Hille E. The analgesic effect of acupuncture in chronic tennis elbow pain. British Journal of Rheumatology. 1994;33(12):1162-5.

238. Molsberger AF, Mau J, Pawelec DB, Winkler J. Does acupuncture improve the orthopedic management of chronic low back pain - a randomized, blinded, controlled trial with 3 months follow up. Pain. 2002;99(3):579-87.

239. Monkerud D. Put your health in your hands. Runner's World. 1976;11(12):32-7.

240. Monkerud D. Putting your finger on the source of pain. The solution to many of the pains running cause may already be at your fingertips. Runner's World. 1979;14(8):59-61.

241. Morgan BC, Deyle GD, Petersen EJ, Allen CS, Koppenhaver SL. Dry needling in the management of patients meeting clinical diagnostic criteria for subcromial pain syndrome: A case series. International Journal of Sports Physical Therapy. 2019;14(4):637-54.

242. Morison A, Balliett M, Merkle P, Burke J. Prevalence of chronic musculoskeletal spinal injuries in the sport of artificial track luge. Science & Sports. 2016;31(5):290-6.

243. Muijs K, Giesen H. Acupuncture for canoeing. Leistungssport. 1974;4(3):218-21.

244. Mullins JF, Nitz AJ, Hoch MC. The Effect of Dry Needling on Patient-Reported Outcomes in Individuals With Chronic Ankle Instability: A Critically Appraised Topic. International Journal of Athletic Therapy & Training. 2019;24(5):186-92.

245. Murphy P. Acupressure and volleyball. Volleyball Technical Journal. 1982;6(3):65-6.

246. Mustafin SM. (Stimulations douloureuses: limites des algozones cutanees dans le traitement des traumatismes en sport). Teoria i Praktika Fiziceskoj Kul'tury. 1987(7):54-5.

247. Nayak S, Shiflett S, Schoenberger N, Agostinelli S, Kirshblum S, Averill A, et al. Is acupuncture effective in treating chronic pain after spinal cord injury? Archives of Physical Medicine and Rehabilitation. 2001;82(11):1578-86.

248. Nichols AW, Harrigan R. Complementary and Alternative Medicine Usage by Intercollegiate Athletes. (Poster Session). Clinical Journal of Sport Medicine. 2005;15(5):390-.

249. Nichols AW, Harrigan R. Complementary and Alternative Medicine Usage by Intercollegiate Athletes. Clinical Journal of Sport Medicine. 2006;16(3):232-7.

250. Nikituk BA, Samoilov DNG. Adaptation of skeletal muscles to increased physical loads through laseropuncture. Fitness & Sports Review International. 1993;28(3):73-4.

251. Noran S. Acupuncture in the service of sport. / L'acupuncture au service du sport. Sports in Romania/Sport en Roumanie. 1985(1):24-5;44-5.

252. Noxon B. Natural Cures. Runner's World. 2008;43(7):43-4.

253. Nugent-Head A. Ashi points in-clinical practice. Journal of Chinese Medicine. 2013;101:5-12.

254. Nyland J, Nolan MF. Therapeutic modality: rehabilitation of the injured athlete. Clinics in Sports Medicine. 2004;23(2):299-313.

255. O'Sullivan K, O'Sullivan P. The ineffectiveness of paracetamol for spinal pain provides opportunities to better manage low back pain. British Journal of Sports Medicine. 2016;50(4):197-8.

256. Okuma Y, Miyazaki S, Yoshida N, Mukaino Y, Hisajima T, Takahashi H. Complementary and Alternative Medicine Usage by Intercollegiate Athletes in Japan. Japanese Journal of Clinical Sports Medicine. 2012;20(2):351-9.

257. Okuma Y, Mukaino Y. The effect of continuing acupuncture treatment based on the M-Test for collegiate rugby players. Japanese Journal of Clinical Sports Medicine. 2010;18(2):264-73.

258. Okuma Y, Mukaino Y. Effects on reaction time in sprinters of press tack acupuncture treatment based on evaluation of movement-induced somatic response. Japanese Journal of Clinical Sports Medicine. 2011;19(2):250-7.

259. Oliveira VC, Henschke N. Multimodal physiotherapy is effective for anterior knee pain relief. British Journal of Sports Medicine. 2013;47(4):245-6.

260. Orlando G. Agopuntura nel volley. / Acupuncture in volleyball. Pallavolo. 1981;17(2):54-5.

261. Ortega-Cebrian S, Luchini N, Whiteley R. Dry needling: Effects on activation and passive mechanical properties of the quadriceps, pain and range during late stage rehabilitation of ACL reconstructed patients. Phys Ther Sport. 2016;21:57-62.

262. Pach D, Yang-Strobel X, Ludtke R, Roll S, Icke K, Brinkhaus B, et al. Standardized versus Individualized Acupuncture for Chronic Low Back Pain: A Randomized Controlled Trial. Evidence-Based Complementary and Alternative Medicine. 2013;2013.

263. Pan H, Pan H. Impact of Acupuncture Applied to Sanyinjiao on the Movement Ability of Female Athletes. International Journal of Clinical Acupuncture. 2007;16(3):157-61.

264. Paoloni M, Tavernese E, Cacchio A, Tattoli M, Melis L, Ronconi R, et al. Patient-oriented rehabilitation in the management of chronic mechanical neck pain: A randomized controlled trial. European Journal of Physical and Rehabilitation Medicine. 2013;49(3):273-81.

265. Park J, Kim SY, Eggett DL, Chinn DH, Squires A, Smith SC, et al. Cryotherapy and Acupuncture did not Immediately Change Quadriceps Motoneuron Pool Excitability in Induced Knee Pain. Medicine & Science in Sports & Exercise. 2011;43:765-.

266. Pavkovich R. Effectiveness of dry needling, streching, and strenghthening to reduce pain and improve function in subjects with chronic lateral hip and thigh pain: A retrospective case series. International Journal of Sports Physical Therapy. 2015;10(4):540-51.

267. Pearson E. Combined manual medicine and acupuncture in neck injury. J Manual Med. 1990;5(1):19-20.

268. Pecos-Martin D, Montanez-Aguilera F, Gallego-Izquierdo T, Urraca-Gesto A, Gomez-Conesa A, Romero-Franco N, et al. Effectiveness of dry needling on the lower trapezius in patients with mechanical neck pain: A randomized controlled trial. Archives of Physical Medicine and Rehabilitation. 2015;96(5):775-81.

269. Pelham TW, Holt LE, Stalker R. Acupuncture in human performance. Journal of Strength & Conditioning Research (Allen Press Publishing Services Inc). 2001;15(2):266-71.

270. Peppaid A, Riegler HF. Trigger-point therapy for myofascial pain. Physician & Sportsmedicine. 1981;9(6):161; 4.

271. PÉRez-Palomares S, Gaspar-Calvo E, PÉRez-Benito M, LÓPez-LapeÑA E, De La Torre-Beldarrain ML, MagallÓN-Botaya R, et al. Contribution of Dry Needling to Individualized Physical Therapy Treatment of Shoulder Pain: A Randomized Clinical Trial. Journal of Orthopaedic & Sports Physical Therapy. 2017;47(1):11-20.

272. Perle SM. Myofascial trigger points. Chiropractic Sports Medicine. 1995;9(3):106-8.

273. Petal M. Acupuncture? Athletes say yes. Physician & Sportsmedicine. 1978;6(9):22-.

274. Pfab F. Active Voice: Dry Needling -- An Option to Improve Muscle Strength and Flexibility and Prevent Muscular Injuries? Sports Medicine Bulletin. 2017:2-.

275. Pham C, Commandre F, Riaud G, Ferrer JR, Foix XP, Ramirez C. Place de l' acupuncture comme therapie anti-inflammatoire en medecinedu sport. Lyon Mediterranee Medical Medecine du Sud-Est. 1988;24:11695-8.

276. Philippides D. L' Acupuncture en milieu sportif: (etude clinique de son application en traumatologie sportive): a propos de 111 cas traites a l' Institutnational des sports. France1980 1980.

277. Piers A. Acupuncture for sports. A research project. Br J Acupunct. 1986;9(2):29-32.

278. Ping LUO, Hong-Sheng LIN. Curative Efficacy of Myofascial Pain Syndrome(MPS) bya Comphensive Treatment Focusing on Acupuncture Stretching. Journal of Chengdu Sport University. 2013;39(8):75-9.

279. Piras S, Pizzolato F, Guidol A, Zotti A, Furlanetto G, Agostini M. Laser terapia ed aggopuntura nella microtraumatologia da sovraccariconel giovane atleta (Therapie au laser et accupuncture dans la microtraumatologie due a la surcharge chez le jeune athlete). Medicina dello Sport. 1988;41(4):241-5.

280. Pugazhendi S, Rajamani P, Daniel AS, Pugazhendi K. Non-invasive Complementary Therapies in Managing Musculoskeletal Pains and in Preventing Surgery. Int J Ther Massage Bodywork. 2020;13(2):9-18.

281. Qasrawi H, Assi S, Ghanim N, Zyoud SH, Al-Jabi SW. A Descriptive Study of Pain Relief Practices Among Student-Athletes in Palestine: Focus on Non-Steroidal Anti-Inflammatory Drugs, and Complementary Medicine and Alternative Medicine Use. Journal of Community Health.

282. Rajamani P, Shewade HD, Kundu D, Sekaran KK, Amalan SD, Pugazhendi S, et al. Completion Rates and Clinical Changes of Patients Seeking Non-Invasive Treatment for Low Back Pain in 13 Centres of a Sports Medicine Institute in India. Complement Med Res. 2020;27(2):89-96.

283. Ransone JW, Schmidt J, Crawford SK, Walker J. Effect of manual compressive therapy on latent myofascial trigger point pressure pain thresholds. Journal of Bodywork & Movement Therapies. 2019;23(4):792-8.

284. Ranucci M, Ravanelli A. Il blocco dei punti trigger nella piccola traumatologia sportiva (Le blocage des points trigger en traumatologie sportive legere). Medicina dello Sport. 1984(1):25-8.

285. Razavi M, Jansen G. Effects of acupuncture and placebo TENS in addition to exercise in treatment of rotator cuff tendinitis. Clinical Rehabilitation. 2004;18(8):872-8.

286. Reaves W. Acupuncture and the Athlete. ACSM Fit Society Page. 2008:4-.

287. Reaves W. Plantar fasciitis: The acupuncture treatment of heel pain. Journal of Chinese Medicine. 2011;96:22-5.

288. Rempp C. Place de l'acupuncture en medecine du sport. Lyon Mediterranee Medical Medecine du Sud-Est. 1980;16:2975-8.

289. Rezvani M, Yaraghi A, Mohseni M, Fathimoghadam F. Efficacy of yamamoto new scalp acupuncture versus traditional Chinese acupuncture for migraine treatment. Journal of Alternative & Complementary Medicine - New York. 2014;20(5):371-4.

290. Rogers AE, Baker J, Beutler A, Witkop C, Leggit JC. Injury and Illness Surveillance During the 2016 Department of Defense Warrior Games: Review of Methods and Results. Mil Med. 2019;184(11):e616-e21.

291. Rossi A, Blaustein S, Brown J, Dieffenderfer K, Ervin E, Griffin S, et al. Spinal amd peripheral dry needling versus peripheral dry needling alone among individuals with a history of lateral ankle sprain: A randomized controlled trial. International Journal of Sports Physical Therapy. 2017;12(7):1034-47.

292. Rubi-Klein K, Kucera-Sliutz E, Nissel H, Bijak M, Stockenhuber D, Fink M, et al. Is acupuncture in addition to conventional medicine effective as pain treatment for endometriosis? A randomised controlled cross-over trial. European Journal of Obstetrics & Gynecology and Reproductive Biology. 2010;153(1):90-3.

293. Rueda GJ, Vas J, Lopez D. Acupuncture treatment of shoulder impingement syndrome: A randomized controlled trial. Complementary Therapies in Medicine. 2016;25:92-7.

294. Russell B. A two-year study: acupuncture and the treatment of dance injuries. (Poster Session). Journal of Dance Medicine & Science. 2003;7(2):66-.

295. Salehi S, Hesami O, Esfehani MP, Khosravi S, Rashed A, Haghighatzadeh M, et al. The Effectiveness of Exercise Therapy and Dry Needling on Wrist Range of Motion, Pinch and Grip Force in Carpal Tunnel Syndrome: A Randomized Clinical Trial. Asian Journal of Sports Medicine. 2019;10(4):1-9.

296. Salom-Moreno J, Ayuso-Casado B, Tamaral-Costa B, Sanchez-Mila Z, Fernandez-de-las-Penas C, Alburquerque-Sendin F. Trigger Point Dry Needling and Proprioceptive Exercises for the Management of Chronic Ankle Instability: A Randomized Clinical Trial. Evidence-Based Complementary and Alternative Medicine. 2015;2015.

297. Schilling G. Methodes psychoregulatrices et le sport helvetique - alibi ou dernier recours? Compte rendu des experiences faites dans ce domaine. Jeunesse & Sport. 1979;5:110-3.

298. Schilling G. Les methodes psycho-regulatrices et le sport helvetique. Alibi ou dernier recours? Compte rendu des essais d'application. ;1982 1982.

299. Schwanitz R. Acupuncture and related methods applied in sports medicine: Exemplified by rupture of a muscle fiber. Med Acupunct. 2007;19(2):105-8.

300. Schwarcz AE. Effects of transcutaneous electrical nerve stimulation to selected acupuncture points for treatment of second-degree inversion ankle sprains. Eugene, Ore.;: Microform Publications, College of Human Development and Performance, University of Oregon; 1987 1987.

301. Sheehan G. Medical advice: fever; hypoglycemia; calf strain; Osgood-Schlatter's; swollen glands; nose breathing; quadriceps; acupressure; depression. Runner's World. 1977;12(6):21-3.

302. Shi Y, Huang M, Luo H. 30 cases of cervico-shoulder myofascitis treated by meridian scraping therapy combined with pricking and cupping therapy. International Journal of Clinical Acupuncture. 2016;25(4):278-80.

303. Shirzadi Z, Rojhani-Shirazi Z, Hemmati L. A Comparison Between the Effects of Scapulothoracic Mobilization Plus Physical Therapy With Physical Therapy Alone in Patients With Mechanical Neck Pain: A Randomized Clinical Trial. Journal of Chiropractic Medicine. 2018;17(4):237-43.

304. Silvério Lopes S, da Mota MPG. Efeito da acupuntura na resistência física após exercícios repetitivos de tornozelo - estudo experimental. / Effects of acupuncture in physical strength after repetitive ankle exercises - experimental study. Revista Brasileira de Ciência e Movimento: RBCM. 2018;26(1):13-21.

305. Skatteboe S, Roe C, Fagerland M, Granan L. Expectations for treatment outcomes in neck/back patients regarding improvements in pain and function: A cross-sectional pilot study. European Journal of Physical and Rehabilitation Medicine. 2014;50(6):649-56.

306. Skatteboe S, Roe C, Fagerland M, Granan L. The influence of expectations on improvements in pain and function in patients with neck/back/shoulder complaints: A cohort study. European Journal of Physical and Rehabilitation Medicine. 2017;53(6):936-43.

307. Skorczyk WW. Acpuncture in sports medicine. Sportarzt und Sportmedizin. 1977;28(8):252-4.

308. Siqueira APR, Beraldo LM, Krueger E, Ulbricht L. Reduction in knee oain symptoms in athletes using an acupuncture protocol. Acta Ortop Bras. 2018;26(6):418-22

309. Smith JM, Sullivan SJ, Baxter GD. A descriptive study of the practice patterns of massage new zealand massage therapists. Int J Ther Massage Bodywork. 2011;4(1):18-27.

310. Solomons L, Lee JJY, Bruce M, White LD, Scott A. Intramuscular stimulation vs sham needling for the treatment of chronic midportion Achilles tendinopathy: A randomized controlled clinical trial. Plos One. 2020;15(9).

311. Song Z. Treatment of 1000 cases of lumbar soft tissue injury with acupuncture plus exercise. J Trad Chin Med. 1993;13(1):19-21.

312. Song Z. Exercise-associated acupuncture therapy (part 2). International Journal of Clinical Acupuncture. 2000;11(4):315-7.

313. Southall AJ. Healing through trigger point therapy:A guide to fibromyalgia,myofascial pain, and dysfunction. Journal of Orthopaedic & Sports Physical Therapy. 2014;44(7):544-5.

314. Srbely J, Dickey J, Lee D, Lowerison M. Dry needle stimulation of myofascial trigger points evokes segmental anti-nociceptive effects. Journal of Rehabilitation Medicine. 2010;42(5):463-8.

315. Stacey R. Acupuncture and alternative therapies in premiership football clubs. Acupuncture in Medicine. 1999;17(1):62-3.

316. Stieven FF, Ferreira GE, Wiebusch M, De Ara ÚJo FX, Telles Da Rosa LH, Faria Silva M. Dry Needling Combined With Guideline- Based Physical Therapy Provides No Added Benefit in the Management of Chronic Neck Pain: A Randomized Controlled Trial. Journal of Orthopaedic & Sports Physical Therapy. 2020;50(8):447-54.

316. Stone JA. Prevention and rehabilitation. Myofascial techniques: trigger-point therapy. Athletic Therapy Today. 2000;5(3):54-5.

317. Sun W, Li J. Treatment for injury of superior clunial nerves by triple puncture needling with massage. Journal of Traditional Chinese Medicine. 2002;22(1):24-5.

318. Sutlive TG, Moore JH, Golden A, King K, Morris WB, Morrison JE, et al. Short-term effects of Trigger point dry needling on pain and disability in subjects with patellofemoral pain syndrome. International Journal of Sports Physical Therapy. 2018;13(3):462-73.

319. Tang WJ, Jiang CG, Chen LR, Pang Y, Li J, Huang Y. [Effects of acupuncture-moxibustion intervention on proprioception in athletes with lateral collateral ligament injury of ankle joint]. Zhen Ci Yan Jiu. 2013;38(4):314-8.

320. Tas-Cebe G, Cummings C. A single-blinded controlled trial comparing corticosteroid injection, acupuncture and home-based exercises for the treatment of primary contracted (frozen) shoulder. Journal - Acupuncture Association of Chartered Physiotherapists. 2013;2013(3):25-38.

321. Taylor J. The Role of Complementary Alternative Medicine in Physical Activity and Sport. WellSpring. 2010;21(3):A1-A4.

322. Tekeoglu I, Adak B, Ercan M. Investigation into the possibilities of using ear acupuncture for increasing the pain threshold during athletic training. American Journal of Acupuncture. 1998;26(1):49-52.

323. Tellez-Garcia M, de-la-Llave-Rincon A, Salom-Moreno J, Palacios-Cena M, Ortega-Santiago R, Fernandez-de-las-Penas C. Neuroscience education in addition to trigger point dry needling for the management of patients with mechanical chronic low back pain: A preliminary clinical trial. Journal of Bodywork and Movement Therapies. 2015;19(3):464-72.

324. Tenforde AS, Rhim HC. Evidence-Based Physiatry: Treatment of Midportion Achilles Tendinopathy. American Journal of Physical Medicine & Rehabilitation. 2020;99(12):1189-90.

325. Terada K, Mukai N, Miyamoto T, Miyanaga Y. Effect of acupuncture stimulation on delayed onset muscle soreness produced by eccentric exercise. Japanese Journal of Physical Fitness and Sports Medicine. 2001;50(5):583-91.

326. Thompson R, Prosell M, Timpka T. Elite athletes' experiences of musculoskeletal pain management using neuroanatomical dry needling: A qualitative study in Swedish track and field. Journal of Science & Medicine in Sport. 2021;24(1):46-51.

327. Tkachuk V, Medvedev I, Bachurin E. Effectiveness of acupuncture analgesia in the treatment of chronic postraumatic pain. Orthop Travmatol Protez. 1991(5):33-5.

328. Torcy M. Acupuncture et urgences sportives. Cinesiologie. 1993;32(148):59-60.

329. Trampas A, Kitsios A, Sykaras E, Symeonidis S, Lazarou L. Clinical massage and modified Proprioceptive Neuromuscular Facilitation stretching in males with latent myofascial trigger points. Physical Therapy in Sport. 2010;11(3):91-8.

330. Troop N. Power of the needle. Running Magazine (UK). 1987(69):31-3.

331. Turk Z. Akopunktura pri sportnih poskodbah. / The use of acupuncture in sports injury. Sportnomedicinske Objave. 1981;18(7):256-9.

332. Vander Doelen T, Scott A. Multimodal management of patellar tendinopathy in basketball players: A retrospective chart review pilot study. Journal of Bodywork & Movement Therapies. 2020;24(3):267-72.

333. Vasilenko AM, Doronina YV, Kas'yanov TR, Radzievskii SA, Fisenko LA, Shestkov BP. The adaptogenic effect of paravertebral electropuncture massage during sub-maximal and maximal physical loads. Soviet Sports Review. 1990;25(2):88-9.

334. Vibes J. Les points moteurs des muscles: leur relation avec l'acupuncture. Science & Sports. 1987;2(4):311-2.

335. Volkov VM, Perepekin VA, Nikolaev VA. Moyens de recuperation non traditionnels. Teoria i Praktika Fiziceskoj Kul'tury. 1995(8):22-3.

336. Vonk F, Pool J, Ostelo R, Verhagen A. Physiotherapists' treatment approach towards neck pain and the influence of a behavioural graded activity training: An exploratory study. Manual Therapy. 2009;14(2):131-7.

337. Vrchota K, Belgrade M, Johnson R, Potts J. True acupuncture vs. sham acupuncture and conventional sports medicine therapy for plantar fascitis pain: a controlled, double-blind study. International Journal of Clinical Acupuncture. 1991;2(3):247-53.

338. Wadsorth LT. What does acupuncture do? Sports Medicine in Primary Care. 1998;4(7):57-8.

339. Wadsworth LT. Acupuncture in Sports Medicine. Current Sports Medicine Reports (American College of Sports Medicine). 2006;5(1):1-3.

340. Walsh R, Kinsella S, McEvoy J. The effects of dry needling and radial extracorporeal shockwave therapy on latent trigger point sensitivity in the quadriceps: A randomised control pilot study. Journal of Bodywork & Movement Therapies. 2019;23(1):82-8.

341. Wan H, Du D, Ma Z. Acupuncture at cervical Jiaji (EX B2) combined with motion moxibustion in treating 58 patients with cervical spondylotic radiculopathy. International Journal of Clinical Acupuncture. 2014;23(1):9-10.

342. Wand BM, Abbaszadeh S, Smith AJ, Catley MJ, Moseley GL. Acupuncture applied as a sensory discrimination training tool decreases movement-related pain in patients with chronic low back pain more than acupuncture alone: a randomised cross-over experiment. British Journal of Sports Medicine. 2013;47(17):1085-9.

343. Wang H, He J, Bu M. Clinical study on the treatment of chronic traumatic knee synovitis with blood-letting, cupping and acupuncture. International Journal of Clinical Acupuncture. 2017;26(3):146-9.

344. Wang IL, Chen YM, Jiang YH, Wang J, Chiu WC, Chiu YS. Immediate Effect of Acupuncture on Performance in the Drop Jump Task: A Single-Group Pretest Posttest Experimental Study. European Journal of Integrative Medicine. 2020;36.

345. Wang LQ, Wang AM, Zhang SD. Clinical analysis and experimental observation on acupuncture and moxibustion treatment of patellar tendon terminal disease in athletes. Journal of Traditional Chinese Medicine. 1985;5(3):162-6.

346. Wang X. Early treatment the acute sprain of muscle groups at the back of thigh using electro-acupuncture and massage therapy. Journal of Shandong Physical Education Institute. 1997;13(1):39-41.

347. Wheeler PC, Mahadevan D, Bhatt R, Bhatia M. A Comparison of Two Different High-Volume Image-Guided Injection Procedures for Patients With Chronic Noninsertional Achilles Tendinopathy: A Pragmatic Retrospective Cohort Study. Journal of Foot & Ankle Surgery. 2016;55(5):976-9.

348. White A. Acupuncture greatly improves recovery from spinal cord injury compared with standard care alone. Focus on Alternative and Complementary Therapies. 2003;8(3):336-7.

349. White D. The use of electro-acupuncture in remedial gymnasts. Remedial Gymnastics & Recreational Therapy. 1983;108:9-11.

350. White J. Alternative sports medicine. Physician and Sports Medicine. 1998;26(6):92-105.

351. White J. Alternative sports medicine. / Medecine du sport alternative. Physician & Sportsmedicine. 1998;26(6):92-4;9-100;2;5.

352. White P, Bishop F, Prescott P, Scott C, Little P, Lewith G. Practice, practitioner, or placebo? A multifactorial, mixed-methods randomized controlled trial of acupuncture. Pain. 2012;153(2):455-62.

353. Williams CM, Kamper SJ. Non-specific effects of acupuncture -- does the 'placebo' effect play an important role? British Journal of Sports Medicine. 2012;46(8):578-9.

354. Williams GN, Allen EJ. Rehabilitation of Syndesmotic (High) Ankle Sprains. Sports Health: A Multidisciplinary Approach. 2010;2(6):460-70.

355. Wong CWY, Ng EYL, Fung PW, Mok KM, Yung PSH, Chan KM. Comparison of treatment effects on lateral epicondylitis between acupuncture and extracorporeal shockwave therapy. Asia-Pacific Journal of Sport Medicine Arthroscopy Rehabilitation and Technology. 2017;7:21-6.

356. Wu Z. Acupuncture combined with Chinese medicine in the treatment of 65 cases of cervical vertigo. International Journal of Clinical Acupuncture. 2012;21(4):172-4.

357. Wygand JW, Curley KJ, Sam M, Griffiths K, Henschel M, Katae D, et al. The Effect of Serial Acupuncture Treatment on Anaerobic Power. Medicine & Science in Sports & Exercise. 2011;43:362-.

358. Xiang H. The crura injury and cure in wushu sport. Journal of Wuhan Institute of Physical Education. 1998(3):91-2.

359. Xiao W. Acupuncture in the treatment of soft tissue adhesion from athletic damage. International Journal of Clinical Acupuncture. 1992;3(1):57-66.

360. Xiao W, Zheng F. Treatment of soft tissue adhesion from athletic damage by cross-shaking needling at different level. International Journal of Clinical Acupuncture. 1992;3(4):389-91.

361. Xu T. Acupuncture and cupping in treatment of traumatic chest pain. International Journal of Clinical Acupuncture. 1994;5(2):229-30.

362. Xu XS, Lin WP, Chen JY, Yu LC, Huang ZH. [Efficacy observation on rear thigh muscles strain of athletes treated with surrounding needling of electroacupuncture and hot compress of Chinese medicine]. Zhongguo Zhen Jiu. 2012;32(6):511-4.

363. Xu Y, Wang R. Effects of acupuncture and magnetic acupuncture on the resting potential of skeletal muscles after a long term constriction by electrical stimulation. Sports Science/Tiyu Kexue. 1999;19(1):75-6.

364. Xun H, Zhang Y. Acupuncture combined with blood-letting cupping therapy in treating 40 cases of lumbago of blood stasis type. International Journal of Clinical Acupuncture. 2012;21(4):152-3.

365. Yang J. 32 cases of femoral adductors syndrome treated by electroacupuncture and moxibustion. Journal of Traditional Chinese Medicine. 1998;18(4):263-4.

366. Yang Y, Zhang J. Dong's extra-point acupuncture combined with bloodletting therapy in treating cervical spondylosis of nerve root type. International Journal of Clinical Acupuncture. 2013;22(4):158-9.

367. Yanhui L, Yunshuang Z. Lumbar Muscle Strain Treated by Acupuncture Combined With Ultra Short Wave: 200 Cases. International Journal of Clinical Acupuncture. 2002;13(3):243-4.

368. Yeo A, Kendall N, Jayaraman S. Ultrasound-guided dry needling with percutaneous paratenon decompression for chronic Achilles tendinopathy. Knee Surgery, Sports Traumatology, Arthroscopy. 2016;24(7):2112-8.

369. Zhang F, Miao Y. Acupuncture treatment for sprains of the ankle joint in 354 cases. Journal of Traditional Chinese Medicine. 1990;10(3):207-8.

370. Zhang FC. Analysis of the clinical effect of acupuncture and moxibustion in the treatment of chronic sports soft tissue injuries of shoulder joints. Basic & Clinical Pharmacology & Toxicology. 2020;126:107-8.

371. Zhang HJ, Wang MM, Esa R. Comparison of the Efficacy of Combination and Monotherapy with Acupuncture and Tai Chi-Yunshou against Sports Rotator Cuff Injury2011 2011. 393-5 p.

372. Zhang J. Exercise with acupuncture in treating acute lumbar sprain; a report of 155 cases. International Journal of Clinical Acupuncture. 1995;6(3):343-5.

373. Zhang J. Ashi point massage combined with warming needle acupuncture in treating 40 cases of cervicogenic headache. International Journal of Clinical Acupuncture. 2013;22(3):117-9.

374. Zhang S, Hu B. Galvano-acupuncture for motor injury of the ankle flab: 128 cases. Journal of Hubei Sports Science. 1998;17(4):39-40-5.

375. Zhao Y, Yu NT, Lai ZT. Clinical study of thumb-tack needle therapy for cervical radiculopathy based on meridian differentiation. Journal of Acupuncture and Tuina Science. 2020;18(2):129-34.

376. Zhong J. Acupuncture treatment in 96 cases of superior cluneal nerve injury. J Trad Chin Med. 1991;11(4):259-60.

377. Zhou J, Chen J, Liu J, Zhong Z, Huang Y. Clinical study of JINGJIN therapy compared with normal acupuncture in waist and back muscle fasciitis. International Journal of Clinical Acupuncture. 2014;23(3):118-21.

378. Zhu Z. Electroacupuncture Effects on the Medium - long Distance Athletes' Physical Efficiency Recovery after Half - marathon Race. Journal of Chengdu Sport University. 2007;33(3):83-6.

379. Ziaeifar M, Arab A, Karimi N, Nourbakhsh M. The effect of dry needling on pain, pressure pain threshold and disability in patients with a myofascial trigger point in the upper trapezius muscle. Journal of Bodywork and Movement Therapies. 2014;18(2):298-305.

380. 吴冲云, 杨罗丹, 刘承宜, 朱玲, 段锐, 张全光. 慢性膝关节疼痛针刺治疗的定量差异再分析. / A reanalysis of quantitative differences in the acupuncture therapy of chronic knee joint pain. Journal of Physical Education / Tiyu Xuekan. 2017;24(2):115-21.

381. Quackery1973 1973.

382. Akupunktur im sport. (Acupuncture et sport). Leistungssport. 1974;4(3):218-26.

383. Physiotherapy for Olympic marksmen. Finger-pressure therapy. UIT Shooting Sport Journal. 1981;2:26-7.

384. Acu-Punctured! Joe Weider's Muscle & Fitness. 1999;60(11):28.

385. Acupuncture for archers in field. Physiotherapy Frontline. 2000;6(13):19.

386. Stick It! Joe Weider's Muscle & Fitness. 2000;61(8):162.

387. Acupuncture: this ancient eastern therapy could heal your sports injury and balance your body. Ultra-Fit Magazine. 2001;11(2):80-.

388. Alternative sports medicine: acupuncture, massage. Georgia Tech Sports Medicine & Performance Newsletter. 2002;10(9):4-5.

389. Acupressure for contusions and fractures? Sports Medicine Digest. 2003;25(2):24-.

390. Acupuncture increasingly popular. IDEA Personal Trainer. 2003;14(3):15-.

391. Complementary and alternative medicine in sports. Sports Medicine Digest. 2003;25(2):13-.

392. Sports Med Update. British Journal of Sports Medicine. 2004;38(4):508-10.

393. Acupuncture versus placebo for the treatment of chronic mechanical neck pain. British Journal of Sports Medicine. 2005;39(8):579-.

394. How to Take Strain Off Back Pain. Sports Medicine Bulletin. 2009:6-.

395. What is iliotibial band syndrome? Triathlon Life. 2009;12(1):42-3.

396. Should dry needling for myofascial pain be within the scope of practice for physical therapists? Orthopaedic Physical Therpy Practice. 2011;23(4):212-8.

397. Neck pain: combining exercise and manual therapy for your neck and upper back leads to quicker reductions in pain. J Orthop Sports Phys Ther. 2013;43(3):128.

398. Exercise Tops the List for Knee OA Help. Running & FitNews. 2013;31(3):3-5.

399. Painful and tender muscles: dry needling can reduce myofascial pain related to trigger points muscles. J Orthop Sports Phys Ther. 2013;43(9):635.

400. Acupuncture, miseStrom H en pratique du Daodejing. Acupuncture et Moxibustion. 2014;13(3):188-94.

401. Neck Pain. Journal of Orthopaedic & Sports Physical Therapy. 2014;44(4):261-.

402. The immediate effects of triceps surae myofascial trigger point therapy on restricted active ankle joint doriflexion in recreational runners: A crossover randomized controlled trial. SportEX Dynamics. 2014(39):7-.

403. Untying the knots. Athletics Weekly (0004-6671). 2015:41-.

404. Why and How Acupuncture Works. Running & FitNews. 2016;34(4):13-5.

405. Hip Pain. Journal of Orthopaedic & Sports Physical Therapy. 2017;47(4):240-.

406. Could acupuncture help you running? Athletics Weekly (0004-6671). 2018:8-.

407. East Meets West. Training & Conditioning. 2018;28(3):6-8.

408. Medial tibial stress syndrome (Shin splints) in runners. Co-Kinetic Journal. 2018(77):49-.

409. Acupoint application combined with acupoint massage for ankle sprain of basketball players. Co-Kinetic Journal. 2018(77):9-.

410. The Effect of Muscle Energy Techniques on Latent Trigger Points of the Gastrocnemius Muscle. Sport Journal. 2021

411. Kheradmandi A, Kamali F, Ebrahimian M, Abbasi L. Comparison between dry needling plus manual therapy with manual therapy alone on pain and function in overhead athletes with scapular dyskinesia: A randomized clinical trial. J Bodyw Mov Ther. 2021;26:339-46.

412. Kleinhenz J, Streitberger K, Windeler J, Güssbacher A, Mavridis G, Martin E. Randomised clinical trial comparing the effects of acupuncture and a newly designed placebo needle in rotator cuff tendinitis. Pain. 1999;83(2):235-41

413. Siqueira APR, Beraldo LM, Krueger E, Ulbricht L. Reduction in knee pain symptoms in athletes using an Acupuncture protocol. Acta Ortop Bras. 2018;26(6):418-22.

## eTable 4: Quality assessment for randomized controlled trials

The risk of bias and the quality of the included randomized controlled trials (RCTs) were appraised independently by AJ and KC with the Cochrane Collaboration Risk of bias tool^5^. Each RCT was assigned a low, some concerns, high, or unclear risk of bias (ROB)^5^ for six sources of bias: sequence generation, allocation concealment, blinding of participants and personnel, blinding of outcome assessment, incomplete outcome data, selective outcome reporting^5^. Unclear ROB was attributed to a specific source of bias when the article did not provide enough information to make the decision. Quality assessment was based on information reported in the publications, their supplementary materials, and by contacting the primary study authors when required.

| **Bias Domain** | **Selection Bias** | | **Performance Bias** | **Detection Bias** | **Attrition Bias** | **Reporting Bias** | **Conflict of interest** |
| --- | --- | --- | --- | --- | --- | --- | --- |
| **Sources of Bias** | **Sequence generation** | **Allocation concealment** | **Blinding of participants and personnel** | **Blinding of outcome assessment** | **Incomplete outcome data** | **Selective outcome reporting** |  |
| Ceballos-Laita, 2021^6^ | Low ROB  Quote: "Thirty handball athletes were randomly allocated to both groups: the DN group or the control group. An independent assistant allocated the participants to the groups (ratio 1:1) using the GraphPad computer software 2018  (GraphPad Software, San Diego, CA, USA)." | Unclear ROB  Quote: "An independent assistant allocated the participants to the groups (ratio 1:1) using the GraphPad computer software 2018 (GraphPad Software, San Diego, CA, USA)." | Minor concerns  Single-blind randomized controlled trial  Quote: "Both therapists were blinded to all the measurements."  "The participants remained in prone position for the same time that the intervention lasted in the DN group." | Low ROB  Quote: "Both therapists were blinded to all the measurements.”  "Outcome variables were measured at baseline and immediately after the intervention  by two blinded examiners." | Low ROB  Quote: "No HB athletes were excluded for not presenting an MTrP in the teres major muscle."  " The flowchart of the study is presented in Figure 1."  "No statistically  significant differences were found at baseline between both groups for any of the sociodemographic or clinical variables (p > 0.05)." | Low ROB  All prespecified outcomes were reported | Low ROB  Quote: "This research received no external funding."  "The authors declare no conflict of interest" |
| Etminan, 2019^7^ | Low ROB  Quote: "the participants were divided into two groups (A, B) using simple randomization method and picking up a draw from a box." | Unclear ROB  Not reported | Unclear ROB  Not reported | Unclear ROB  Not reported | Unclear ROB  Not reported | Low ROB  All prespecified outcomes were reported | Low ROB  Quote: "Conflict of interest: None declared" |
| Jamaly, 2018^8^ | Low ROB  Quote: "They randomly allocated into two groups." | Unclear ROB  Not reported | Unclear ROB  Not reported | Unclear ROB  Not reported | Unclear ROB  Not reported | Low ROB  All prespecified outcomes were reported | Low ROB  Quote: "Conflict of interest: None"  "Funding support: None" |
| Kamali, 2019^9^ | Low ROB  Quote: "the patients were randomly allocated into 2 groups of direct DN (needling in the upper trapezius, n = 21) and indirect DN (needling in the infraspinatus, n =  19), by random number table." | Unclear ROB  Not reported | Minor concerns  Single-blind randomized controlled trial  Quote: "The therapist  was also unaware of the patients’ assessment process and treatment  allocations." | Low ROB  Quote: "Outcome measures were assessed at baseline and 3 days after the last treatment session by an educated physical therapist blinded to the treatment procedures."  "The therapist was also unaware of the patients’ assessment  process and treatment allocations." | Low ROB  Quote: "Figure 3 depicts the ﬂow diagram of the study. Demographic characteristics of the participants are summarized in Table 1, and the 2 groups were well-matched at baseline." | Low ROB  All prespecified outcomes were reported | Low ROB  Quote: "The authors have no conflicts of interest to disclose" |
| Lopez-Royo, 2021^10^ | Low ROB  Quote: "Participants were randomly assigned by a researcher to the control  group, DN intervention combined with Ex group, or PNE intervention combined with Ex group with a 1:1:1 allocation." | Low ROB  Quote: "Participants were randomly assigned by a researcher to the control group, DN intervention combined with Ex group, or PNE intervention combined with Ex group with a 1:1:1 allocation using an opaque envelope, with a block size of 15 participants." | Minor concerns  Quote: "A RCT with blinded assessors and participants was conducted to determine whether the addition of either DN or PNE to Ex was superior to sham needling and Ex in patients with PT."   "Assessments were made by an assessor blinded to treatment allocation. In order to blind patients, all interventions were made with the US and PNE device connected to simulate the same intervention in all groups." | Low ROB  Quote: "Assessments were made by an assessor blinded to treatment allocation." | Low ROB  Quote: "Two participants dropped out of the study before starting the intervention."  "Eighteen patients did not complete the blinded questionnaire after the last session, and 2 patients chose “Don’t know.” | Low ROB  All prespecified outcomes were reported | Unclear ROB  None reported |
| Zarei, 2020^11^ | Low ROB  Quote: "This study was a parallel-group, single-blind randomized clinical  trial with an allocation ratio of 1: 1."  "20 participants were randomly assigned to each group. Group assignment was conducted with random allocation software (block size=4, block number= 10) by a biostatistician"  "Female athletes with PFP (N = 40), who were randomly assigned to the exercise therapy (Ex group) or exercise-therapy+ dry needling (Ex+ DN group) group" | Low ROB  Quote: "All allocations were concealed from both the participants and the physiotherapist who administered the  interventions in pre-sealed opaque envelopes. The participants and physiotherapist in each group were blinded to group allocations until the treatments were started." | Minor concerns  Single-blind randomized controlled trial.  "The participants and physiotherapist in each group were blinded to group allocations until the treatments were started. The assessor remained completely blinded to all group allocations" | Low ROB  Quote: "The assessor remained completely blinded to all group allocations" | Low ROB  Quote: "None of the participants dropped out of the study, and all 20 women in each group received the allocated intervention. None of the participants was lost to follow-up, and data for all partici­pants were included in the analysis." | Low ROB  All prespecified outcomes were reported | Unclear ROB  None reported |

DN= Dry needling; Ex= Exercise; PNE= Percutaneous needle electrolysis; PT= Physiotherapy; ROB= Risk of bias; MTrP =Myofascial trigger points

## eTable 5: Quality assessment for Observational, cohort and case-control studies

| **Reference** | Luetmer, 2019^12^ | Garlanger, 2017^13^ |
| --- | --- | --- |
| **Was the research question or objective in this paper clearly stated?** | Yes  Quote: "The aims of this unique study were to primarily assess the feasibility of performing acupuncture on multiple adolescent athletes in a warm weather, high-intensity training environment and to secondarily measure athletes' perceived effects of acupuncture on DOMS and sense of well-being." | Yes  Quote: "To primarily assess the feasibility of performing acupuncture in adolescent Nordic skiers within their athletic environment, and secondarily to measure the effect of acupuncture on muscle soreness and sense of well-being" |
| **Was the study population clearly specified and defined?** | Yes  Quote: "Participants were included if they were between the ages of 13 and 18 yrs, were male or female members of one of two local high school football teams, reported no current musculoskeletal injuries, and were generally healthy individuals who planned to complete the season provided that they did not sustain an injury. Participants were excluded if they had received acupuncture treatment within the past year or had a known active musculoskeletal injury or condition, bleeding disorder, prosthetic heart valve, pregnancy, or immunocompromised state." | Yes  Quote: "Participants were included in this study if they were between the ages of 13 and 18 years, were male or female  members of the local Nordic Ski Team, reported no current musculoskeletal injuries, and were generally healthy individuals who planned to complete the season barring injury or other unexpected events" |
| **Was the participation rate of eligible persons at least 50%?** | NR | NR |
| **Were all the subjects selected or recruited from the same or similar populations (including the same time period)?**  **Were inclusion and exclusion criteria for being in the study prespecified and applied uniformly to all participants?** | Yes  Quote: "Participants were included if they were between the ages of 13 and 18 yrs, were male or female members of one of two local high school football teams, reported no current musculoskeletal injuries, and were generally healthy individuals who planned to complete the season provided that they did not sustain an injury. Participants were excluded if they had received acupuncture treatment within the past year or had a known active musculoskeletal injury or condition, bleeding disorder, prosthetic heart valve, pregnancy, or immunocompromised state" | Yes  Quote: "Participants were included in this study if they were between the ages of 13 and 18 years, were male or female members of the local Nordic Ski Team, reported no current musculoskeletal injuries, and were generally healthy individuals who planned to complete the season barring injury or other unexpected events. Participants were excluded from this study if they had received acupuncture treatment within the past year or had a known active musculoskeletal injury or condition, immunocompromised state, prosthetic heart valve, pregnancy, or bleeding disorder" |
| **Was a sample size justification, power description, or variance and effect estimates provided?** | Yes  Quote: "Recruitment target was 50 participants, which was for the purpose of feasibility" | No |
| **For the analyses in this paper, were the exposure(s) of interest measured prior to the outcome(s) being measured?** | Yes  Quote: "Mean change in pre-post treatment DOMS scores by treatment day is presented in Table 2." | Yes  Quote: "Overall effect of pre to post treatment on muscle soreness over 5 different treatment days is presented in Table 1." |
| **Was the timeframe sufficient so that one could reasonably expect to see an association between exposure and outcome if it existed?** | Yes  Quote: "The secondary purpose was to measure the effects of acupuncture on DOMS and sense of well-being. This was evaluated using the VAS data collected from pretreatment and posttreatment  surveys and analyzed using paired t tests and two-factor analysis of variance with repeated measures on both factors (timing [pretreatment, posttreatment] and study day [day 1, 2, 3, 4, or 5])." | No  Quote: "The lack of significant changes in pre- to posttreatment well-being scores may be attributed to the partial acupuncture treatment protocol. Needling was limited to 3-6 acupuncture points in 1 lower limb. An impact on well-being may be more likely to occur with a complete traditional acupuncture treatment." |
| **For exposures that can vary in amount or level, did the study examine different levels of the exposure as related to the outcome (e.g., categories of exposure, or exposure measured as continuous variable)?** | Yes  Quote: "This was evaluated using the VAS data collected from pretreatment and posttreatment  surveys and analyzed using paired t tests and two-factor analysis of variance with repeated measures on both factors (timing [pretreatment, posttreatment] and study day [day 1, 2, 3, 4, or 5])." | Yes  Quote: "There were a total of 5 treatment days that corresponded to the number of practices in the first 2 weeks of the Nordic Ski team season (Monday through Wednesday of week 1, Monday and Tuesday of week 2) in an attempt to capture potential delayed onset muscle soreness (DOMS) in these athletes." |
| **Were the exposure measures (independent variables) clearly defined, valid, reliable, and implemented consistently across all study participants?** | Yes  Quote: "The length of each treatment including survey and observation time averaged approximately 20 mins. The total time to complete set-up (15 mins), treatments (45 mins for an average of 20–25 participants), and take down (15 mins) for the volunteer, medical, and acupuncture staff was approximately 75mins  per treatment day."  "A minimum of two and maximum of three licensed acupuncturists performed the treatments on different participants for efficiency. The largest participant/acupuncturist  ratio was 7–10:1." | Yes  Quote: "A total of 5 treatment days that corresponded  to the number of practices in the first 2  weeks of the Nordic Ski team season "  "Two licensed acupuncturists were involved in the  treatments over the 5-day study. The mandatory  standards and safety guidelines for acupuncture were  followed; however, there was no fixed treatment protocol  from the literature to follow. The selection of  acupuncture points and location of needle placement  vary across previous studies as the protocols are based  on specific diagnoses." |
| **Was the exposure(s) assessed more than once over time?** | Yes  Quote: "There were a total of five treatment sessions per team; they occurred after afternoon practices during the first 3 wks of the football season in an attempt to capture potential DOMS. Team 1 had treatments on Mondays and Wednesdays and team 2 had treatments on Tuesdays/Thursdays" | Yes  Quote: "The study concluded after a maximum of 5 treatment  days."  "Each treatment day implemented the identical  process described" |
| **Were the outcome measures (dependent variables) clearly defined, valid, reliable, and implemented consistently across all study participants?** | Yes  Quote: "They rated the severity of their muscle soreness and sense of well-being on a 0–10 visual analog scale (VAS). Adolescents have been shown to reliably use numeric VAS to rate their mood and numeric VAS is recommended for measurement of self-reported acute pain in children and adolescents." | Yes  Quote: "The secondary purpose was to measure the effects of acupuncture on muscle soreness and sense of well-being over 5 consecutive practices in the beginning of the Nordic ski season within this population. To address the latter question, visual analogue scale (VAS) data were collected from each participant’s pre- and posttreatment surveys and analyzed using paired t tests and 2-factor analysis of variance with repeated measures on both factors (timing [pretreatment, posttreatment] and study day [day 1, 2, 3, 4, or 5])" |
| **Were the outcome assessors blinded to the exposure status of participants?** | No  Only participant was blinded.  Quote: "One volunteer and a physician were in charge of directing participants to their treatment stations, distributing and collecting participants' deidentified treatment survey packets" | No  Only participant was blinded.  Quote: "De-identified survey packets were given to each participant based on the assigned Participant Code Number." |
| **Was loss to follow-up after baseline 20% or less?** | CD | CD |
| **Were key potential confounding variables measured and adjusted statistically for their impact on the relationship between exposure(s) and outcome(s)?** | No | No  Quote: "we allowed the use of medications  and other conservative treatments between study days to observe whether athletes required fewer additional interventions; unfortunately, this serves as a confounder when analyzing improvements in muscle soreness." |
| **Conflict of interest disclosure** | No  Quote: "Financial disclosure statements have been obtained, and no conflicts of interest have been reported by the authors or by any individuals in control of the content of this article" | No  Quote: All the authors of the study have the disclosure statement "nothing to disclose" |

CD=Cannot determine; NR=Not reported

## eTable 6: Included studies characteristics and study selection for the meta-analyses

| **Reference** | **I** | **C** | **Age** | **Study design** | **Pain location** | **Needle insertion** | **Sex** | **Tool** | **Time of measurement and number of sessions** | **n (I)** | **Mean Pre (I)** | **SD Pre (I)** | **Mean Post (I)** | **SD Post (I)** | **n (C)** | **Mean Pre (C)** | **SD Pre (C)** | **Mean post (C)** | **SD Post (C)** | **Meta-analysis** | |
| --- | --- | --- | --- | --- | --- | --- | --- | --- | --- | --- | --- | --- | --- | --- | --- | --- | --- | --- | --- | --- | --- |
|  |  |  |  |  |  |  |  |  |  |  |  |  |  |  |  |  |  |  |  | **Pre vs. post** | **I vs. C** |
| Ceballos-Laita, 2021^6^ | US guided DN | No I | A | RCT | Shoulder | Muscle | M | NPRS | After the 1st session | 15 | 3.96 | 2.2 | 0.65 | 0.71 | 15 | 3.56 | 2.84 | 3.06 | 2.5 | Incl | Incl |
| Kamali, 2019^9^* | DN | DN | A | RCT | Shoulder | Muscle | M/F | VAS | 3 days after the 3rd session | 21 | 6.4 | 1.77 | 1.73 | 1.31 | NA | NA | NA | NA | NA | Incl | Excl |
| Kamali, 2019^9^* | DN | DN | A | RCT | Shoulder | Muscle | M/F | VAS | 3 days after the 3rd session | 19 | 6.36 | 1.94 | 1.52 | 1.3 | NA | NA | NA | NA | NA | Incl | Excl |
| Luetmer, 2019^12^ | MA | NA | Ado | LS | DOMS | Muscle | M | VAS | Average over the 5 consecutive days of one session each day | 11 | 4.6 | 2 | 2.9 | 2.2 | NA | NA | NA | NA | NA | Incl | Excl |
| Garlanger, 2017^13^ | MA | NA | Ado | LS | DOMS | Muscle | M/F | VAS | Average over the 5 consecutive days of one session each day | 15 | 2.6 | 2.8 | 1.9 | 2.6 | NA | NA | NA | NA | NA | Incl | Excl |
| Etminan, 2019^7^ | DN+PT  +Ex | PT+Ex | A | RCT | Elbow | Tendon | M/F | PREE | After the 4th session | 22 | 34.62 | 9.39 | 18.54 | 12.11 | 22 | 33.9 | 11.17 | 25.18 | 9.65 | Incl | Incl |
| Etminan, 2019^7^ | DN+PT  +Ex | PT+Ex | A | RCT | Elbow | Tendon | M/F | PREE | After the 7th session | 22 | 34.62 | 9.39 | 4 | 5.28 | 22 | 33.9 | 11.17 | 10.18 | 9.55 | Incl | Incl |
| Etminan, 2019^7^ | DN+PT  +Ex | PT+Ex | A | RCT | Elbow | Tendon | M/F | PREE | After the 9th session | 22 | 34.62 | 9.39 | 9 | 7.13 | 22 | 33.9 | 11.17 | 18.4 | 9.21 | Excl | Incl |
| Etminan, 2019^7^ | DN+PT  +Ex | PT+Ex | A | RCT | Elbow | Tendon | M/F | PREE | 1 week after 9 sessions | 22 | 34.62 | 9.39 | 2.63 | 5.58 | 22 | 33.9 | 11.17 | 10.04 | 10.55 | Excl | Incl |
| Lopez-Royo, 2021^10^ | US guided DN+Ex | Sham | A | RCT | Knee | Tendon | M/F | VAS | 2 weeks after 16 sessions | 16 | 3.8 | 1.98 | 2.5 | 0.71 | 16 | 4.3 | 2.11 | 2.3 | 0.61 | Incl | Incl |
| Lopez-Royo, 2021^10^ | US guided DN+Ex | Sham | A | RCT | Knee | Tendon | M/F | VAS | 4 weeks after 16 sessions | 16 | 3.8 | 1.98 | 0.9 | 0.31 | 16 | 4.3 | 2.11 | 1.9 | 0.69 | Excl | Incl |
| Lopez-Royo, 2021^10^ | US guided PNE+Ex | Sham | A | RCT | Knee | Tendon | M/F | VAS | 4 weeks after 16 sessions | 16 | 4.5 | 1.85 | 2 | 0.61 | 16 | 4.3 | 2.11 | 1.9 | 0.69 | Excl | Excl |
| Lopez-Royo, 2021^10^ | US guided PNE+Ex | Sham | A | RCT | Knee | Tendon | M/F | VAS | 2 weeks after 16 sessions | 16 | 4.5 | 1.85 | 2.8 | 0.48 | 16 | 4.3 | 2.11 | 2.3 | 0.61 | Excl | Excl |
| Zarei, 2020^11^ | DN+ Ex | Ex | A | RCT | Knee | Muscle | F | NPRS | A week after the 4 sessions | 20 | 5.9 | 0.85 | 2.1 | 1.11 | 20 | 6 | 0.72 | 4.1 | 0.91 | Incl | Incl |
| Zarei, 2021^11^ | DN+ Ex | Ex | A | RCT | Knee | Muscle | F | NPRS | 2 weeks after the 4 sessions | 20 | 5.9 | 0.85 | 1.4 | 0.82 | 20 | 6 | 0.72 | 3.5 | 1.05 | Excl | Incl |
| Jamaly, 2018^8^ | DN+PT+Ex | PT+Ex | A | RCT | Hip | Muscle | M | VAS | After one session | 20 | 8.41 | NR | 4.2 | NR | 20 | NR | NR | NR | NR | Excl | Excl |

* The control group in this RCT had DN in a shoulder muscle, which was different from the one selected for the intervention group (eTable 6 and eBox 3).

I: Intervention; C: Control; A: Adult; Ado: Adolescent; RCT: Randomized controlled trial; LS: Longitudinal study; M: Male; F: Female; NPRS: Numerical Pain Rating Scale; PREE: Patient rate elbow evaluation questionnaire; VAS: Visual Analog scale; KAKPS: Kujala anterior knee pain scale; DN: Dry Needling; Ex: Exercise therapy; PT: Physiotherapy; NA: Not applicable; NR: Not reported; Incl: Included; Excl: Excluded

## eBox 4: Detailed selection criteria for the meta-analysis

To minimize heterogeneity between studies, data points on the effectiveness of percutaneous needle electrolysis (PNE) were excluded from the meta-analysis^10^. Consequently, only studies reporting the effectiveness of DN or MA were included in the meta-analysis^6,7,9-13^. Pain level mean measured by KAKPS instrument ^11^ was excluded as the study also reported pain levels for the same population using the NPRS instrument, which was used in another study^6^. In one study^9^ the control group received DN in a muscle, which was different from the one selected for the intervention group. Meta-analysis for the pooled difference in mean scores between intervention and control did not include this study(29). However, both the intervention and control groups from the study^9^ were included in the pre/post analysis. Meta-analysis for the pooled difference in pre-post mean scores did not include one study^8^ not reporting standard deviation and 95% confidence interval. When standard deviation was not reported, the values were calculated using 95% confidence interval according to the method recommended by the Cochrane Handbook^14^.

## eTable 7: Characteristics of the studies included in the systematic review

| **Reference** | **Study characteristics** | **Intervention population**  **sample size**  **Mean age (SD)**  **Range**  **Sex: n (%)** | **Intervention** | **Definition of pain** | **Control intervention and population** |
| --- | --- | --- | --- | --- | --- |
| **Randomized Clinical Trials- Adults** | | | | | |
| Ceballos-Laita, 2021^6^ | Spain  Randomized single-blind clinical trial  2021  Male elite professional handball athletes  A minimum of 2 years’ experience practicing handball  Practice routine of a minimum of 2 hrs/day and 3 days/week | 15  22.47y (3.04) M:15 (100) | US guided DN in the teres major muscle  Rationale for treatment: Point selection based on Kietrys, 2013; and Dommerholt, 2006  Treatment variation: NR Number of needles inserted: NR Points used in DN treatment: Active myofascial trigger points in the teres major muscle Insertion depth: NR Response elicited: Hong’s fast-in fast-out technique was performed with the aim of eliciting the local twitch response.  Needle stimulation: NR Retention time: NR Needle size/manufacturer: 0.30 mm × 50 mm single-use stainless needle Number of sessions: One session Frequency/duration of treatment: One session   Details of other intervention: NA  Setting and content of the treatment: An independent assistant allocated the athletes randomly into groups. The DN was performed by the physical therapist and the US was applied to guide the treatment. Both were blinded to all the measurements. The ultrasound-guided technique provided a precise needle location within the teres major muscle, increasing the efficacy of treatment and minimizing risks. The transducer was first placed from the inferior angle of the scapula following the direction of the teres major muscle. The patient was lying in a prone position, and a needle was inserted through the skin beside the ultrasound transducer using a guide tub.  Practitioner background: Experienced physical therapist with more than 7 years of clinical experience in the treatment of myofascial trigger points with DN therapy. The therapist who applied the US had more than 10 years of experience in the musculoskeletal sonography. | Unilateral shoulder pain  Reproducible during throwing actions  A Glenohumeral internal rotation deficit (GIRD value ≥ 15◦)  Presence of an active myofascial trigger points in the teres major muscle. The Travell and Simons criteria were used to identify the presence of myofascial trigger points  External rotation gain suffering with shoulder pain. | No intervention  The participants remained in prone position for the same time that the intervention lasted in the DN group  Sample size:15 Mean age (SD):22.31 (4.37) M:15 (100) |
| Etminan, 2019^7^ | Iran  Randomized clinical trial  Simple sampling  Athletes carrying out exercises that often involve upper limbs (e.g., tennis, badminton, volleyball, and basketball)  At least 3 sessions (exercise or match, 6 hours per week). | 22 35.31y (7.1)  M:6 (27) F:16 (73) | DN in the tendon  Rationale for treatment: Point selection based on Sukumar, 2014; Stenhouse, 2013; and Vermillion, 2013 Treatment variation: Fixed Number of needles inserted: NR Points used in DN: Tendon parallel to skin position and toward the radius bone at the origin of common extensor muscles  Insertion depth: NR Needle stimulation: NR Retention time: 15 mins Needle size/manufacturer: NR Number of sessions: 3 sessions per week Frequency/ duration of treatment: 3 weeks  Details of other intervention:  Physiotherapy Continuous therapeutic ultrasound, one watt, for a period of five minutes at the site of the tendon of muscles of the forearm and the fingers. Deep friction massage for five minutes,   Muscle stretching and strengthening exercise   Setting and content of the treatment: Patients who met the inclusion criteria were randomised either into the dry needling with physiotherapy group or the physiotherapy only group to prove if DN is effective for treating tennis elbow syndrome.   Practitioner background: NR | Tennis elbow syndrome   Cozen’s test was used to ensure that syndrome detection is correct.  Having the syndrome for more than 3 months | Physiotherapy  Continuous therapeutic ultrasound, one watt, for a period of five minutes at the site of the tendon of muscles of the forearm and the fingers  Deep friction massage for five minutes   Muscle stretching and strengthening exercise   Sample size:22 Mean age (SD):34.54y (6.36)  M:1 (4.5) F:21 (95.5) |
| Jamaly, 2018^8^ | Iran  Randomized clinical trial  Simple sampling  Male athletes  Absence of use of steroids and non-steroids   Absence of metabolic and neurologic diseases | 20  26.5y (1.4) 18-30y M:20 (100) | DN in the piriformis muscle  Rationale for treatment: Point selection based on Huguenin, 2005 Treatment variation: NR Number of needles inserted: NR Points used in DN treatment: Tight points or taut band in greater trochanter and sacrum areas in S2, S3 and S4 Insertion depth: NR Response elicited: NR Needle stimulation: NR Retention time: NR Needle size/manufacturer: 0.30mm in diameters and 65mm in length (Seirin Corp, Shizuoka, Japan) Number of sessions: 3 sessions per week Frequency/ duration of treatment: NR  Details of other intervention:  Conventional physiotherapy and stretching exercise Conventional physiotherapy included heat, interferential and ultrasound therapy Stretching exercise consisted of hip flexion 90 degree, hyper horizontal hip adduction and hyper internal hip rotation, 14 repetition and 20-30 seconds for every movement.  Setting and content of the treatment: The patients were allocated randomly into the two groups. DN was performed by a similar examiner.  Practitioner background: NR | Piriformis syndrome   Syndrome and trigger points confirmed by an orthopedist  Positive to at least three of the tests: FAIR, Freiberg, Lasegue, and Beaty tests  Pain in hip or buttock region which is increased by walking, sit to standing and chair climbing and during lower limb functional activities | Conventional physiotherapy  Included heat, interferential and ultrasound therapy  Stretching exercise   Consisted of hip flexion 90-degree, hyper horizontal hip adduction and hyper internal hip rotation, 14 repetition and 20-30 seconds for every movement.  Sample size:20 Mean age (SD):27.6 (5.9) M:20 (100) |
| Kamali, 2019^9^ | Iran  Single-blind randomized clinical trial  Simple random sampling  2018  Overhead athletes (semi-elite throwers, swimmers, volleyball and basketball players)  Suffering from unilateral shoulder impingement syndrome   Referred to sport physiotherapy clinics | 21  27.1y (6.4) M:10 (47.6) F:11 (52.4) | DN in the upper trapezius--Direct DN  Rationale for treatment: Point selection based on Abbaszadeh-Amirdehi, 2017 and Tsai, 2010 Treatment variation: Individualised Number of needles inserted: NR Points used in DN treatment: Myofascial trigger points in the upper trapezius muscle Insertion depth: NR Response elicited: Local twitch response Needle stimulation: MA Retention time: The therapist held the region of the taut band between his thumb and index fingers and repeatedly needled forward and backward to the trigger point until no more local twitch responses occurred. Needle size/manufacturer: 0.2 × 50-mm Number of sessions: 3 Frequency/ duration of treatment: One session every 2 days  Details of other intervention: NA  Setting and content of treatment: The study was carried out in the Sports medicine physical therapy clinic. The patients were randomly allocated into the direct dry needling group or the indirect dry needling group. The physical therapist was unaware of the treatment allocation and assessment process.  Practitioner background: Trained physical therapist | Unilateral shoulder impingement syndrome  Shoulder impingement syndrome was diagnosed by positive Neer and Hawkins clinical tests.  Patients were included if they had active myofascial trigger points in the upper trapezius and infraspinatus muscles.   Active myofascial trigger points were identiﬁed by palpating a taut band with a tender spot in the muscle that reproduced referred pain similar to the patient’s familiar pain in response to compression.   Subjects’ pain intensity should be at least 3 out of 10 on a visual analog scale. | DN in the infraspinatus --Indirect DN   Rationale for treatment: Point selection based on Tsai, 2010 and Chou, 2011 Treatment variation: Individualised Number of needles inserted: NR Points used in DN treatment: Myofascial trigger points in the infraspinatus muscle Insertion depth: NR Response elicited: Local witch response Needle stimulation: MA Retention time: The therapist held the region of the taut band between his thumb and index fingers and repeatedly needled forward and backward to the trigger point until no more local twitch responses occurred. Needle size/manufacturer: 0.2 × 50-mm Number of sessions: 3 Frequency/ duration of treatment: One session every 2 days  Setting of treatment: Sports medicine physical therapy clinic  Practitioner background: Trained physical therapist  Sample size:19  Mean age (SD):26.5y (6.1) M:10 (47.4) F:9 (52.6) |
| Lopez-Royo, 2021^10^ | Spain  Double-blind, randomized controlled trial  Convenience sampling  2019  Participants practicing any kind of sports at least 3 times a week  Recruited from different sports clubs and federations | 16 33.2y (7.97) 19-45.8y M:13 (81) F:3 (19) | US guided DN in the tendon:   Rationale for treatment: NR Treatment variation: Fixed Number of needles inserted: 3 Points used in acupuncture treatment: Application on the injured area guided by ultrasound, (approximately 20 degrees of knee flexion).  Insertion depth: NR Response elicited: NR Needle stimulation: MA Retention time: 3 seconds  Needle size/manufacturer: 0.25 x 25 mm needle/ Agupunt Number of sessions: 4  Frequency/duration of treatment: One session every 2 weeks/8 weeks   Details of other intervention:  Strengthening exercise 3 sets of 15 single-leg squat repetitions on a decline board twice a day, according to Young, 2005 protocols.  Exercise pain was allowed to reach 5 in a numerical pain rating scale.  Setting and content of treatment: Recruitment was performed in sports clubs. All participants were instructed by the physiotherapist on how to do the exercise. All interventions were made with the US and PNE device connected to simulate the same intervention in all groups. The assessments were made by the assessor blinded to treatment allocation.  Practitioner background: Physiotherapist | Patellar tendinopathy  Anterior knee pain located on the inferior pole of the patella for at least 3 months while practicing sport.  Score less than 80 on the Victorian Institute of Sport Assessment-Patellar questionnaire | Sham needling  Needle was placed on the treatment zone, simulating the same procedure undergone by the participants enrolled in the other groups.  Sample size:16  Mean age (SD):32.7y (6.1) Range:20.6-40.2y M :15 (94) F:1(6) |
| Lopez-Royo, 2021^10^ | Spain  Double-blind, randomized controlled trial  Convenience sampling  2019  Participants practicing any kind of sports at least 3 times a week  Recruited from different sports clubs and federations | 16 33.2y (7.97) 19-45.8y M:14 (88) F:2 (12) | US guided PNE in the tendon:   Rationale for treatment: NR Treatment variation: Fixed Number of needles inserted: 3 Points used in acupuncture treatment: Application on the injured area guided by ultrasound (approximately 20 degrees of knee flexion).  Insertion depth: NR Response elicited: NR Needle stimulation: An intensity of 3 mA galvanic current was used during the 3 seconds that the needling procedure lasted Retention time: 3 seconds  Needle size/manufacturer: 0.25 x 25 mm needle/ Agupunt Number of sessions: 4  Frequency/duration of treatment: One session every 2 weeks/8 weeks  Details of other intervention:  Strengthening exercise 3 sets of 15 single-leg squat repetitions on a decline board twice a day, according to Young, 2005 protocols.  Exercise pain was allowed to reach 5 in a numerical pain rating scale.  Setting and content of treatment: Recruitment was performed in sports clubs. All participants were instructed by the physiotherapist on how to do the exercise. All interventions were made with the US and PNE device connected to simulate the same intervention in all groups. The assessments were made by the assessor blinded to treatment allocation.  Practitioner background: Physiotherapist | Patellar tendinopathy  Anterior knee pain located on the inferior pole of the patella for at least 3 months while practicing sport.  Score less than 80 on the Victorian Institute of Sport Assessment-Patellar questionnaire | Sham needle  Needle was placed on the treatment zone, simulating the same procedure undergone by the participants enrolled in the other groups.  Sample size:16  Mean age (SD):32.7y (6.1) Range: 20.6-40.2y M :15 (94) F:1(6) |
| Zarei, 2020^11^ | Iran  Parallel-group, single-blind randomized controlled trial  Convenience sampling  2018  Female athletes doing regular sports activity at least 2h per day 3 times per week | 20 Athletes 22.25y (3.25) F:20 (100) | DN in the Gluteus medius and quadratus lumbarum  Rationale for treatment: Trigger points (TrP) selection based Roach,2013 and Rozenfeld, 2019 Treatment variation: Fixed Number of needles inserted: One Points used: TrP1- Upper lateral quadrant of the buttock between the region proximal to the greater trochanter and inferior to the iliac crest in side lying position TrP2- Region immediately anterior to TrPl in side lying position TrP3- Anterior to the tensor fascia latae muscle by rolling the thumb perpendicular to the muscle fibers inside lying position Insertion depth: NR Response elicited: Local twitch response Needle stimulation: MA Retention time: When the investigator felt the first local twitch response, the needle was moved up/down with no rotation Needle size/manufacturer: 0.30x50-mm and 0.30x 100-mm needles Number of sessions: 4 sessions Frequency/duration of treatment: Once a week for 4 weeks  Details of other intervention:  Exercise 2 sessions of exercise therapy at the clinic and 3 sessions at home per week for 4 weeks An experienced physiotherapist taught the exercises to the participants in each session and supervised them individually to ensure that the exercises were done correctly. However, the home exercise program was not supervised.   Setting and content of treatment: In the study one group received the exercise therapy and the other group received the same exercise therapy protocol in combination with dry needling. When necessary, the exercise therapy was tailored based on individual symptoms and abilities.  Practitioner background: An experienced physiotherapist carried out the Acupuncture and taught the exercise | Unilateral prepatellar or retropatellar pain   Unrelated to trauma for at least 3 months, aggravated with at least 2 daily activities such as prolonged sitting, kneeling, squatting, running, hopping, or stair climbing  Positive Clarke's sign  Average pain in the previous week >3 on numeric pain rating scale  Kujala anterior knee pain scale <85 of 100   Trigger points in the gluteus medius on the affected side and in quadratus lumbarum on the nonaffected side | Exercise  2 sessions of exercise therapy at the clinic and 3 sessions at home per week for 4 weeks.  An experienced physiotherapist taught the exercises to the participants in each session and supervised them individually to ensure that the exercises were done correctly. However, the home exercise program was not supervised.  Sample size:20  Mean age (SD):25.65y (8.49) F:20 (100) |
| **Longitudinal Studies- Adolescents** | | | | | |
| Garlanger, 2017^13^ | USA  Prospective (feasibility) observational study (no randomization, blinding, or long-term follow-up)  2016  Healthy male and female adolescent ski athletes of the local Nordic Ski Team with no current musculoskeletal injuries | 15 Range:14-17y M:3 (20) F:12 (80) | MA in the muscle in the lower limbs  Rationale for treatment: "No fixed treatment protocol from the literature to follow due to selection of acupuncture points and location of needle placement vary across previous studies because the protocols are based on specific diagnoses" Treatment variation: Partially individualized Number of needle insertions: NR Points used in MA treatment: Acupuncture points specific to muscle groups in the lower limbs which are commonly painful during Nordic skiing were chosen. These muscles (and their corresponding acupoint names) included the quadriceps (points SP10 and ST34), anterior tibialis (anterior shin point ST36), gastrocnemius-soleus complex (posterior calf point BL56), medial head of the gastrocnemius (posterior medial calf point SP9), and peroneal muscles (lateral leg point GB34). Insertion depth: 1/8 inch to 1 inch Response elicited: No twitch responses were intentionally stimulated, but sometimes occurred spontaneously. Needle stimulation: Manual stimulation moving needle with up and down movements.  Retention time: 15 minutes Needle size/length/type/manufacturer: 0.2mm diameter, no. 36 gauge, 1-inch stainless steel, single-use, disposable needles Number of sessions: 5 sessions Frequency/duration of treatment sessions: One session per day for 5 days  Details of other intervention: None  Setting and content of treatment: Indoor or outdoor treatment stations dependent on weather conditions. The participants were given de-identified survey packets and assigned a participation code number.   Practitioner background: 2 licensed acupuncturists | Delayed-onset muscle soreness   Pain arising from exercise induced muscle soreness in the beginning of the Nordic ski season | NA |
| Luetmer, 2019^12^ | USA  Prospective (feasibility) observational study (no randomization, blinding, or long-term follow-up)  2018  Healthy male and female high school football team players with no reported current musculoskeletal injuries | 11 Mean:16y Range:13-18y M:11 (100) | MA in the muscles in the lower limbs  Rationale for treatment: Study used acupuncture points specific to muscle groups in the lower limbs, which are heavily exercised in football Treatment variation: Partially individualised Number of needles inserted: NR Point used in MA treatment: Acupuncture points specific to muscle groups in the lower limbs, which are heavily exercised in football. These muscles (and their corresponding acupoint names) included the quadriceps (points SP10 and ST34), anterior tibialis (anterior shin point ST36), gastrocnemius-soleus complex (posterior calf point BL56), medial head of the gastrocnemius (posterior medial calf point SP9), and peroneal muscles (lateral leg point GB34). Insertion depth:1/8 inch to 1 inch Response elicited: Twitch responses were not intentionally elicited but sometimes occurred spontaneously. Needle stimulation: Needling was “augmented” by incorporating “manual stimulation,” such as gentle twisting of the needles back and forth with up and down needle movements. Retention time: 15 minutes Needle size/manufacturer: 0.2-mm diameter, no. 36 gauge, 1-in stainless steel, single-use, disposable needles; Seirin Corporation Number of sessions: 5 sessions  Frequency/ duration of treatment: One session per day for 5 days  Details of other intervention: None  Setting and content of treatment: A minimum of two and maximum of three licensed acupuncturists performed the treatments on different participants for efficiency. The largest participant/acupuncturist ratio was 7–10:1. One volunteer and a physician were in charge of directing participants to their treatment stations, distributing and collecting participants' deidentified treatment survey packets, and monitoring for adverse effects. The participant remained in a chair, limiting body movement as much as possible, before and during treatment as well as during resting time to provide an optimal environment for therapeutic effect.   Practitioner background: Acupuncturists master level licensed, board certified by the National Certification Commission for Acupuncture and Oriental Medicine, and had similar levels of clinical experience | Delayed-onset muscle soreness   Soreness or pain in large muscle groups, used during football practice, described by the athletes during the three first weeks of the football season. The pain was specific to muscle groups rather than joints. | NA |

MA=Manual Acupuncture; DN= Dry Needling; Ex= Exercise therapy; Pt= Physiotherapy; SM= Scapular mobilization; PNE= Percutaneous needle electrolysis; PREE= Patient rate elbow evaluation questionnaire; PNE= Percutaneous needle electrolysis; KAKPS= Kujala anterior knee pain scale; NA= Not applicable; NR=No reported

## eTable 8: Acupuncture effectiveness reported in the included studies

| **Reference** | **Intervention** | **Control** | **Instrument administered** | **Measurement time of intervention efficacy** | **Pain level (mean pain score (SD); range)** | | | | **Reported mean difference**  **(SD; [95%CI]; p-value)** | |
| --- | --- | --- | --- | --- | --- | --- | --- | --- | --- | --- |
|  |  |  |  |  | **Pre (I)** | **Post (I)** | **Pre (C)** | **Post (C)** | **Pre/Post (I)** | **Pre/Post (C)** |
| Ceballos-Laita, 2021^6^ | US guided DN | No intervention | NPRS | After the 1st session | 3.96 (2.20) | 0.65 (0.71) | 3.56 (2.84) | 3.06 (2.50) | −3.30 (NR); [NR]; p-value=<0.001 | −0.50 (NR); [NR]; p-value = 0.072 |
| Etminan, 2019^7^ | DN+ PT+ Ex | PT+ Ex | PREE | After the 4th session | 34.62 (9.39) | 18.54 (12.11) | 33.90 (11.17) | 25.18 (9.65) | -16.08* (NR); [NR] | -8.72 (3.91); [NR] |
|  |  |  |  | After the 7th session | 34.62 (9.39) | 9.00 (7.13) | 33.90 (11.17) | 18.40 (9.21) | -25.62* (NR); [NR] | -15.50 (5.8); [NR] |
|  |  |  |  | After the 9th session | 34.62 (9.39) | 2.63 (5.58) | 33.90 (11.17) | 10.04 (10.55) | -31.99* (NR); [NR] | -23.86 (8.23); [NR] |
|  |  |  |  | One week after 9 sessions | 34.62 (9.39) | 4.00 (5.28) | 33.90 (11.17) | 10.18 (9.55) | -30.62* (NR); [NR] | -23.72 (8.49); [NR] |
| Jamaly, 2018^8^ | DN+ PT+ Ex | PT+ Ex | VAS | After session one | 8.41 (NR) | 4.2 | NR | NR | NR | NR |
| Kamali, 2019^9^ | DN | DN | VAS | 3 days after the 3rd session | 6.40 (1.77) | 1.73 (1.31) | 6.36 (1.94) | 1.52 (1.30) | -4.66 (2.01); [NR];  p-value=<.001 | -4.84 (1.74); [NR];  p-value=<.001 |
| Lopez-Royo, 2021^10^ | US guided DN+ Ex | Sham | VAS | 2 weeks after 16 sessions | 3.8 (1.98);  0-7 | 2.5; 1.1-3.9 | 4.3 (2.11);  1-8 | 2.3;  1.1 - 3.5 | -1.31 (NR); [-2.85; -0.23]; p-value=.12 | -1.97 (NR); [-3.56; -0.38]; p-value=.01 |
|  |  |  |  | 4 weeks after 16 sessions | 3.8 (1.98);  0-7 | 0.9; 0.3-1.5 | 4.3 (2.11);  1-8 | 1.9; 0.5-3.2 | -2.91 (NR); [-4.25; -1.57];  p- value=<.01 | -2.4 (NR); [-3.78; -1.02]; p-value=<.01 |
| Lopez-Royo, 2021^10^ | US guided PNE+ Ex | Sham | VAS | 2 weeks after 16 sessions | 4.5 (1.85);  2-7 | 2.8; 1.8-3.7 | 4.3 (2.11);  1-8 | 2.3; 1.1-3.5 | -1.75 (NR); [-3.29; -0.21];  p- value=.02 | -1.97 (NR); [-3.56; -0.38]; p-value=<.01 |
|  |  |  |  | 4 weeks after 16 sessions | 4.5 (1.85); 2-7 | 2; 0.8-3.2 | 4.3 (2.11); 1-8 | 1.9; 0.5-3.2 | -2.53 (NR); [-3.87; -1.19];  p- value=<.01 | -2.4 (NR); [-3.78; -1.02]; p-value=<.01 |
| Zarei, 2020^11^ | DN+ Ex | Ex | NPRS | A week after the 4 sessions | 5.90 (0.85) | 2.10 (1.11) | 6.00 (0.72) | 4.10 (0.91) | -3.80 (0.61); [NR] | -1.90* (NR); [NR] |
|  |  |  |  | 2 weeks after the 4 sessions | 5.90 (0.85) | 1.40 (0.82) | 6.00 (0.72) | 3.50 (1.05) | -4.50 (0.76); [NR] | -2.5* (NR); [NR] |
|  |  |  |  | 6th-4th week | 2.10 (1.11) | 1.40 (0.82) | 4.10 (0.91) | 3.50 (1.05) | -0.70 (0.86); [NR] | -0.6* (NR); [NR] |
| Zarei, 2020^11^ | DN+ Ex | Ex | KAKPS | A week after the 4 sessions | 73.95 (6.41) | 86.00 (5.31) | 72.80 (6.52) | 78.20 (5.94) | 12.05 (3.45); [NR] | 5.4* (NR); [NR] |
|  |  |  |  | 2 weeks after the 4 sessions | 73.95 (6.41) | 92.10 (4.41) | 72.80 (6.52) | 80.75 (6.06) | 18.15 (3.91); [NR] | 7.95* (NR); [NR] |
|  |  |  |  | 6th-4th week | 86.00 (5.31) | 92.10 (4.41) | 72.80 (6.52) | 80.75 (6.06) | 6.10 (3.37); [NR] | 2.55* (NR); [NR] |
| Garlanger, 2017^13^ | MA | NA | VAS | Average over the 5 consecutive days of one session each day | 2.6 (2.8); 0-9 | 1.9 (2.6); 0-1 | NA | NA | NR | NA |
| Luetmer, 2019^12^ | MA | NA | VAS | Average over the 5 consecutive days of one session each day | 4.6 (2.0); 0-10 | 2.9 (2.2);  0-10 | NA | NA | −1.68 (1.33); [NR] | NA |

*The mean difference was calculated using raw data

US: Ultrasound therapy; MA: Manual Acupuncture; DN: Dry needling; Ex: Exercise; SM: Scapular Mobilization; PT: Physiotherapy;

VAS: Visual Analog Scale; NPRS: Numerical Pain Rating Scale; PREE: Patient Rate Elbow Evaluation questionnaire; KAKPS: Kujala Anterior Knee Pain Scale; PNE: Percutaneous needle electrolysis; NA: Not Applicable; NR: Not Reported

## eFigure 1: Meta-analysis for the assessment of the pooled pre-to-post intervention difference in pain mean scores (MD) difference by pain etiology


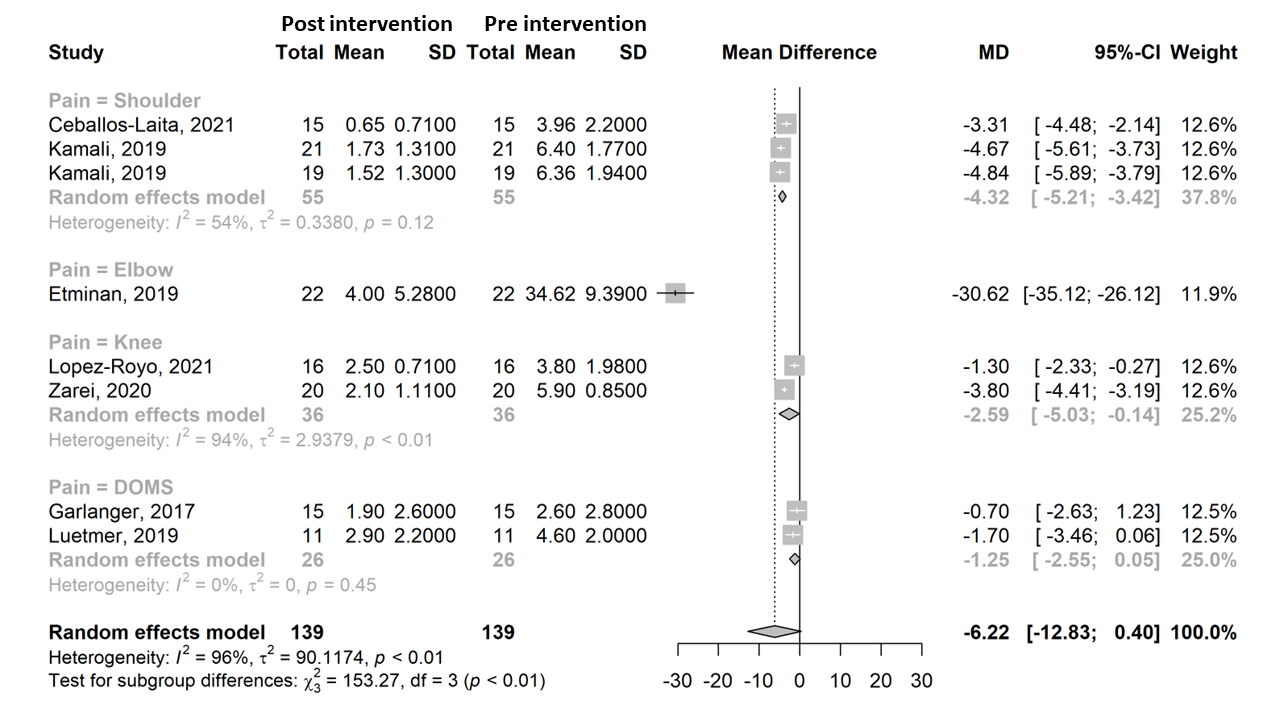


## eFigure 2: Meta-analysis for the assessment of the pooled pre-to-post intervention difference in pain mean scores (MD) difference by pain measurement instrument


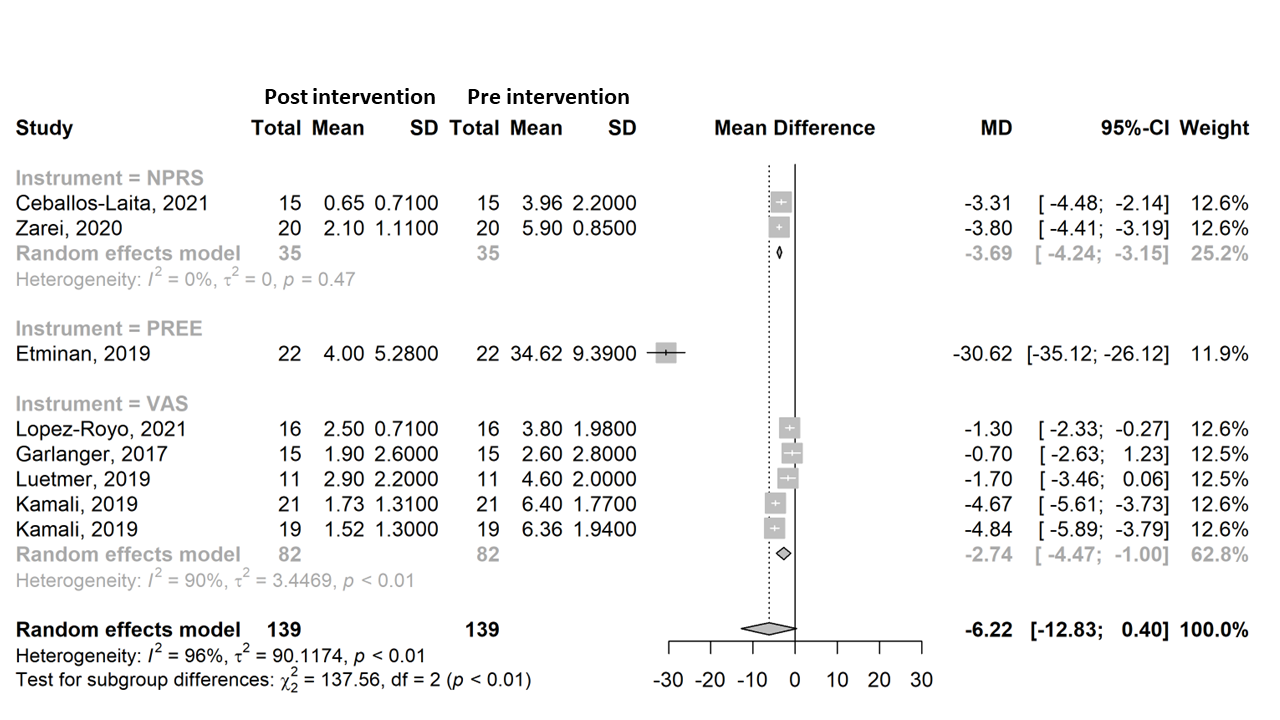


NPRS: Numerical Pain Rating Scale; PREE: Patient Rate Elbow Evaluation questionnaire; VAS: Visual Analog Scale. Zarei (2020)[26] reported pain outcomes using both the Kujala Anterior Knee Pain Scale (KAKPS) and the Numeric Pain Rating Scale (NPRS); however, only NPRS data were included in the meta-analysis

## eFigure 3: Meta-analysis for the assessment of the pooled pre-to-post intervention difference in pain mean scores (MD) difference by number of sessions


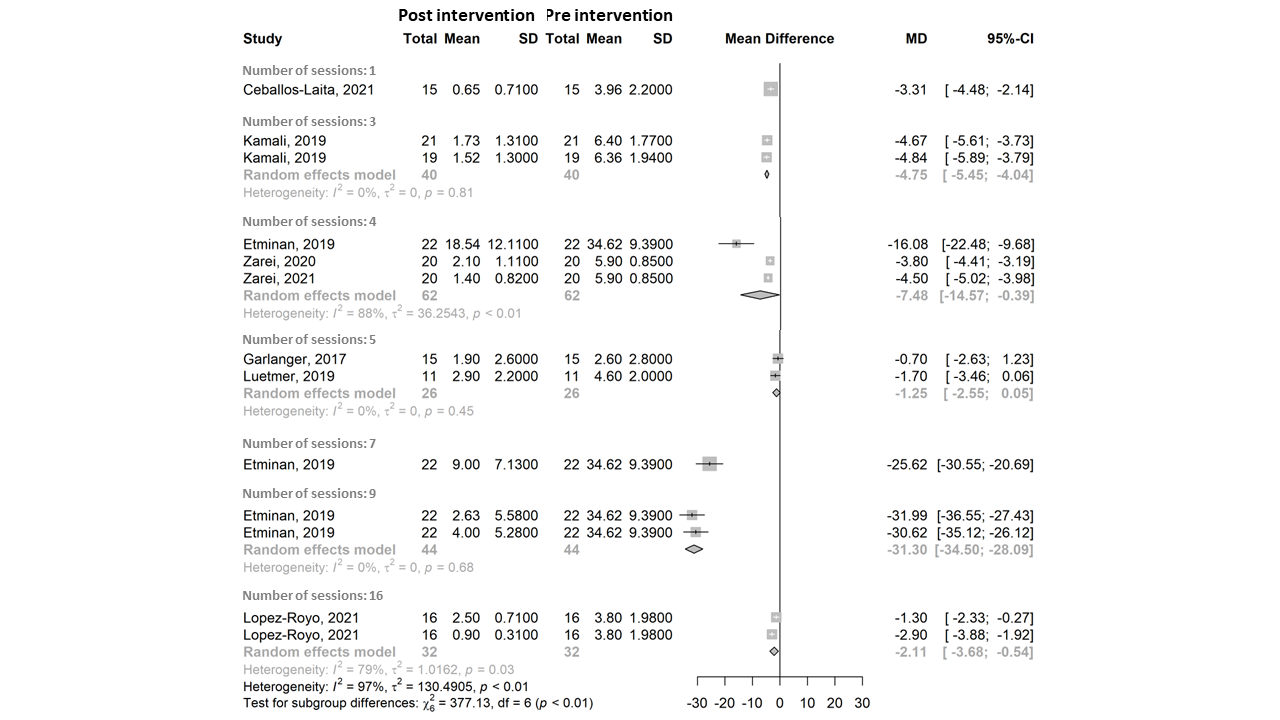


## eFigure 4: Meta-analysis for the assessment of the pooled pre-to-post intervention difference in pain mean scores (MD) difference by body section studied


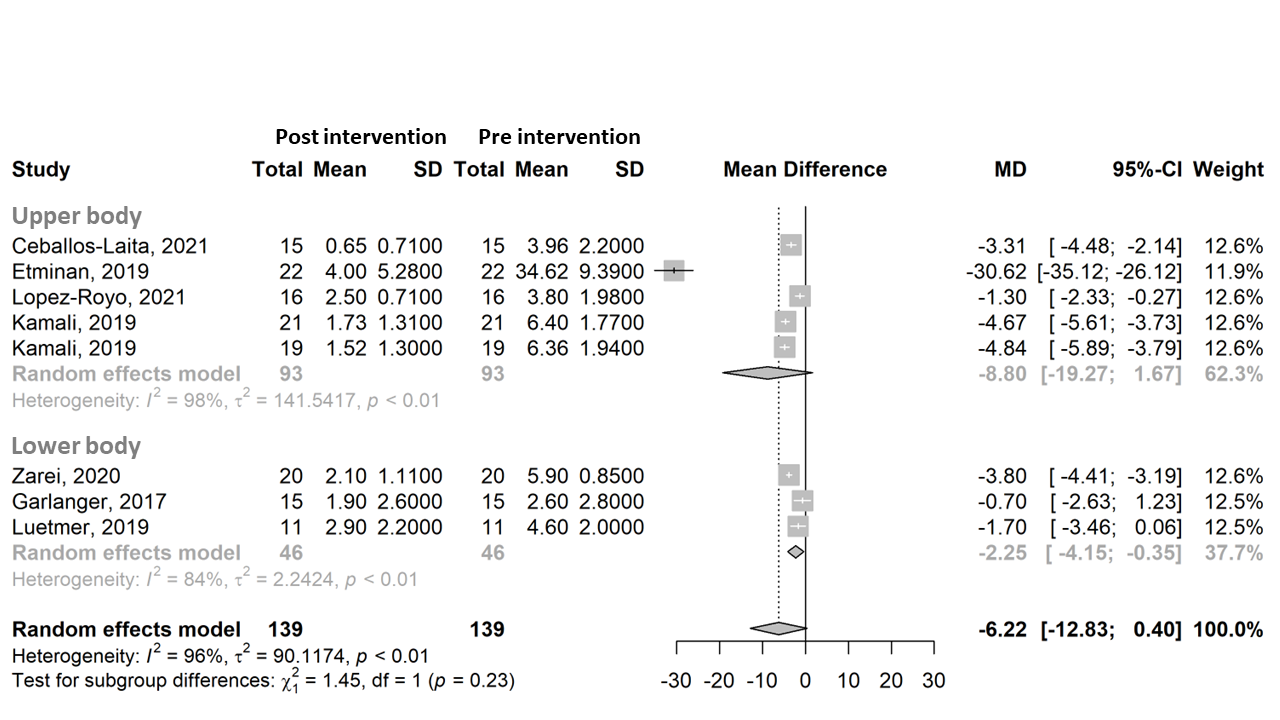


## eFigure 5: Meta-analysis for the assessment of the pooled pre-to-post intervention difference in pain mean scores (MD) difference by needle insertion location


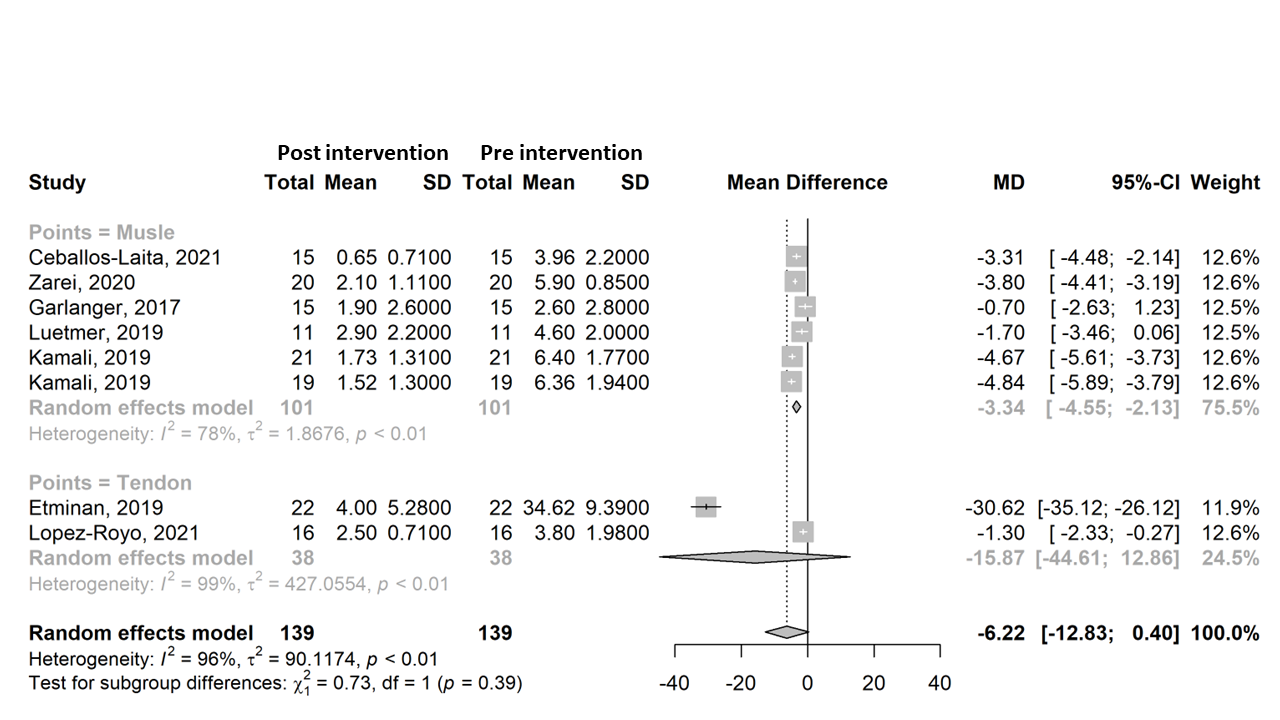


## eFigure 6: Meta-analysis for the assessment of the pooled difference in mean scores (MD) between the intervention vs. the control groups by type of control intervention


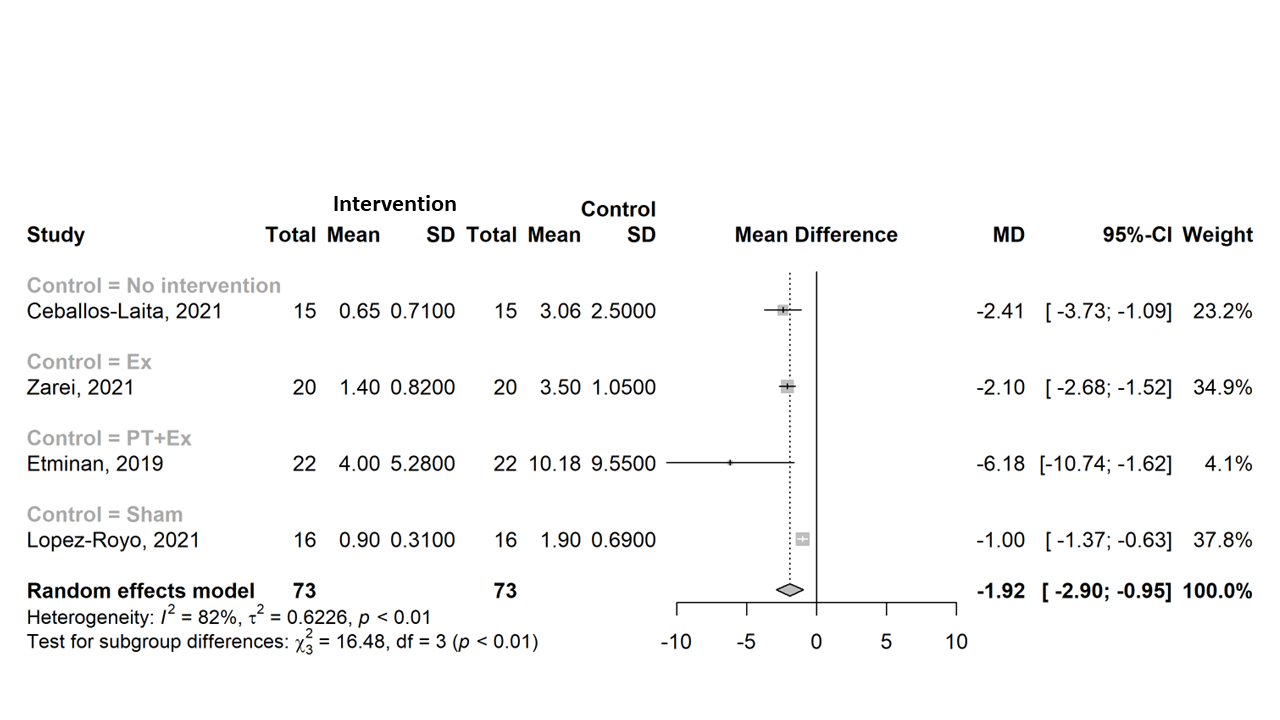


## eFigure 7: Meta-analysis for the assessment of the pooled difference in mean scores (MD) between the intervention vs. the control groups by pain measurement instrument

**
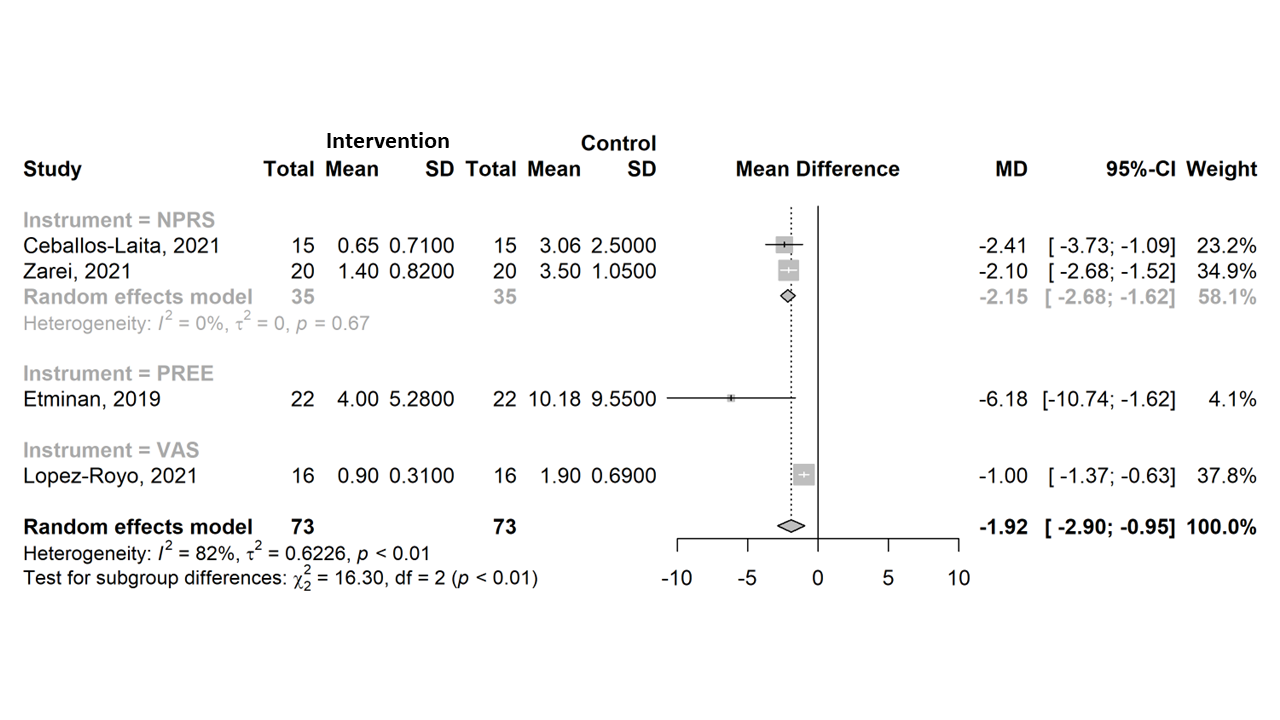
**

NPRS: Numerical Pain Rating Scale; PREE: Patient Rate Elbow Evaluation questionnaire; VAS: Visual Analog Scale

## eFigure 8: Meta-analysis for the assessment of the pooled difference in mean scores (MD) between the intervention vs. the control groups by number of sessions
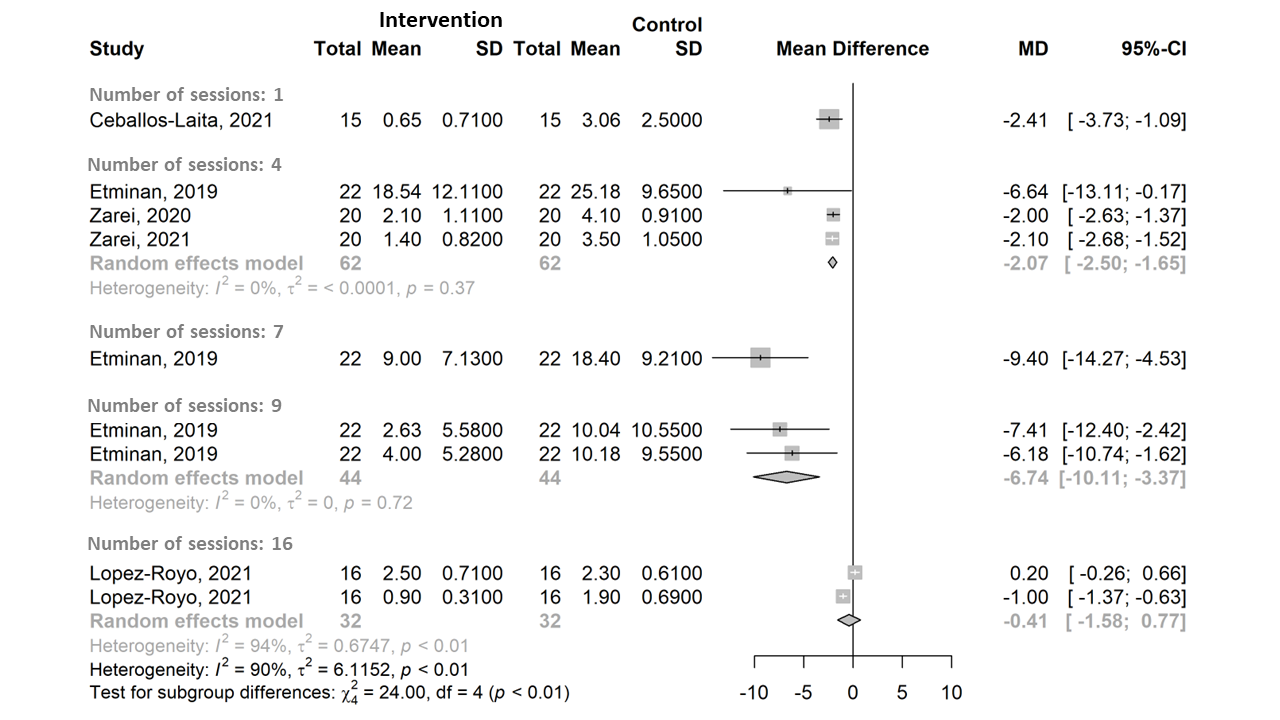


## eFigure 9: Meta-analysis for the assessment of the pooled difference in mean scores (MD) between the intervention vs. the control groups by pain etiology


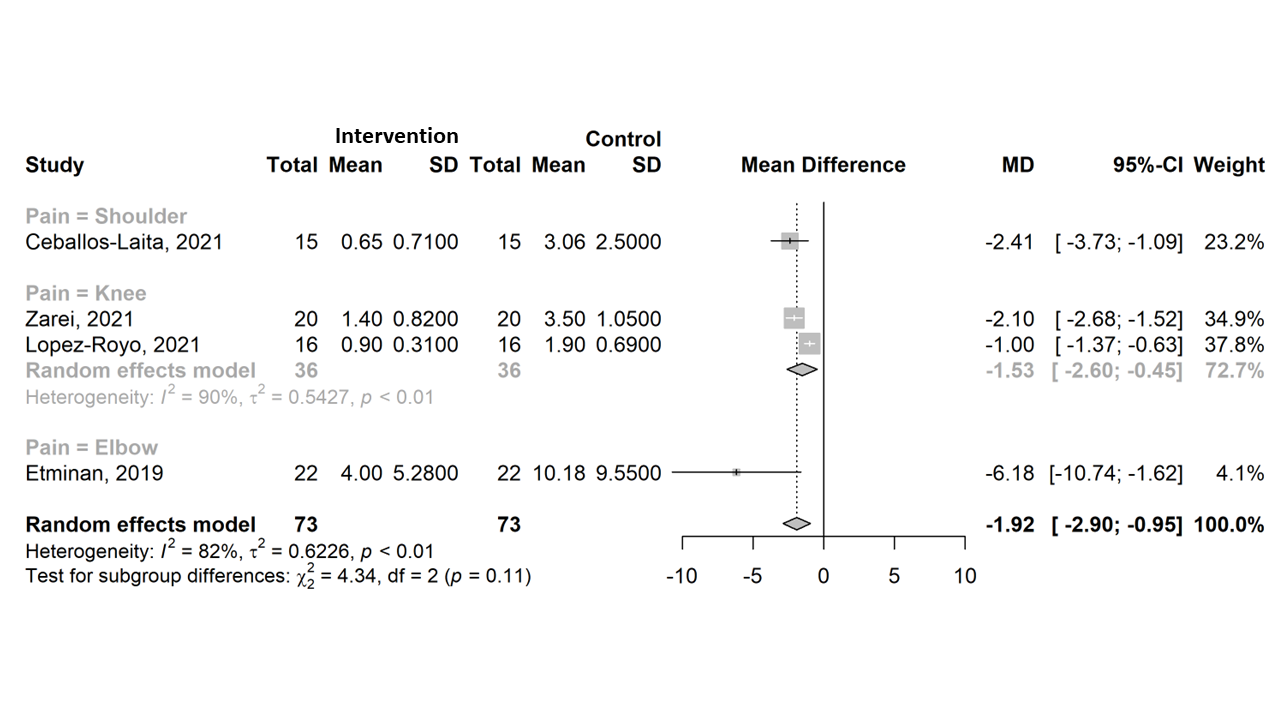


## eFigure 10: Meta-analysis for the assessment of the pooled difference in mean scores (MD) between the intervention vs. the control groups by body section studied


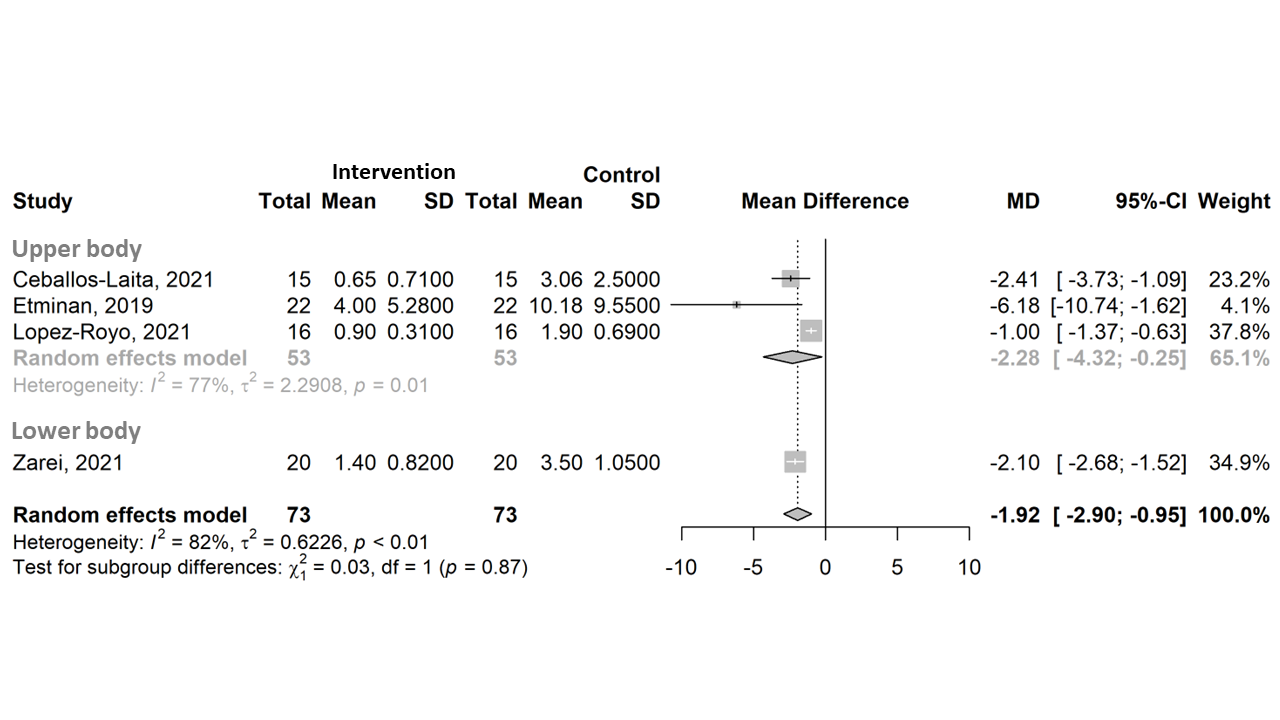


## eFigure 11: Meta-analysis for the assessment of the pooled difference in mean scores (MD) between the intervention vs. the control groups by needle insertion location
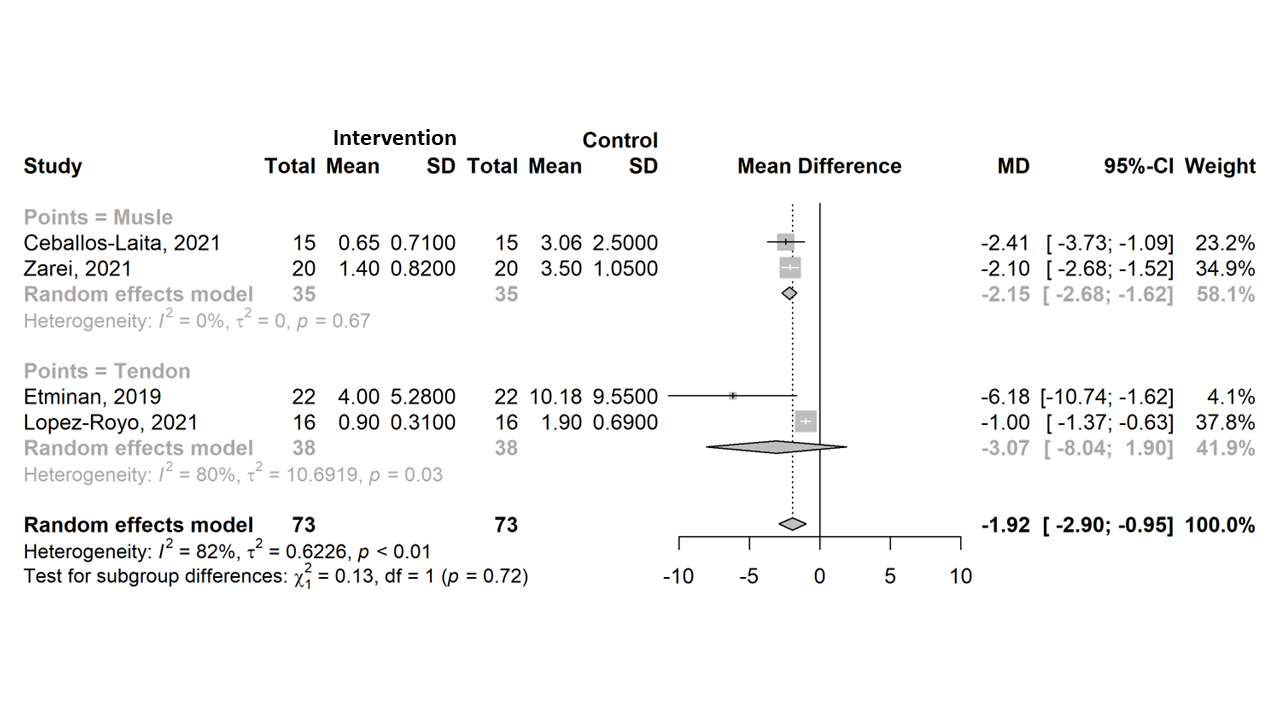


## eFigure 12: Funnel plot for the pre-post mean difference meta-analysis


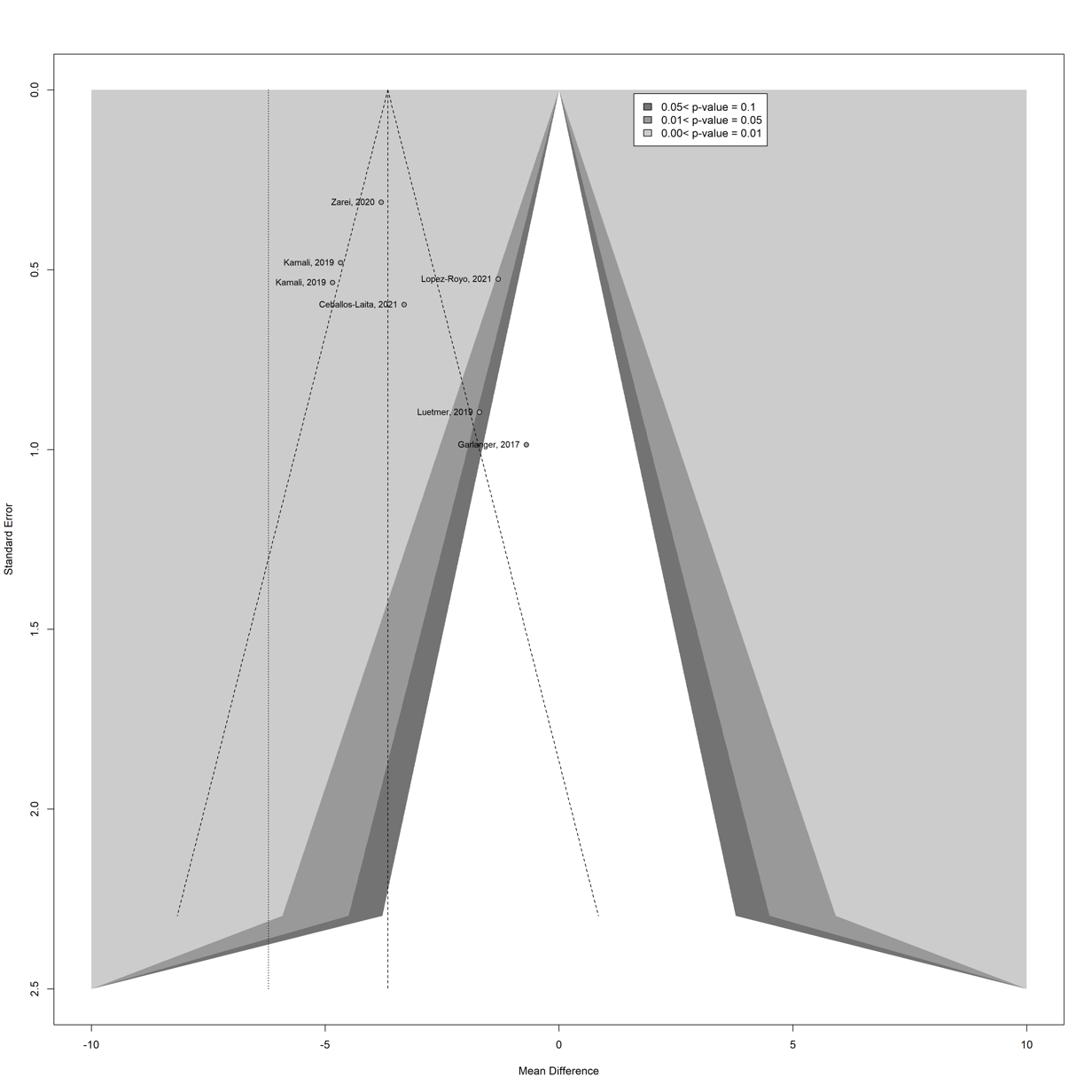
Contour enhanced funnel plots. In the pre–post analyses, visual inspection suggested asymmetry. There is a suggestion of missing studies in the right of the plot, broadly in the white area of non-significance, making publication bias plausible. We were unable to apply Egger’s test since each meta-analysis included fewer than ten data points,

## eFigure 13: Funnel plot for the meta-analysis of mean difference between the intervention and control groups

**
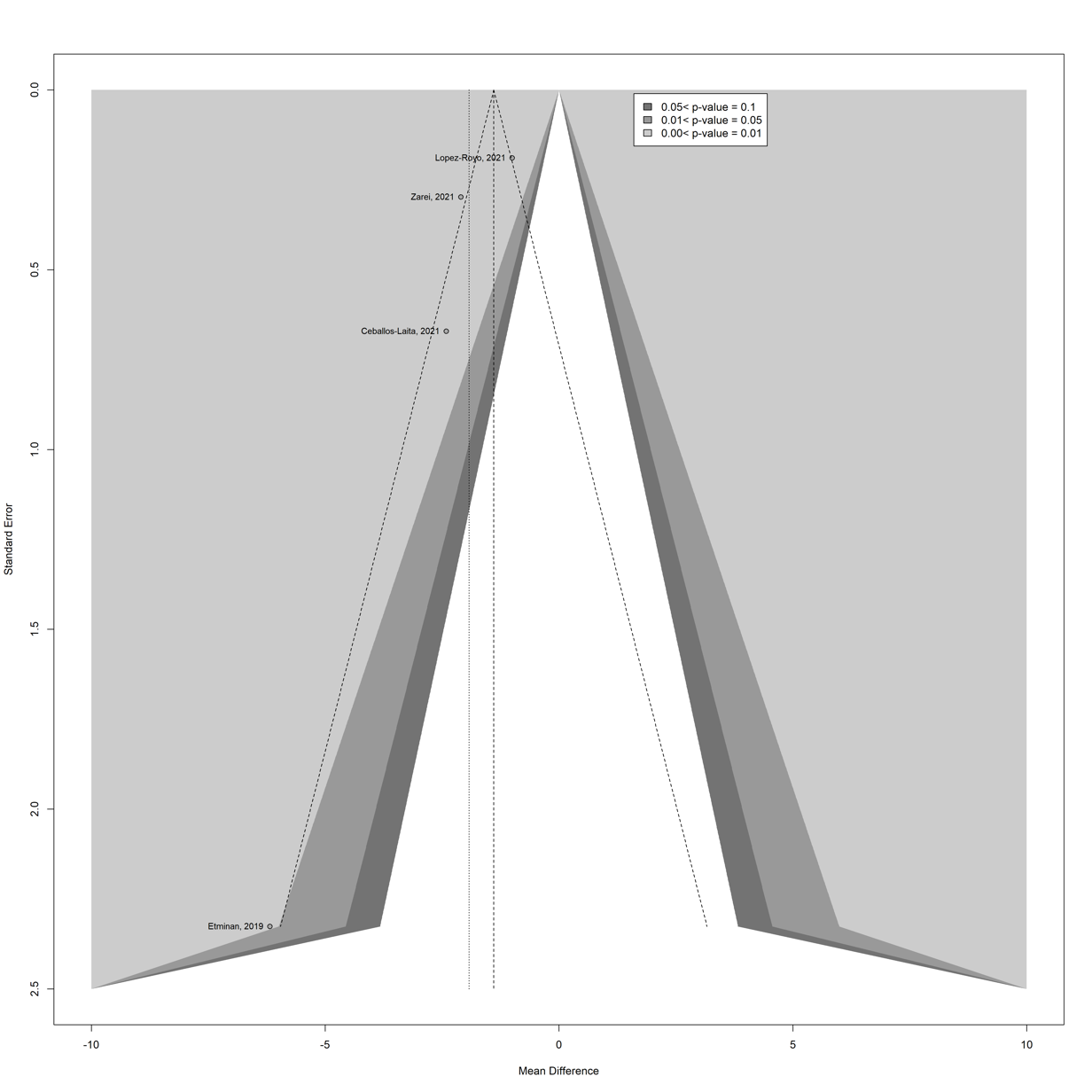
**

In the intervention vs. control analyses, the funnel plot revealed missing studies on the right-hand side, particularly within the nonsignificant white contour area, suggesting potential publication bias. We were unable to apply Egger’s test since each meta-analysis included fewer than ten data points.

## Supplemental Reference

1. White A. Western medical acupuncture: a definition. *Acupunct Med.* 2009;27(1):33-35.

2. Hotfiel T, Freiwald J, Hoppe MW, et al. Advances in Delayed-Onset Muscle Soreness (DOMS): Part I: Pathogenesis and Diagnostics. *Sportverletz Sportschaden.* 2018;32(4):243-250.

3. Chang WD, Chang NJ, Lin HY, Wu JH. Effects of Acupuncture on Delayed-Onset Muscle Soreness: A Systematic Review and Meta-Analysis. *Evid Based Complement Alternat Med.* 2020;2020:5864057.

4. Cheung K, Hume P, Maxwell L. Delayed onset muscle soreness : treatment strategies and performance factors. *Sports Med.* 2003;33(2):145-164.

5. Higgins JP, Altman DG, Gøtzsche PC, et al. The Cochrane Collaboration's tool for assessing risk of bias in randomised trials. *Bmj.* 2011;343:d5928.

6. Ceballos-Laita L, Medrano-de-la-Fuente R, Estébanez-De-Miguel E, et al. Effects of Dry Needling in Teres Major Muscle in Elite Handball Athletes. A Randomised Controlled Trial. *J Clin Med.* 2021;10(18).

7. Etminan Z, Razeghi M, Ghafarinejad F. The effect of dry needling of trigger points in forearm’s extensor muscles on the grip force, pain and function of athletes with chronic tennis elbow. *Journal of Rehabilitation Sciences & Research.* 2019;6(1):27-33.

8. Jamaly A, Mohsenifar H, Amiri A. The effects of dry needling in combination with physical therapy on improvement of pain and hip internal rotation range in patients with piriformis syndrome. *Journal of Clinical Physiotherapy Research.* 2018;3(3):118-122.

9. Kamali F, Sinaei E, Morovati M. Comparison of Upper Trapezius and Infraspinatus Myofascial Trigger Point Therapy by Dry Needling in Overhead Athletes With Unilateral Shoulder Impingement Syndrome. *Journal of Sport Rehabilitation.* 2019;28(3):243-249.

10. López-Royo MP, Ríos-Díaz J, Galán-Díaz RM, Herrero P, Gómez-Trullén EM. A Comparative Study of Treatment Interventions for Patellar Tendinopathy: A Randomized Controlled Trial. *Archives of Physical Medicine & Rehabilitation.* 2021;102(5):967-975.

11. Zarei H, Bervis S, Piroozi S, Motealleh A. Added Value of Gluteus Medius and Quadratus Lumborum Dry Needling in Improving Knee Pain and Function in Female Athletes With Patellofemoral Pain Syndrome: A Randomized Clinical Trial. *Archives of Physical Medicine & Rehabilitation.* 2020;101(2):265-274.

12. Luetmer MT, Do A, Canzanello NC, Bauer BA, Laskowski ER. The Feasibility and Effects of Acupuncture on Muscle Soreness and Sense of Well-being in an Adolescent Football Population. *American Journal of Physical Medicine & Rehabilitation.* 2019;98(11):1-7.

13. Garlanger KL, Fredericks WH, Do A, Bauer BA, Laskowski ER. The Feasibility and Effects of Acupuncture in an Adolescent Nordic Ski Population. *Pm r.* 2017;9(8):795-803.

14. Higgins JPT TJ, Chandler J, Cumpston M, Li T, Page MJ, Welch VA (editors). Cochrane Handbook for Systematic Reviews of Interventions version 6.3. Cochrane. [www.training.cochrane.org/handbook](file:///C:\Users\kac2047\Box\IPH-Internal%20Projects\Acupuncture\Effectiveness%20manuscript\Manuscript\Submission\4-Scientific%20Reports\www.training.cochrane.org\handbook). Published 2022. Accessed October, 2021.
